# Long Noncoding RNA Interleukin 6 Antisense RNA 1 Promotes Inflammatory Effects in Lung Macrophages via Exosomes Through the S100A9/TLR4 Pathway in Chronic Obstructive Pulmonary Disease Progression

**DOI:** 10.1002/mco2.70204

**Published:** 2025-06-06

**Authors:** Erkang Yi, Xiaoyu Wang, Yu Liu, Zihui Wang, Ge Bai, Xinyue Mei, Fan Wu, Chengshu Xie, QiYang Li, Weitao Cao, Huahua Xu, Xinyuan Liu, Jieda Cui, Haiqing Li, Ruiting Sun, Xinru Ran, Wei Hong, Zhishan Deng, Bing Li, Yumin Zhou, Pixin Ran

**Affiliations:** ^1^ State Key Laboratory of Respiratory Disease National Clinical Research Center for Respiratory Disease Guangzhou Institute of Respiratory Health The First Affiliated Hospital of Guangzhou Medical University Guangzhou Medical University Guangzhou Guangdong China; ^2^ Guangzhou National Laboratory. Guangzhou International BioIsland Guangzhou Guangdong China; ^3^ GMU‐GIBH Joint School of Life Sciences Guangzhou Medical University Guangzhou Guangdong China

**Keywords:** chronic obstructive pulmonary disease, lncRNA interleukin 6 antisense RNA 1, S100A9, macrophages, Mendelian randomization

## Abstract

This study investigates the role of interleukin 6 antisense RNA 1 (*IL6‐AS1*), a highly expressed long noncoding RNA (lncRNA), in chronic obstructive pulmonary disease (COPD). An adeno‐associated virus (AAV) was used to induce the expression of *IL6‐AS1* in mice, and they were exposed to cigarette smoke to establish a COPD model. *IL6‐AS1*‐overexpressing mice exposed to cigarette smoke demonstrated exacerbated COPD‐like pathologies. Integrated with single‐cell RNA sequencing analysis of COPD patients and pulmonary fibroblast–macrophage coculture system, our findings indicate that the upregulation of *IL6‐AS1* in fibroblasts enhances the interaction between the S100A9 protein and the AGER and TLR4 receptors on lung macrophages, thereby exacerbating pulmonary inflammation. The molecular mechanism likely involves exosome‐mediated secretion, with *IL6‐AS1* binding to S100A9 protein. These findings suggest that *IL6‐AS1* may facilitate crosstalk between fibroblasts and macrophages, contributing to increased pulmonary inflammation, an effect that can be blocked by paquinimod. Mendelian randomization analysis further suggests a potential shared causal variant between *IL6‐AS1* and COPD risk. Taken together, this investigation provides valuable insights into the function of *IL6‐AS1* and its potential implications for the pathogenesis and therapeutic strategies in COPD.

## Graphical abstract

Clinical cohort analyses in COPD patients demonstrated lncRNA *IL6‐AS1* overexpression inversely correlating with lung function. AAV‐mediated transgenic models and coculture system revealed fibroblast‐derived *IL6‐AS1* stabilizes S100A9 through exosomal transfer to macrophages, promoting TLR4/AGER binding and NF‐κB‐driven inflammatory amplification. Mendelian randomization (MR) analyses confirmed the causal role of *IL6‐AS1* in COPD pathogenesis and lung function decline

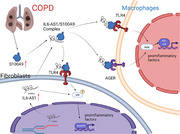

## 1 Introduction

Chronic obstructive pulmonary disease (COPD) is a complex lung disorder characterized by respiratory symptoms such as dyspnea, cough, and sputum, accompanied by a progressive limitation of airflow [1]. These symptoms often arise from abnormalities affecting the airways, including bronchitis and bronchiolitis, as well as the alveoli, particularly emphysema [2]. A combination of environmental and genetic factors has been demonstrated to influence the development of these conditions, with key environmental factors comprising smoking and exposure to airborne pollutants, while the genetic element most closely associated with COPD is the SERPINA1 gene mutation, which leads to a deficiency of α1‐antitrypsin [3, 4, 5]. Pathological changes in COPD encompass emphysema, chronic bronchitis, airway fibrosis, pulmonary hypertension, and alterations in cellular morphology [6].

In the human genome, noncoding sequences constitute approximately 98% of the genome, with nearly 90% of these regions undergoing transcription [7]. Among these transcribed noncoding sequences, those exceeding 200 nucleotides in length are categorized as long noncoding RNAs (lncRNAs) [8], whose principal role is to orchestrate various biological and pathological processes by forming complexes and interacting with proteins, RNA, or DNA [9]. Despite some studies reporting on the mechanisms via which lncRNAs regulate inflammation, comprehensive studies in this area remain limited. Many studies have found significant alterations in the noncoding RNA profiles in the lungs of COPD patients, particularly in lncRNAs, suggesting their potential key role in the progression of COPD [10]. As research deepens, an increasing number of lncRNAs have been reported to be involved in the biological processes of COPD [11]. Our previous sequencing of lung tissue from COPD patients and associated lncRNA investigations revealed that lncRNA *IL6‐AS1* is mainly upregulated in fibroblasts, leading to an increase in IL‐6 expression [12]. Additionally, Smad3‐mediated lncRNA *HSALR1* amplifies the nonclassical TGF‐β1 signaling pathway by binding to HSP90AB1 [13]. Furthermore, lncRNA *COPDA1* increases the expression of MS4A1 and promotes smooth muscle cell proliferation [14]. Although in vivo experiments are currently limited, these lncRNAs may play roles in COPD pathogenesis and their effects in the lungs require further investigation. [15]. Therefore, in this experiment, we conducted a follow‐up in vivo animal experiment on *IL6‐AS1*.

S100A9, a protein derived from myeloid cells, has been found to initiate downstream inflammatory pathways by binding to TLR4 [16]. It also significantly contributes to various inflammatory lung diseases, including COPD and asthma [17]. Recently, it has been demonstrated to be involved in regulating immune cell migration, activation and stimulation of lung fibroblast cells through TLR4‐mediated or RAGE‐mediated signaling pathways [18]. Notably, mice lacking S100a9 (S100a9^−^/^−^) exhibited improved COPD‐like pathological changes following chronic exposure to cigarette smoke (CS), suggesting a potential role for S100A9 in the development and progression of COPD [19]. In this study, we further investigated the in vivo function of *IL6‐AS1*. Molecular biology analysis revealed that *IL6‐AS1* can bind to the S100A9 protein via exosomes to exert inflammatory effects, highlighting the complex role of lncRNAs in tissues, particularly in the lungs.

Mendelian randomization (MR) is a method that employs genetic polymorphisms as instrumental variables to establish causal relationships between genes, including noncoding genes and diseases. By utilizing the random allocation of chromosomal alleles during gamete formation, MR facilitates the inference of causality in epidemiological data while reducing the influence of confounding factors [20]. Many studies have already used MR based on gene expression Quantitative Trait Locis​ (eQTLs) to explore the causal relationships between gene expression and diseases or traits, including COPD and lung function [21]. This approach provides additional evidence supporting the role of genes in disease pathogenesis.

In this study, we hypothesized that *IL6‐AS1* contributes to inflammation in vivo and developed an IL6‐AS1 mouse model by delivering adeno‐associated virus (AAV) through intratracheal instillation and exposing the mice to CS. Our findings from single‐cell sequencing and MR analyses supported the hypothesis, which provides additional evidence of the role and impact of IL6‐AS1.

## 2 Results

### 2.1 lncRNA IL6‐AS1 Expression is Increased in COPD Lungs and is Negatively Correlated with Lung Function in COPD Patients

**FIGURE 1:**
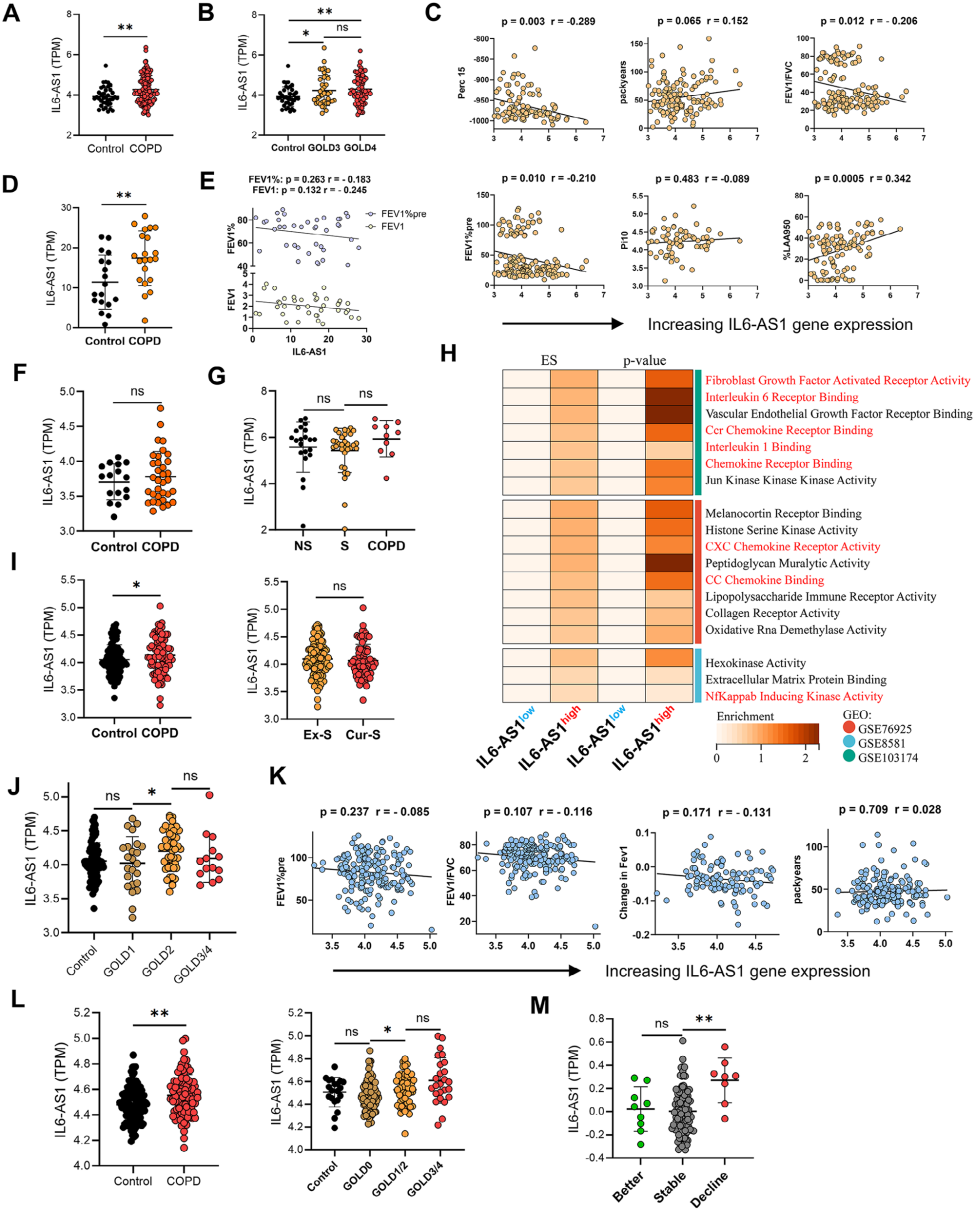
LncRNA *IL6‐AS1* expression is increased in COPD lungs and negatively correlates with lung function in COPD patients. (A and B) Expression of *IL6‐AS1* in the lungs of healthy smokers and COPD patients from the GSE76925 database (*n* = 37 smokers and *n* = 110 COPD patients, including *n* = 40 GOLD3 patients, *n* = 70 GOLD4 patients). (C) Correlation analysis of *IL6‐AS1* expression with various parameters, including FEV_1_% predicted (FEV_1_%pre), FEV_1_/FVC ratio, pack years, Perc15, % low attenuation area at −950 HU (%LAA950), and Pi10, based on GSE76925. (D and E) Expression of *IL6‐AS1* in the lungs of control and COPD patients (D), and the correlation of *IL6‐AS1* with FEV_1_%pred and FEV1 (E) from the GSE8581 database (*n* = 18 non‐COPD patients and *n* = 21 COPD patients). (F and G) Expression of *IL6‐AS1* in the lungs of control and COPD patients (F), including GOLD1 patients and GOLD2 patients (G) from the GSE103174 database (*n* = 16 non‐COPD patients and *n* = 34 COPD patients, including *n* = 7 GOLD1 patients, *n* = 27 GOLD2 patients). (H) Heatmap illustrating the most significantly enriched gene lists in the lungs of populations with high and low *IL6‐AS1* expression, as determined by GSEA of the GO molecular function (MF) set using the GSE76925 dataset. The nominal enrichment score (ES) and *p* value were generated by the GSEA software, which revealed the enrichment score's significance relative to a null distribution. (I and J) *IL6‐AS1* expression was analyzed in bronchial brushings of healthy smokers (*n* = 107) and COPD patients (*n* = 86), as well as in ex‐smokers (*n* = 116) and current smokers (*n* = 79)(I). Additionally, *IL6‐AS1* expression was evaluated among COPD patients classified as GOLD1 (*n* = 24), GOLD2 (*n* = 48), and GOLD3/4 (*n* = 14), using data from GSE37147 (J). (K) Correlation analysis was conducted to investigate the relationship between *IL6‐AS1* expression and clinical parameters, including FEV_1_%pre, FEV_1_/FVC ratio, pack years, and changes in FEV1, utilizing data from GSE37147. (L) *IL6‐AS1* expression in bronchial biopsies was assessed in non‐COPD and COPD patients, as well as across subgroups including healthy smokers (*n* = 20), chronic bronchitis patients (GOLD0, *n* = 94), GOLD1/2 patients (*n* = 58), and GOLD3/4 patients (*n* = 25), using data from GSE162635. (M) Expression of *IL6‐AS1* in transbronchial biopsies was evaluated during follow‐up, with patients categorized into groups based on pulmonary function: improved (better, *n* = 9), stable (stable, *n* = 100), and deteriorated (decline, *n* = 8). Data are presented as mean ± SD. *p* Values in the charts were calculated using two‐tailed Mann–Whitney tests (A, B, D, F, G, I, J, and L) and two‐tailed Pearson correlation tests (C, E, and K).

Initial investigations identified lncRNA *IL6‐AS1* through RNA‐seq analysis of COPD patient cohorts [12, 14]. Then, we conducted in vitro experiments to validate the functional role of *IL6‐AS1*. Validation in vitro prompted further evaluation of its expression in lung tissues (110 COPD patients vs. 37 smoker controls; GSE76925^22^). *IL6‐AS1* was markedly upregulated in GOLD3–4 patients (Figure 1A,B) and inversely correlated with forced expiratory volume in 1 second percent predicted (FEV_1_%pre), FEV_1_/forced vital capacity (FEV_1_/FVC), and percentile 15 of the expiratory flow‐volume curve (Perc15), while positively associating with percentage of low attenuation area below −950 Hounsfield units (%LAA950). No significant associations emerged with pack years or airway wall thickness at an internal perimeter of 10 mm (Pi10; Figure 1C).

Consistent trends were observed in GSE8581[23] (Figure 1D), but with nonsignificant inverse correlations to FEV_1_%pre and FEV_1_/FVC (Figure 1E). Analysis of GSE103174 (16 healthy vs. 37 COPD [GOLD1–2] [24]) corroborated elevated *IL6‐AS1* in COPD (Figure 1F,G). Smoking status showed no impact on *IL6‐AS1* expression (Figure S1A), and COPD‐related elevation trended negatively with lung function parameters (Figure S1B,C).

Using gene set enrichment analysis (GSEA) based on Gene Ontology (GO), we divided patients from above three cohorts (GSE76925, GSE8581, and GSE103174) into groups with high and low *IL6‐AS1* expression levels, and our analysis revealed significant enrichment of differentially expressed genes (DEGs) in pathways related to fibroblast growth factor receptor activity, interleukin/chemokine receptor binding (CCR/CXC), NF‐κB‐inducing kinase activity, and related inflammatory mediators (Figure 1H).

Furthermore, elevated *IL6‐AS1* expression extended to bronchial brushings from COPD patients (GSE37147) [25], mirroring lung tissue trends: higher levels in advanced GOLD stages (Figure 1I,J), negative correlations with FEV_1_%pre and FEV_1_/FVC, and no association with pack years (Figure 1K). In a bronchial biopsy cohort (20 controls, 94 GOLD0, 83 COPD), *IL6‐AS1* expression peaked in GOLD3–4 patients (Figure 1L) and was highest in the clinical decline subgroup (Figure 1M). Collectively, these findings indicate that *IL6‐AS1* is highly expressed in lung tissue and bronchial brush biopsy samples of COPD patients across multiple cohorts, and its expression is negatively associated with lung function and emphysema indices, suggesting that *IL6‐AS1* may have undefined pathogenic functions and a potential role in driving COPD progression.

### 2.2 The AAV‐Constructed *IL6‐AS1* Mice Demonstrated Exacerbated COPD‐Like Pathological Changes

**FIGURE 2:**
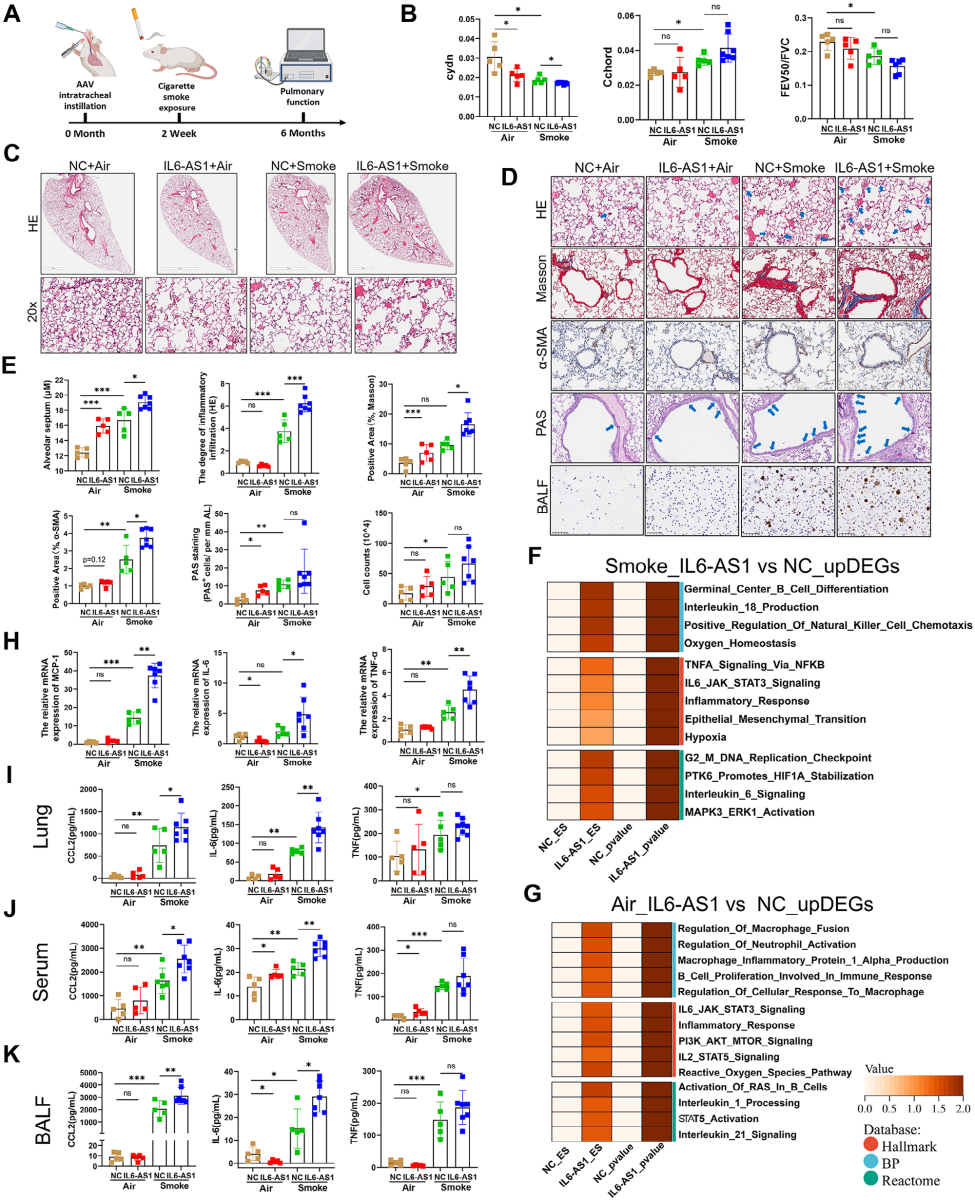
The AAV‐constructed *IL6‐AS1* mice demonstrated exacerbated COPD‐like pathological changes following cigarette smoke exposure. (A) Schematic representation of the establishment of *IL6‐AS1* mice, with lung function assessments and tissue sampling conducted after 6 months of cigarette smoke exposure. (B) Evaluation of lung FEV_50_/FVC, Cchord, and Cydn in four distinct groups of mice. FEV50/FVC represents forced expiratory volume at 50 ms (FEV50) relative to forced vital capacity (FVC); Cydn indicates lung dynamic compliance; Cchord denotes lung compliance. (*n* = 7 for Smoke+ *IL6‐AS1*, *n* = 5 for other three groups). (C–E) Histological sections of lung tissues from the four experimental groups subjected to HE staining (C); histological sections of lung tissues from the four experimental groups assessed through HE stains, Masson staining, IHC staining for α‐SMA, PAS staining, and cell smears for inflammatory cells in BALF (D). The bar chart presents the relevant pathological statistics (E). (*n* = 7 for Smoke+ *IL6‐AS1*, *n* = 5 for other three groups). Data are presented as mean ± SD. *p* Values in charts were determined using one‐way ANOVA Bonferroni's multiple comparisons test (B, C, and D). (F and G) GSEA results for upregulated DEGs in IL6‐AS1 mice compared with wild‐type mice in the Smoke group (G) and air group (F), spanning Hallmark, biological processes (BP), and Reactome pathways. *p* Values are represented as log10 values. (H) qRT‐PCR analysis to assess the expression of inflammation‐associated genes (IL‐6, MCP‐1, TNF‐α) across the four groups of mice. (*n* = 7 for Smoke+ *IL6‐AS1*, *n* = 5 for other three groups). (I–K) Evaluations of the secretion levels of IL‐6, MCP‐1, and TNF‐α within lung tissue homogenates (I), serum (J), and BALF (K) were conducted using a CBA array. (*n* = 7 for Smoke+ *IL6‐AS1*, *n* = 5 for other three groups). Data are presented as mean ± SD. *p* Values in charts were determined by one‐way ANOVA Bonferroni's multiple comparisons test (B, E, H, I, J, and K).

To investigate the in vivo function of *IL6‐AS1*, we engineered an AAV encoding human *IL6‐AS1* (AAV‐*IL6‐AS1*) for intratracheal delivery in C57BL/6 mice, as no murine ortholog exists (Figure S2A). AAV‐transduced mice [validated via Quantitative Reverse Transcription Polymerase Chain Reaction (qRT‐PCR) and Fluorescence In Situ Hybridization (FISH); Figure S2B,C] were exposed to air or CS for 6 months. CS‐exposed AAV‐*IL6‐AS1* mice exhibited significant lung function decline (reduced dynamic compliance, FEV_100_; increased resistance index, chord compliance) compared with air‐exposed controls (Figures 2B and S2D).

*IL6‐AS1* overexpression exacerbated CS‐induced emphysema, airway remodeling, and inflammatory infiltration. However, air‐exposed AAV‐*IL6‐AS1* mice showed no significant differences in inflammatory infiltration, airway remodeling, or related parameters compared with AAV‐NC controls (Figure 2C–E). PAS staining and BALF analysis revealed trends toward goblet cell hyperplasia and altered cellularity in IL6‐AS1 groups, though these changes lacked statistical significance (Figure 2D,E).

RNA‐seq of lung tissues (*n* = 3; Figure S3A,B) revealed *IL6‐AS1*‐driven upregulation of inflammatory pathways (IL‐6/MAPK/PI3K–AKT; Figure 2F,G) and downregulation of cilia function, DNA repair, and metabolism‐related genes in both air‐ and smoke‐exposed groups (Figure S3C,D). Smoke exposure further elevated chemokine/inflammatory factor expression in *IL6‐AS1* mice (Figure S3E), accompanied by NF‐κB and p38 MAPK activation (Figure S3F), which are known to primarily serve as upstream pathways for inflammatory factors.

RNA‐seq identified COPD‐associated inflammation/extracellular matrix (ECM)‐related genes (IL‐6, CCL‐2/7, TNF‐α, CXCL‐15, ICAM‐1, ELN1) as DEGs (Table S1). Experimental validation confirmed these DEGs except MMP12 (Figures 2H and S4A). Given the inflammatory annotation of DEGs, we quantified proinflammatory factors via cytometric bead array (CBA). Our analysis revealed elevated secretion of CCL‐2, TNF‐α, and IL‐6 in AAV‐*IL6‐AS1* mice, although the difference in TNF‐α levels did not reach statistical significance (Figure 2I–K). No differences were observed in CXCL‐9, IL‐10, IFN‐γ, CCL‐5, or CSF‐3 levels (Figure S4B,C). Collectively, these findings indicate that IL6‐AS1 may primarily exert proinflammatory effects in vivo.

### 2.3 *IL* *6* *‐* *AS1* Exhibits Specific Binding to S100A9 in both in Vivo and in Vitro Settings

**FIGURE 3:**
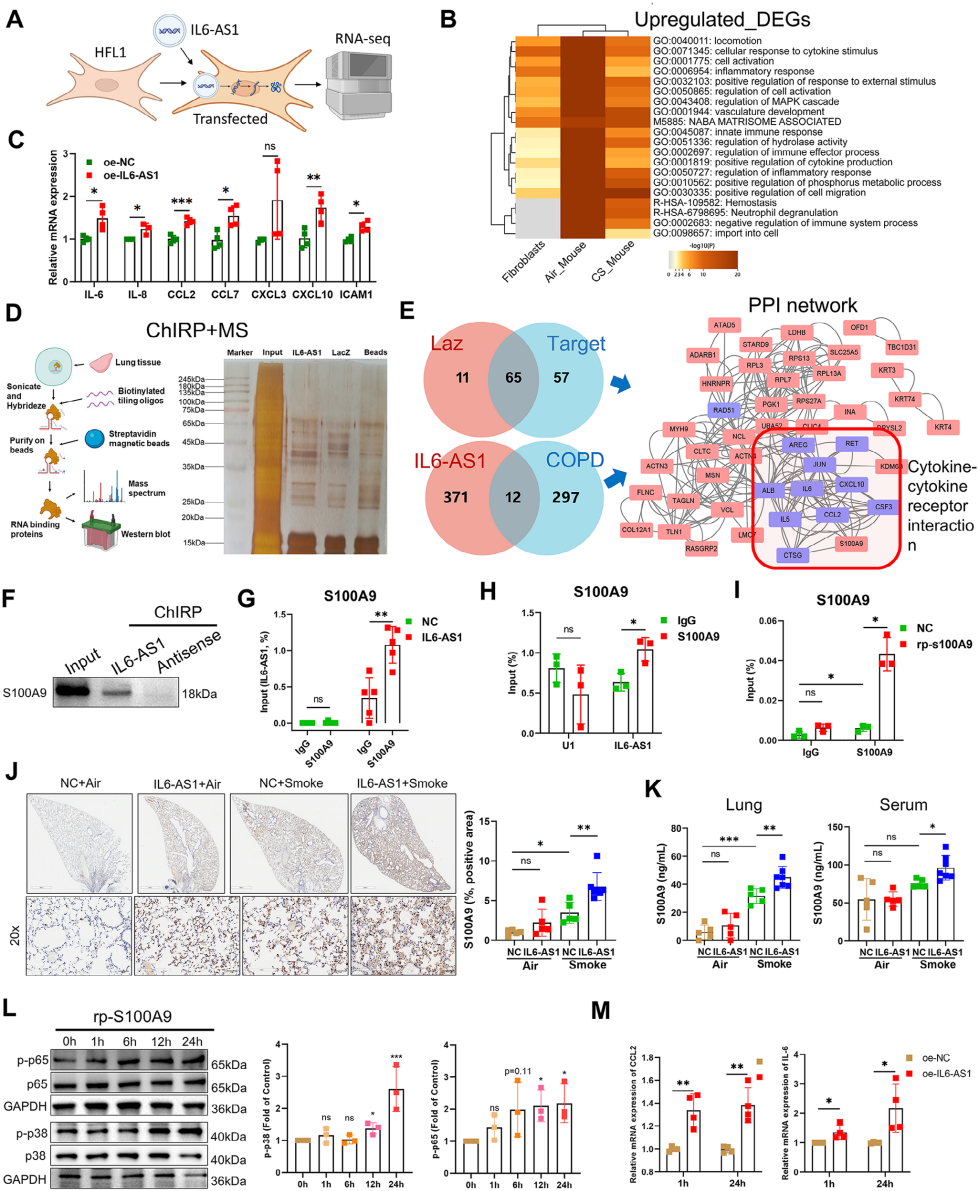
*IL6‐AS1* exhibits specific binding to S100A9 in both in vivo and in vitro settings. (A) Diagrammatic representation illustrates RNA‐seq conducted on HFL1 cells following overexpression of *IL6‐AS1*. (B) Metascape enrichment results for differentially upregulated genes in *IL6‐AS1* mice in the air group, IL6‐AS1 mice in the smoke group, and genes upregulated following *IL6‐AS1* overexpression in HFL cells. (C) qRT‐PCR for the expression of inflammation‐associated genes (IL‐6, IL‐8, ICAM‐1, CCL‐2/7, and CXCL‐3/10) in HFL1 cells after the *IL6‐AS1* overexpression. (*n* = 4 biological replicates). (D) ChIRP assays were conducted using either an unspecific lacZ probe or probes designed for *IL6‐AS1* in human lung tissue, followed by silver staining and mass spectrometric sequencing. (E) A PPI network was constructed by combining the distinct binding proteins identified in the mass spectrometry results of the target group with the differentially expressed COPD‐related genes identified in the RNA‐seq. (F) Western blot analysis of the proteins from the proteomics screen after ChIRP assays shows the specific interaction of *IL6‐AS1* with S100A9 protein in lung homogenates. (G) RIP‐qPCR analysis with anti‐S100A9 antibody in wild‐type (NC) and *IL6‐AS1* mice. Anti‐IgG antibody acted as a negative control (*n* = 5). (H) RNA immunoprecipitation followed by quantitative PCR (RIP‐qPCR) analysis using anti‐S100A9 antibody in HFL1 cells. Anti‐IgG antibody and U1 served as negative controls. (*n* = 3 biological replicates). (I) RIP‐qPCR analysis using anti‐S100A9 antibody in HFL1 cells after stimulation with recombinant S100A9 protein (rp‐S100A9). Anti‐IgG antibody served as a negative control. (*n* = 3 biological replicates). (J) Lung tissue sections from the four groups of mice were subjected to IHC staining for S100A9. (*n* = 7 for Smoke+ IL6‐AS1, *n* = 5 for other three groups). (K) The concentration of S100A9 protein in the lung tissue homogenates and serum of the four groups of mice was determined by ELISA. (*n* = 7 for Smoke+ IL6‐AS1, *n* = 5 for other three groups). (L) Western blot illustration reveals the presence of phosphorylated p65 and p38 following stimulation with rp‐S100A9 for 0, 1, 6, 12, and 24 h (*n* = 3 biological replicates). (M) qRT‐PCR evaluation of the expression of CCL‐2 and IL‐6 in *IL6‐AS1*‐augmented HFL1 cells following stimulation with rp‐S100A9 at the 1 and 24‐h time points. (*n* = 4 biological replicates). Data are presented as mean ± SD. *p* Values in charts were determined by one‐way ANOVA Bonferroni's multiple comparisons test (G, H, and I), one‐way ANOVA Tukey's multiple comparisons test (C, L, and M), and unpaired two‐tailed Student's *t*‐test (J and K).

Our prior work established *IL6‐AS1* as a regulator of IL‐6 in human fibroblasts [12]. To extend this, we overexpressed *IL6‐AS1* in HFL1 fibroblasts (Figures 3A and S5A–C) and performed RNA‐seq, revealing chemokine upregulation and TNF/MAPK pathway activation (Figure S5D,E), mirroring observations in *IL6‐AS1* mice. Metascape analysis of cross‐species DEGs showed upregulated genes were inflammation‐associated, while downregulated genes linked to metabolism, cilia function, and proliferation (Figures 3B and S5F). Key DEGs in *IL6‐AS1* mice were concordantly elevated in HFL1 cells (Figure 3C).

LncRNA typically exerts their regulatory functions by interacting with proteins [26]. Our previous research demonstrated similar roles for other lncRNAs [13], based on which we hypothesize that *IL6‐AS1* could follow a similar pattern. To explore *IL6‐AS1*'s mechanistic role, we performed competitive hybridization internal reference protocol (ChIRP)–mass spectrometry (MS) on human lung tissues (Figures 3D and S6A), identifying 65 *IL6‐AS1*‐interacting proteins (Table S2). Intersection analysis of *IL6‐AS1*‐induced DEGs and established COPD‐related genes identified 12 overlapping candidates (Figure 3E and Table S3). STRING‐based PPI network analysis positioned S100A9—a differentially bound protein—as a central node interacting with these overlapping targets (Figures 3E and S6B,C). Structural analysis revealed moderate overall conservation of S100A9 between humans and mice, while MS‐identified binding fragments exhibited striking sequence conservation (UniProt; Figure S6D). Using PRIdictor, we assessed the binding potential of *IL6‐AS1* to S100A9, which confirmed that *IL6‐AS1* effectively binds to both human and mouse S100A9 (Figures S6E and S7).

ChIRP–WB and RIP assays confirmed *IL6‐AS1*/S100A9 binding in vivo (Figure 3F) and in mouse lung tissues (Figure 3G), with concordant HFL1 validation (Figure 3H). Other CHIRP–MS candidates (RPS27A, TAGLN, PGK1) showed no interaction (Figure S8A). As HFL1 cells lack endogenous S100A9, stimulation with recombinant S100A9 (rp‐S100A9) or CS extract (CSE) enhanced *IL6‐AS1*/S100A9 binding (Figures 3I and S8B). These findings establish S100A9 as a functional *IL6‐AS1* partner.

It has been well documented that lncRNAs can enhance protein stability by binding to the target protein, concurrently inhibiting the degradation of the lncRNA [13]. To assess reciprocal stabilization between *IL6‐AS1* and S100A9, actinomycin D‐chase assays demonstrated rp‐S100A9 pretreatment preserved *IL6‐AS1* stability, without affecting control lncRNA *HSALR1* (Figure S8C). Conversely, cycloheximide treatment in *IL6‐AS1*‐overexpressing HFL1 cells showed delayed S100A9 degradation at 3–6 h, though stability converged by 12 h (Figure S8D), indicating time‐dependent mutual stabilization.

Immunohistochemistry localized S100A9 predominantly to alveolar/airway fibroblasts and immune cells in COPD mice, with elevated expression confirmed in smoke‐exposed models (Figures 3J and S8E), consistent with a previous study [18]. *IL6‐AS1* overexpression further increased S100A9 levels in lung tissues (histological and homogenates) and serum, though undetectable in BALF (Figure 3K). However, S100A9 expression was undetectable in BALF (data not shown). GEPIA analysis revealed positive associations between *IL6‐AS1*/S100A9 and smoke‐induced inflammatory factors, though some correlations lacked statistical significance (Figure S9A,B).

Building on established roles of S100A9 in NF‐κB/p38‐mediated inflammation [27], rp‐S100A9 stimulation in HFL1 cells upregulated CCL‐2, IL‐6, and IL‐8 while suppressing ELN expression (Figure S10A), recapitulating COPD‐associated signatures [28, 29]. Secretion analysis revealed parallel CCL‐2/IL‐6 elevation but stable TNF‐α levels (Figure S10B), likely reflecting fibroblast‐specific secretory profiles. Mechanistically, S100A9 activated p38 and p65 pathways (Figure 3L), consistent with prior inflammatory cascadess [30].

In *IL6‐AS1*‐overexpressing HFL1 cells, rp‐S100A9 stimulation induced time‐dependent upregulation of inflammatory factor mRNAs (1 h/24 h posttreatment; Figures 3M and S11), except IL‐8. These data support a synergistic axis wherein S100A9 activation drives proinflammatory signaling and cytokine production in fibroblasts, with *IL6‐AS1* amplifying these effects to potentiate pulmonary inflammation.

### 2.4 *IL6‐AS1* ^+^ Macrophages are Closely Associated with Inflammatory Effects

**FIGURE 4:**
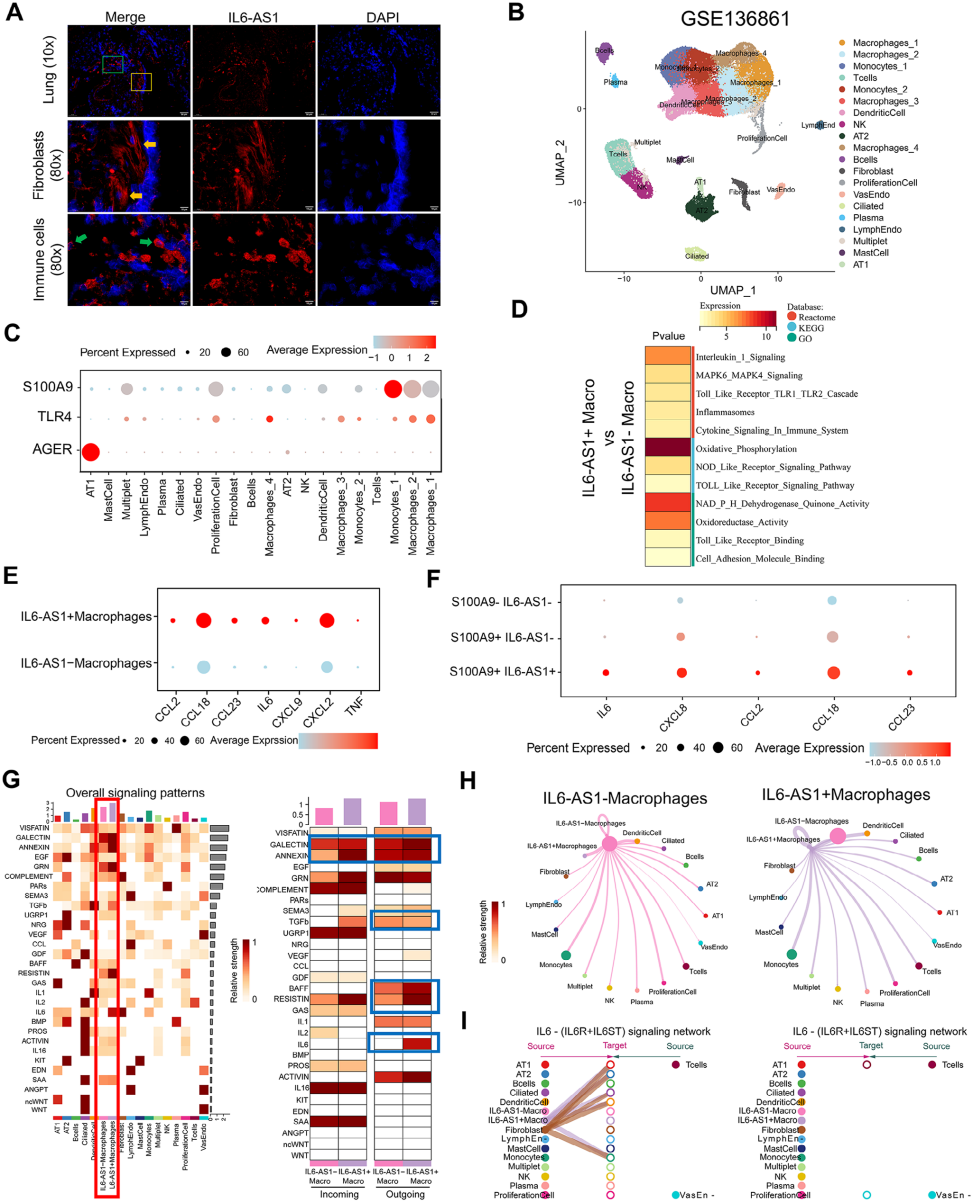
Overexpression of *IL6‐AS1* and stimulation with rp‐S100A9 enhance the inflammatory response in lung fibroblast cells. (A) FISH staining of *IL6‐AS1* in human lung tissue sections. Red represents *IL6‐AS1*, and blue corresponds to DAPI. (B) Umap plot displaying single‐cell transcriptomes of lung tissues from healthy controls and COPD patients from the GSE136861 dataset. (C) Dotplot of the expression of *S100A9*, *AGER* and *TLR4* in different cell types from GSE136861. (D) Enrichment results for Reactome, KEGG, and GO pathways in DEGs between IL6‐AS1 positive and negative macrophages. (E) Bubble chart illustrating the enrichment of related inflammatory genes (including CCL‐2/18 23, IL‐6, CXCL‐2/9, and TNF) in the distribution and expression profiles between *IL6‐AS1* ^+^ and *IL6‐AS1^−^* macrophages, as identified in single‐cell transcriptomes (GSE136861). (F) Bubble chart illustrating the enrichment of related inflammatory genes (including CCL‐2/18/23, IL‐6, and CXCL8) in the distribution and expression profiles between *S100A9^‐^IL6‐AS1^−^*, *S100A9^+^IL6‐AS1^−^*, and *S100A9^+^IL6‐AS1^+^* macrophages from GSE136861. (G) Secretory‐type intercellular interactions among various cell types within *IL6‐AS1^+^* and *IL6‐AS1^−^* macrophage clusters were analyzed, including the degree of enrichment of incoming and outgoing signaling pathways, as identified in single‐cell transcriptomes. Blue frames indicate significantly enriched pathways with distinct differences. (H) Network graphs comparing pulmonary cell interactions in *IL6‐AS1^−^* (left) and *IL6‐AS1^+^* (right) macrophages as ligand cells. Each vertex represents a cellular subpopulation; edges signify ligand–receptor interactions. The thickness of the edges quantifies the cumulative expression of ligand–receptor genes, while the size of each vertex reflects Kleinberg centrality, indicating the cell's role in signaling. Cellular subpopulations are differentiated by color and number. (I) Comparison of IL‐6 (*IL‐6R*+*IL‐6ST*) signaling interactions between *IL6‐AS1* ^+^ and *IL6‐AS1^−^* macrophages and other cell subpopulations.

While S100A9 signals through TLR4/RAGE receptors implicated in COPD pathogenesis [16], these receptors are predominantly expressed on alveolar epithelial cells and macrophages rather than fibroblasts [31]. Single‐cell RNA‐seq of COPD lungs (GSE173896^32^) revealed cell type‐specific inflammatory signatures: fibroblasts (adventitial/alveolar) showed elevated CCL‐2, IL‐6, and ICAM‐1, whereas neutrophils/myofibroblasts/macrophages upregulated CXCL‐3/8/10, TNF‐α, and CCL‐7 (Figure S12A,B). CIBERSORTx deconvolution identified increased resting NK cells, monocytes, and M1 macrophages in *IL6‐AS1* mice (Figure S12C), paralleling human immune infiltration patterns. Notably, *IL6‐AS1* localized to both fibroblasts and morphologically distinct immune cells in human lungs (Figure 4A), suggesting pleiotropic proinflammatory roles across epithelial, stromal, and myeloid lineages.

Due to the low abundance of *IL6‐AS1* and the limited depth of single‐cell sequencing, no *IL6‐AS1* expression was detected in the GSE173896 dataset. Therefore, we utilized an alternative single‐cell dataset (GSE136861) that allowed for the detection of *IL6‐AS1* expression [33] after quality filtering (25 samples: 13 controls/12 COPD). Unsupervised clustering identified 22 cell subtypes (Figures 4B and S13A,B), with IL6‐AS1 predominantly expressed in macrophages (Macrophages_2 cluster), fibroblasts, and endothelial cells, but absent in alveolar epithelium (Figure S13D and Table S4).

Single‐cell profiling revealed S100A9/TLR4 coexpression in monocytes/macrophages, and AGER exclusivity in AT1 cells (Figure 4C). AGER^+^ AT1 cells with elevated *IL6‐AS1* showed heightened inflammatory signatures except TNF (Figure S14A), while S100A9^+^/TLR4^+^ myeloid subsets exhibited progressive inflammation amplification (Figure S14B).

Given that *IL6‐AS1* ^+^ macrophages constituted the numerically dominant subset, subsequent analyses were concentrated on this subpopulation, DEG enrichment analysis identified Toll‐like receptor signaling and inflammatory pathways (Figures 4D and S14C), mirroring murine findings (Figure 3B). These cells overexpressed CCL‐2/18/23, IL‐6, and CXCL‐9/2 (Figure 4E and Table S5).Critically, S100A9^+^/*IL6‐AS1* ^+^ macrophages demonstrated synergistically elevated IL‐6, CXCL‐8, and CCL‐2/18 versus single‐positive or double‐negative subsets (Figure 4F).

Cell‐cell communication analysis revealed broader interaction networks in *IL6‐AS1* ^+^ versus IL6‐AS1^−^ macrophages, with enhanced bidirectional signalings (Figures 4G,H and S14D). Key enriched pathways included GALECTIN (LGALS), TNFSF13B (BAFF), and IL‐6 signaling (Figure 4G). Spatial mapping showed IL‐6 signaling targeted AT1/AT2 cells and monocytes, –TNFSF13C engaged B cells, and LGALS9‐CD44 broadly interacted across cell types (Figures 4I and S14E), all implicated in inflammation [28, 34, 35].

Moreover, we identified *IL6‐AS1* expression in macrophages from BALF in two independent cohorts of COPD patients (GSE13896^36^ and GSE130928^37^; Figure S15A) showed smoking‐dependent downregulation but disease‐associated upregulation (Figure S15B). GSEA revealed high *IL6‐AS1* levels correlated with IL‐6 pathway activation, inflammatory responses, epithelial–mesenchymal transition (Figure S15C), and M1 polarization determined by CIBERSORTx analysis (Figure S15D,E), reinforcing their proinflammatory phenotype.

### 2.5 Exosomes Mediate the Transfer of *IL6‐AS1* from HFL1 Cells to THP‐1 Cells to Facilitate the Activation of the Downstream S100A9–TLR4/AGER Signaling Pathway

Previous studies have demonstrated S100A9's established role in COPD‐associated ECM remodeling [38], we explored *IL6‐AS1*'s macrophage regulation. We assessed *IL6‐AS1* levels in THP‐1 cells, phorbol 12‐myristate 13‐acetate (PMA)‐induced THP‐1 macrophages, and HFL1 cells. *IL6‐AS1* expression was markedly higher in HFL1 fibroblasts versus THP‐1‐derived macrophages (Figure S16A). Given lncRNAs' exosome‐mediated intercellular communication [39], we hypothesized fibroblast‐to‐macrophage *IL6‐AS1* transfer.

TEM confirmed the typical goblet morphology of these exosomes (Figure S16B) with diameters of 80–107 nm (Figure S16C), and expressed exosomal markers CD63/CD81 while lacking Histone 3 (Figure S16D). *IL6‐AS1* was detected within these exosomes (Figure S16E), with CSE exposure significantly enhancing its exosomal enrichment (Figure S16F), demonstrating smoke‐responsive extracellular packaging of IL6‐AS1.

**FIGURE 5:**
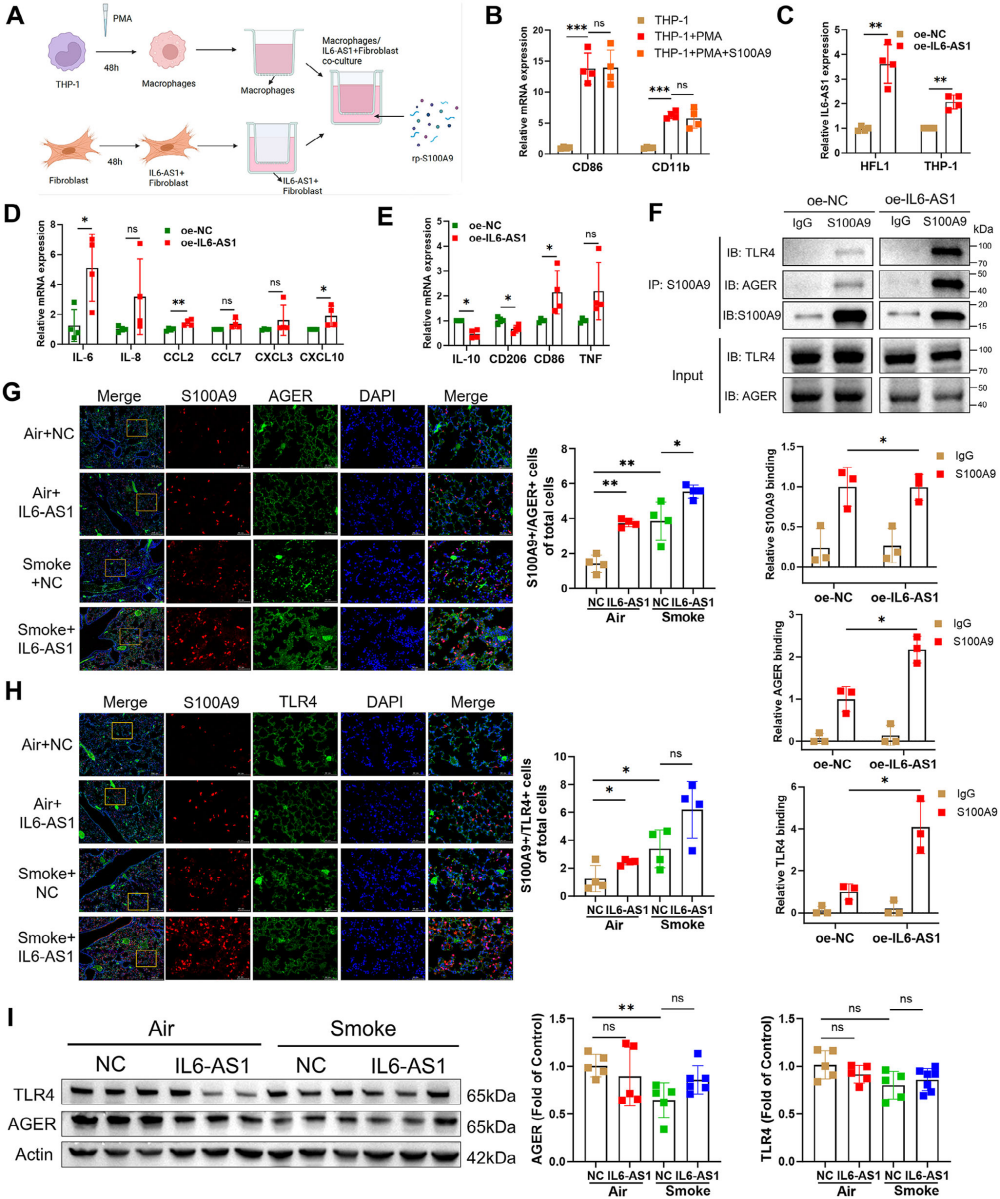
*IL6‐AS1* on fibroblasts can facilitate the binding of S100A9 protein to TLR4 and AGER receptors on macrophages. (A) Schematic representation of the coculture system involving macrophages and fibroblasts. (B) qRT‐PCR assay determining *CD86* and *CD11b* expression in THP‐1 cells following coculture, after stimulation with PMA solely or in conjunction with rp‐S100A9 (*n* = 4 biological replicates). (C) qRT‐PCR assay measuring the expression of *IL6‐AS1* in HFL1 and Thp‐1 cells following coculture. (*n* = 4 biological replicates). (D) qRT‐PCR assay analyzing the expression of inflammation‐associated genes (*IL‐6*, *IL‐8*, *CCL‐2/7*, and *CXCL‐3/10*) in THP‐1 cells after coculture (*n* = 4 biological replicates). (E) qRT‐PCR assay of *IL‐10*, *CD206*, *CD86*, and *TNF‐α* expression in Thp‐1 cells following coculture (*n* = 4 biological replicates). (F) Co‐IP analysis of Thp‐1 cocultured with *IL6‐AS1*‐overexpressed HFL1 using anti‐S100A9 antibody. Western blot was used to verify the co‐IP results with anti‐S100A9, anti‐TLR4, and anti‐AGER antibodies (*n* = 3 biological replicates). (G and H) IF double staining was performed using anti‐S100A9/anti‐TLR4 antibody (G) or anti‐S100A9/anti‐AGER antibody (H) in mouse lung tissue sections extracted from 4 distinct groups of mice. (*n* = 4). (I) Western blot revealing the presence of TLR4 and AGER in four distinct mouse groupings, utilizing Actin as a control reference. (*n* = 5, *n* = 7 [Smoke+*IL6‐AS1*]). Data shown mean ± SD. *p* Values shown in charts are determined by multiple two‐tailed Students’ *t*‐tests (B, C, D, E, and F) and one‐way ANOVA Bonferroni's multiple comparisons test (G and I).

To assess fibroblast‐derived *IL6‐AS1*'s impact on macrophages, we cocultured PMA‐induced THP‐1 macrophages with HFL1 fibroblasts (Figure 5A). Macrophage differentiation (CD86^+^/CD11b^+^ upregulation) was unaffected by rp‐S100A9 (Figures 5B and S16G). Coculture with *IL6‐AS1*‐overexpressing fibroblasts elevated macrophage *IL6‐AS1* levels (Figure 5C), concomitant with upregulated proinflammatory factors (Figure 5D). *IL6‐AS1* overexpression further skewed macrophages toward an M1 phenotype, upregulating M1 markers (CD86/TNF) while suppressing M2 markers (IL‐10/CD206; Figure 5E), indicating fibroblast‐derived *IL6‐AS1* promotes M0‐to‐M1 polarization.

To investigate *IL6‐AS1*'s role in S100A9 receptor engagement, we performed coimmunoprecipitation (co‐IP) in THP‐1/HFL1 cocultures following S100A9 stimulation. *IL6‐AS1* overexpression significantly enhanced S100A9 binding to TLR4/AGER on macrophages (Figure 5F), with no such effect observed in BEAS‐2B epithelial cells (Figure S17A), suggesting that the functional impact of *IL6‐AS1* may be specific to immune cells such as macrophages, rather than epithelial cells. In *IL6‐AS1* mice, immunohistochemistry revealed intensified S100A9–TLR4/AGER colocalization in lung tissues (Figure 5G,H), despite stable TLR4/AGER protein levels (Figure 5I), indicating *IL6‐AS1* potentiates receptor–ligand interaction rather than altering receptor expression.

### 2.6 Coculture of *IL6‐AS1*‐Overexpressing Fibroblasts with Macrophages Aggravates Inflammation

**FIGURE 6:**
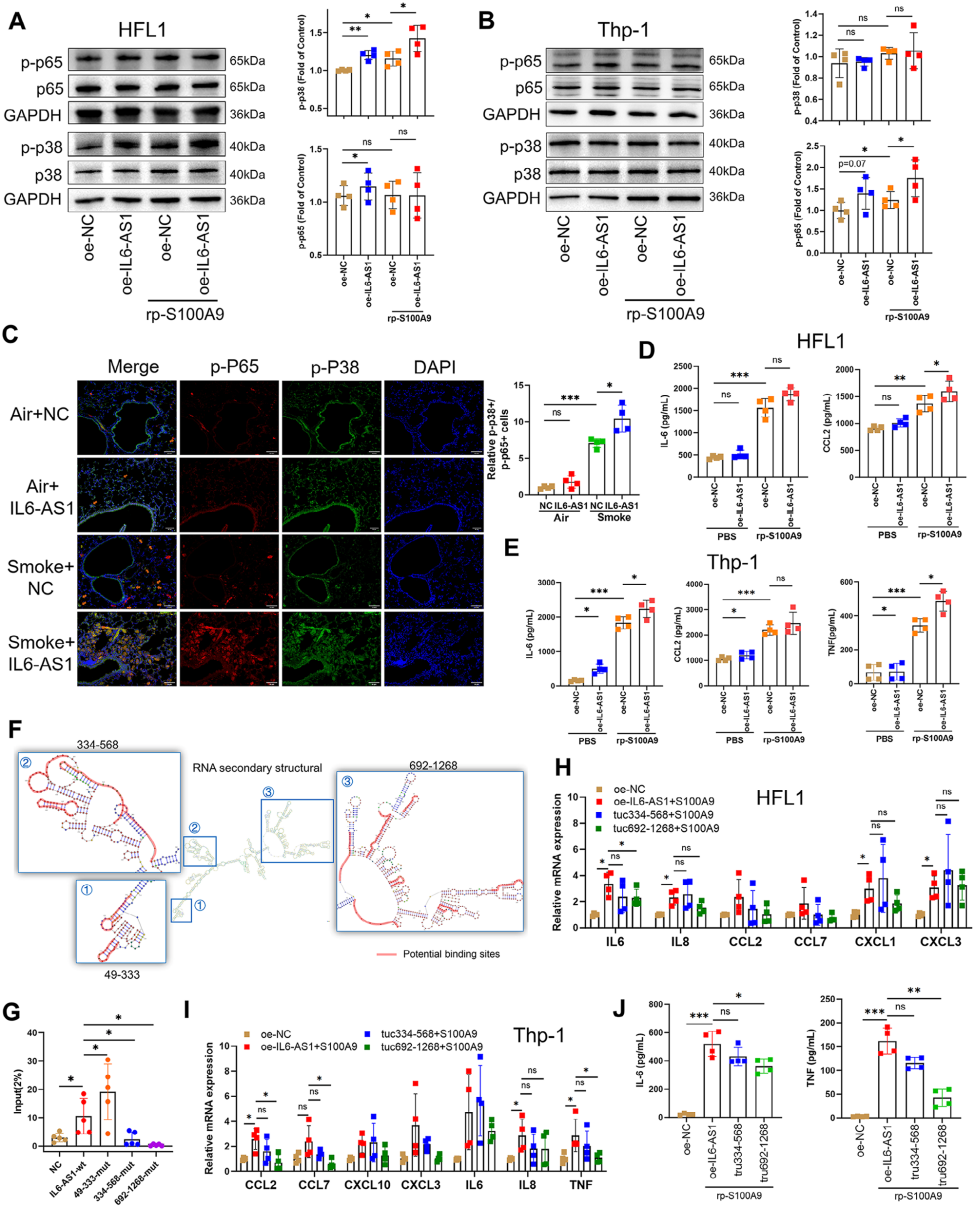
Coculturing *IL6‐AS1* overexpressed fibroblasts with macrophages can enhance the secretion of inflammation‐related factors in both cell types. (A and B) Western blot illustrates the phosphorylation levels of p65 and p38 in HFL1 (A) and THP‐1 (B) after 24 h of cocultivation and stimulation with rp‐S100A9. (*n* = 4 biological replicates). (C) IF assay detection of phos‐p38 and phos‐p65 double‐positive immune cells in the lungs of the four mouse groups. Red: phos‐p65; green: phos‐p38; blue: DAPI. (*n* = 4 in each group). (D and E) ELISA was performed to detect the secretion of CCL2 and IL‐6 in HFL1 cells (D) and CCL2, IL‐6, and TNF‐α in THP‐1 cells (E) following cocultivation and stimulation with rp‐S100A9. (*n* = 4 biological replicates. (F) Schematic delineation provides insight into the RNA secondary structure of *IL6‐AS1* and the prospective binding sites interacting with the S100A9 protein. Red lines indicated potential binding sites. (G) RIP‐qPCR analysis with an anti‐S100A9 antibody after transfection with either wild‐type or truncated *IL6‐AS1* (49–333aa, 334–568aa, 692–1268aa) in HFL1 cells offers evidence of binding dynamics (*n* = 4 biological replicates). (H and I) qRT‐PCR assay of the expression of inflammation‐related genes (*IL‐6*, *IL‐8*, *CCL2*, *CCL7*, *CXCL3*, *CXCL10*, and *TNF*) in HFL1 cells and Thp‐1 cells following coculture, posttransfection with either wild‐type vector or truncation vector of *IL6‐AS1*. (*n* = 4 biological replicates). (J) The secretion of IL‐6 and TNF‐α in THP‐1 cells following coculture after transfection with wily‐type vector or truncation vector of *IL6‐AS1* were detected by ELISA assay. (*n* = 4 biological replicates). Data are presented as mean ± SD. *p* Values in charts were determined by one‐way ANOVA Tukey's multiple comparisons test (A, B, D, E, F, G, and J), one‐way ANOVA Bonferroni's multiple comparisons test (C), and multiple two‐tailed Student's *t*‐test (H and I).

Coculture of *IL6‐AS1*‐overexpressing fibroblasts with macrophages induced cell type‐specific signaling activation: p38 phosphorylation increased in HFL1 fibroblasts, while p65 phosphorylation elevated in THP‐1 macrophages (Figure 6A,B). In smoke‐exposed *IL6‐AS1* mice, immune cells with activated p65/p38 preferentially accumulated near airways (Figure 6C), suggesting fibroblast‐derived *IL6‐AS1* orchestrates immune cell chemotaxis and pathway activation. Cytokine profiling revealed bidirectional inflammatory amplification: *IL6‐AS1* fibroblasts showed elevated IL‐6/CCL‐2 secretion, while cocultured macrophages produced more IL‐6, CCL‐2, and TNF‐α (Figures 6D,E and S17B). Notably, rp‐S100A9 stimulation in coculture systems further amplified IL‐6, CCL‐2/7, and CXCL‐3/10 in fibroblasts (vs. monoculture; Figure S18A,B), with TNF‐α remaining unaffected.

The association of lncRNA with proteins is facilitated by the secondary structure of the RNA molecule, which includes RNA sequences that form various structural motifs such as stem‐loop structures, hairpins, and other configurations, which create binding sites capable of specific interactions with proteins, allowing lncRNAs to form RNA–protein complexes with their protein counterparts. The secondary structure of *IL6‐AS1* was predicted using AnnoLnc2, and potential binding sites of S100A9 were identified within the stem‐loop structures of the *IL6‐AS1* secondary structure according to the results of PRIdictor (Figures 6F and S7). To investigate these interactions further, three distinct truncations of *IL6‐AS1* were generated (Figure S18C). RIP results demonstrated that compared with *IL6‐AS* *1*‐wt, the tuc334‐568aa and tuc694‐1266aa truncations of *IL6‐AS1* significantly reduced S100A9 enrichment, while tuc49‐332aa had no notable impact (Figure 6G). Coculture of macrophages with truncation‐expressing HFL1 cells (tuc694‐1266) markedly attenuated *IL6‐AS1*‐mediated inflammation: THP‐1 macrophages showed reduced CCL2/7/TNF‐α secretion, while HFL1 fibroblasts exhibited decreased IL‐6 production (Figures 6H–J and S18D,E). These results collectively suggest that *IL6‐AS1* can promote the activation of the p65 and p38 signaling pathways in macrophages and enhance the expression and secretion of related inflammatory factors.

### 2.7 Paquinimod Delays the COPD‐Like Changes Induced by Smoke Exposure in *IL6‐AS1* Mice

**FIGURE 7:**
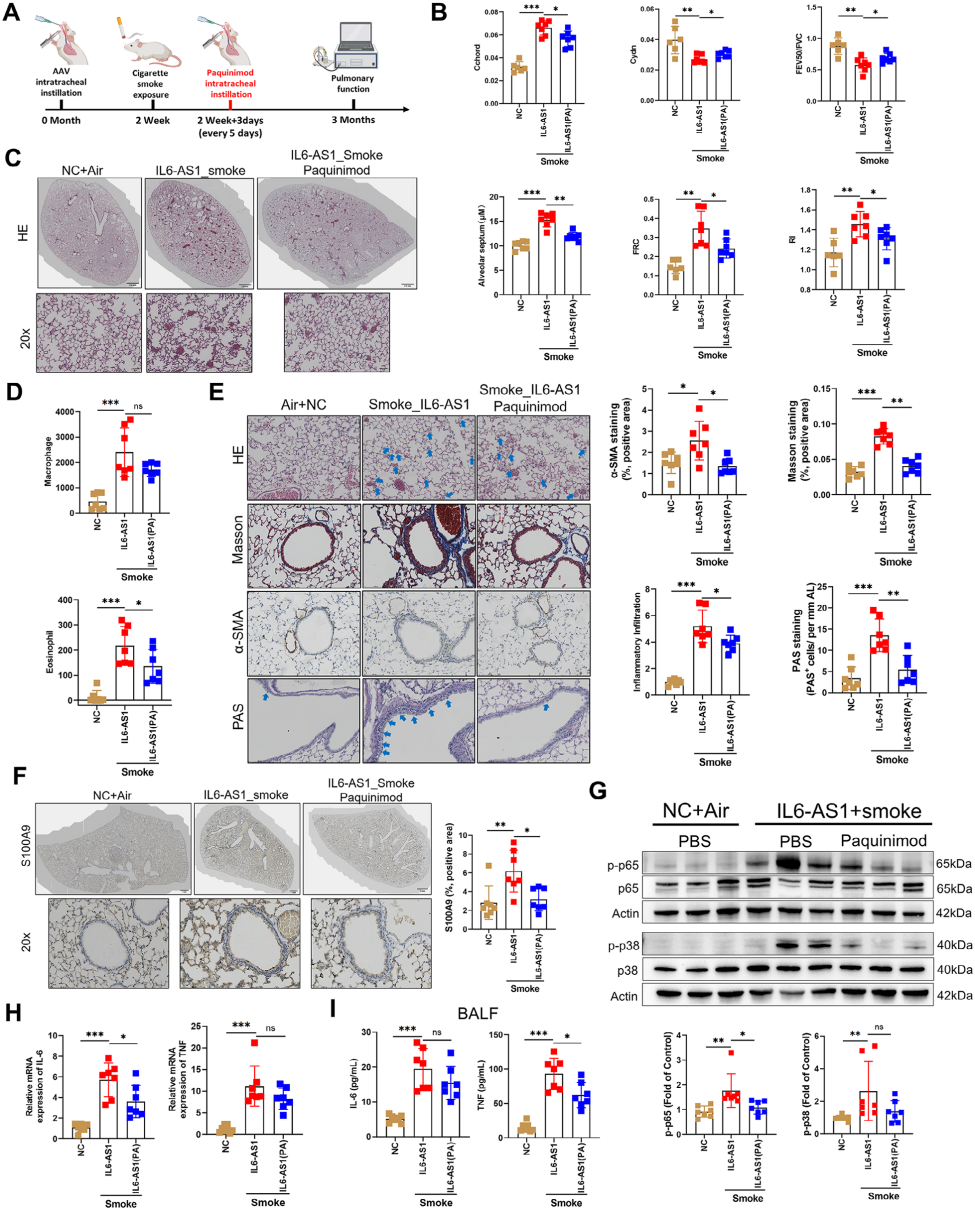
Paquinimod delays the COPD‐like changes induced by smoke exposure in *IL6‐AS1* mice. (A) Schematic illustration delineating the construction of *IL6‐AS1* mice and the administration of paquinimod. (B) Lung FEV_50_/FVC, Cchord, Cydn, FRC, and RI were conducted across the four groups of mice (*n* = 7). (C) Samples of lung tissue from the four distinct groups of mice analyzed using HE staining (*n* = 7). (D) Flow cytometric cell sorting to quantify the populations of macrophages and eosinophils (*n* = 7). (E) Tissue sections from the lungs of the four groups of mice underwent hematoxylin and eosin staining, Masson's trichrome staining, Immunohistochemical staining for α‐SMA, and PAS staining. (*n* = 7). (F) Sections of lung tissues from the four groups of mice were subjected to IHC staining for S100A9. (*n* = 7). (G) Western blot showing the phosphorylation level of p65 and the phosphorylation level of p38 in 4 groups of mice, with Actin as the control. (*n* = 7). (H) qRT‐PCR analysis of IL‐6 and TNF‐α in 3 groups of mice. (*n* = 7). (I) The secretion of IL‐6 and TNF‐α in BALF detected by ELISA. (*n* = 7). Data are presented as mean ± SD. *p* Values in charts were determined by one‐way ANOVA Bonferroni's multiple comparisons test (B, C, D, E, F, G, H, and I).

To dissect *IL6‐AS1*/S100A9 axis in COPD pathogenesis, we administered paquinimod (S100A9 inhibitor blocking RAGE/TLR4 interaction) [18] via intratracheal instillation during CS exposure in IL6‐AS1 mice (Figure 7A). *IL6‐AS1* mice receiving paquinimod showed a significant improvement in lung function, including parameters such as FEV_50_/FVC, Cchord, Cydn, RI and FRC compared with the *IL6‐AS1*+Smoke group (Figure 7B). Flow cytometry of BALF revealed paquinimod‐mediated reduction in eosinophil infiltration and macrophage trends (Figures 7D and S19A). Histologically, paquinimod attenuated smoke‐induced alveolar septal destruction, airway collagen deposition, and mucus hypersecretion (Figure 7C,E).

Paquinimod treatment significantly reduced S100A9 expression (Figure 7F) and suppressed phospho‐p65/p38 levels in *IL6‐AS1* mouse lungs (Figure 7G). This was accompanied by diminished peri‐airway infiltration of phospho‐p38/p65+ immune cells (Figure S19B). Markedly attenuated expression of inflammatory mediators (IL‐6, CCL‐2/7, MMP12) and nonsignificant trends for TNF/IL‐8 reduction were observed (Figures 7H and S20A). Additionally, paquinimod had minimal effects on CXCL‐3, ELN1, and CSF‐3 expression in vivo (Figure S20A).

Paquinimod significantly attenuated TNF‐α and CCL2 levels in lung tissues and BALF, with nonsignificant reductions observed for IL‐6 in BALF/lung tissues and serum TNF‐α (Figures 7I and S20B–D). Serum IL‐6 and CCL2 remained unchanged posttreatment. In HFL1/macrophage cocultures, paquinimod suppressed *IL6‐AS1*‐driven upregulation of IL‐6, CCL7, CXCL1/3 in HFL1 fibroblasts, and IL‐6, IL‐8, CCL‐2/7, and CXCL‐10 in macrophages (Figure S21A,B). While IL‐6/TNF‐α secretion in THP‐1 macrophages and IL‐6 in HFL1 showed nonsignificant reduction trends, CCL‐2 levels remained unaltered in both cell types (Figure S21C,D). These findings collectively suggest that paquinimod may partially inhibit the proinflammatory effects orchestrated by *IL6‐AS1* both in vivo and in vitro.

### 2.8 MR Reveals the Association Between IL6‐AS1 Expression and COPD Risk

**FIGURE 8:**
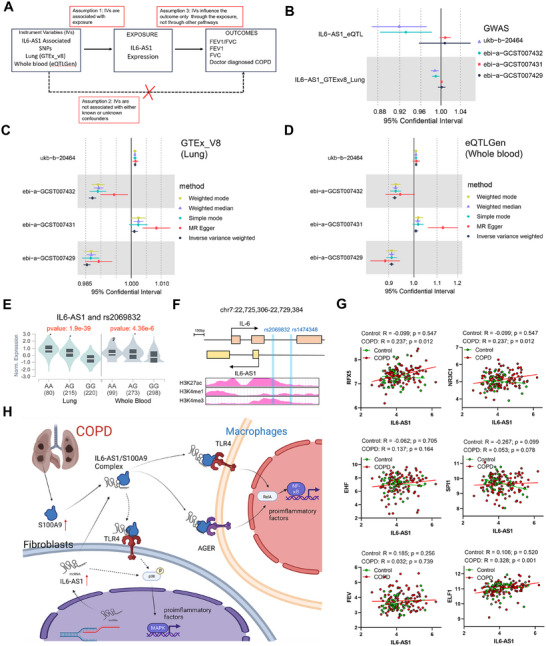
MR analysis indicates that the expression of *IL6‐AS1* in lung and whole blood was associated with COPD progression. (A) Schematic diagram outlines the steps in the MR analysis between *IL6‐AS1* and COPD outcomes (including doctor‐diagnosed COPD, FEV_1_, FVC, and FEV_1_/FVC). The analysis is based on three assumptions: (1) IVs are associated with the exposure, (2) IVs are not influenced by confounders, and (3) IVs affect the outcome exclusively through the exposure, excluding alternative pathways. (B) Forest plot of SMR results of *IL6‐AS1* in lung and whole blood with doctor diagnosis COPD, FEV_1_, FVC and FEV_1_/FVC. (C and D) Forest plots display two‐sample MR analyses of *IL6‐AS1* expression in the lung (C) and whole blood (D) with COPD outcomes after adjusting for the effects of SNPs on other cell types. Effect sizes (beta, 95% CI) are shown as standard deviation changes in COPD outcomes per standard deviation increase in *IL6‐AS1* expression. The points represent effect size estimates, and the whiskers indicate 95% confidence intervals (CIs). (E) Correlation analysis of allelic and replacement bases at rs2069832 with *IL6‐AS1* expression in the lung and whole blood, based on data from the GTEx database. (F) Chromatin locations of rs1474348 and rs2069832, along with histone modification levels (H3K27ac, H3K4me3, and H3K4me1) at the *IL6‐AS1* gene locus, referenced through the UCSC database. (G) Correlation analysis between *IL6‐AS1* and transcription factors ELF1, EHF, FEV, RFX5, SPI1, and NR3C1 in healthy controls and COPD patients, based on the GSE76925 dataset. (H) The schematic representation suggests that fibroblast‐derived *IL6‐AS1*, transported via exosomes, interacts with the S100A9 protein, stabilizing it and promoting its binding to the TLR4/AGER receptor on macrophages. This interaction activates the TLR4/AGER‐mediated NF‐κB pathway, increasing the expression of downstream inflammatory factors in macrophages. Data are presented as mean ± SD. *p* Values in charts were determined by one‐way ANOVA Bonferroni's multiple comparisons test (E) and Pearson correlation two‐tailed (G).

MR analysis integrating *IL6‐AS1*‐associated eQTLs with COPD‐related phenotypes (FEV_1_, FVC, FEV_1_/FVC, and COPD risk; Table S6) revealed tissue‐specific causal associations. Lung tissue analysis (GTEx v8) identified significant SNPs impacting FVC and FEV_1_/FVC, while whole blood analysis (eQTLGen) showed FEV1‐specific effects (Figure 8A,B and Table S7). All significant MR results passed the heterogeneity in dependent instruments (HEIDI) test (*p_HEIDI > 0.05*). Notably, *IL6‐AS1* expression showed nonsignificant positive trends with COPD diagnosis across tissues. Key causal mediators included rs1474348 (lung) and rs2069832 (blood), linking *IL6‐AS1* to impaired pulmonary function.

Two‐sample MR analysis using *IL6‐AS1*‐associated SNPs as IVs (120 lung tissue [GTExv8]; 56 whole blood [eQTLGen]) revealed causal associations with COPD outcomes. For COPD diagnosis, inverse variance weighted (IVW) analysis demonstrated significant positive correlations without heterogeneity or pleiotropy using MR‐PRESSO (Figures 8C and S22A and Tables S8–10).

In lung function analyses, MR‐PRESSO identified pleiotropic outliers in FEV_1_/FVC datasets. After outlier removal, IVW confirmed *IL6‐AS1*'s negative effects on FVC and FEV_1_, but not FEV_1_/FVC. Consistent directionality across methods reinforced result robustness (Figures 8C and S22B–D and Tables S8–10). Peripheral blood MR analysis mirrored lung tissue findings: *IL6‐AS1* showed robust causal links with COPD diagnosis and impaired FEV_1_ via IVW, while FEV1/FVC remained nonsignificant. Crucially, blood‐based FEV_1_ analysis demonstrated no pleiotropy (Figures 8D and S22B and Tables S8–10), bolstering clinical relevance of *IL6‐AS1* in COPD pathogenesis.

COLOC analysis of *IL6‐AS1* eQTLs (lung/blood) with four COPD GWAS datasets (±1 Mb) showed no significant colocalization (PP.H4 < 0.80; Figure S23 and Table S11), indicating distinct causal variants. Notably, rs2069832 (top SMR SNP) emerged as a potential shared locus (Table S12), suggesting genetic convergence between IL6‐AS1 regulation and COPD susceptibility.

COPDGene GWAS analysis revealed rs2069832 and rs1474348 positively correlated with FEV_1_/FVC (Table S13). Both SNPs showed significant associations with *IL6‐AS1* expression in lung/blood in the GTEx database (Figures 8E and S24A) and localized to the *IL6‐AS1* upstream promoter region (≤2 kb), marked by H3K27ac/H3K4me3 enrichment (Figure 8F). These data suggest rs2069832 variants may disrupt transcription factor (e.g., ELF1, RFX5, NR3C1) binding at evolutionarily conserved regions of the *IL6‐AS1* promoter (Figure S24B and Tables S14 and 15). Comparatively, rs1474348 exhibited no transcription factor binding.

Transcriptional network analysis revealed COPD‐specific coexpression patterns: NR3C1 and ELF1 showed disease‐associated positive correlations with *IL6‐AS1* in both lung tissue (Figure S25A) and COPD cohorts (GSE76925; Figure S25B). Subgroup analysis identified enhanced NR3C1/RFX5/ELF1‐*IL6‐AS1* correlations exclusively in COPD patients (Figure 8G), suggesting rs2069832‐mediated transcriptional regulation. Notably, FEV/EHF/SPI1 demonstrated no significant associations, highlighting NR3C1 as a key COPD‐related *IL6‐AS1* regulator.

Integrated MR analyses identified rs2069832 as a causal mediator linking elevated *IL6‐AS1* expression to COPD risk and lung function decline (FEV_1_/FVC reduction). Mechanistically, NR3C1‐mediated transcriptional regulation of the *IL6‐AS1* promoter drives COPD susceptibility through inflammatory pathway activation, positioning NR3C1 as a central epigenetic regulator of *IL6‐AS1*‐driven disease progression

## 3 Discussion

COPD is a complex progressive respiratory condition characterized by persistent inflammation and irreversible airflow restriction [40]. Its primary causes are prolonged exposure to harmful particles and gases, particularly from CS [41], which can lead to chronic inflammation and structural changes in the bronchial passages and lung tissue. Therefore, effectively reducing this persistent inflammation in the respiratory system offers a promising approach to COPD management [29].

In our previous research, we identified *IL6‐AS1* as a highly expressed lncRNA in COPD individuals and explored its role in fibroblasts [12]. To gain a more comprehensive understanding of *IL6‐AS1*’s relevance in COPD, we conducted in vivo functional analysis. In addition, we used AAV to introduce human *IL6‐AS1* expression in mice and then induced a COPD model through exposure to CS to investigate how *IL6‐AS1* influences the interaction between fibroblasts and macrophages and impacts pulmonary inflammation.

To gain a more comprehensive understanding of *IL6‐AS1*’s relevance in COPD, we conducted in vivo functional analysis. In addition, we used AAV to introduce human *IL6‐AS1* expression in mice and then induced a COPD model through exposure to CS to investigate how *IL6‐AS1* influences the interaction between fibroblasts and macrophages and impacts pulmonary inflammation. The AAV‐delivered human *IL6‐AS1* transgene recapitulated fibroblast‐predominant expression patterns observed in human COPD lungs. Despite inherent limitations in AAV tropism, spatial analysis revealed stromal cell‐specific enrichment (particularly airway/alveolar fibroblasts) mirroring human disease distribution. This interspecies conservation implies evolutionarily conserved regulatory mechanisms stabilizing *IL6‐AS1* expression in fibroblasts. Low‐level ectopic expression in epithelial cells reflects residual AAV promiscuity, without compromising overall disease modeling validity.

In our earlier research, we found that *IL6‐AS1* in fibroblasts can bind to the transcription factor EBF1 to promote IL‐6 transcription [12]. However, our current in vivo ChIRP experiments did not detect EBF1 protein, possibly due to its low overall abundance in lung tissues. Nonetheless, we were able to replicate the functional effects of *IL6‐AS1* in fibroblasts, as observed in our previous study, reaffirming our earlier findings. Furthermore, we expanded our investigation to explore the potential functional effects of *IL6‐AS1* throughout the lungs, leading to the discovery of the target protein S100A9. S100A9, commonly known as calprotectin, a calcium‐binding protein primarily produced by neutrophils and circulating macrophages under normal physiological conditions [42]. It plays a protective role in the body by participating in immune responses and maintaining tissue homeostasis [43]. However, in pathological states, particularly chronic inflammatory conditions, S100A9 can become overactive and exhibit proinflammatory properties. Excessive synthesis or release of S100A9 can amplify inflammation and tissue damage [44]. This dysregulated immune response involving S100A9 has been observed in various diseases, including COPD, where it may contribute to sustained inflammation and disease progression [45].

Furthermore, studies have revealed that S100A9 can accumulate and reside within various tissues' ECM, including the lungs. Once embedded in the ECM, S100A9 may interact with other ECM components and immune cells, influencing inflammation and tissue remodeling processes [17]. The precise mechanisms underlying S100A9's function within the ECM and its specific role in pulmonary diseases are subjects of ongoing investigation [46]. In our study, we observed the deposition of S100A9 protein within the ECM of smoke‐exposed mice, consistent with previous reports. Interestingly, the expression of *IL6‐AS1* in the experimental mice appeared to enhance this effect, leading to increased deposition of S100A9 protein in the lungs.

Moreover, we observed an increased infiltration of immune cells, particularly around the airways, in smoke‐exposed *IL6‐AS1* mice, which could be likely mediated by *IL6‐AS1*'s ability to promote the release of chemotactic factors from fibroblasts. Immune cells expressing S100A9 [43], under the regulation of *IL6‐AS1*, showed elevated levels of phosphorylated p65 and p38. These findings suggest that *IL6‐AS1* may modulate a broader range of immune cells beyond macrophages.

The upregulation of *IL6‐AS1* expression appears to play a significant role in the accumulation of S100A9 within the ECM. In our study, FISH and single‐cell RNA sequencing of lung tissues from COPD patients demonstrated that *IL6‐AS1* is expressed in macrophages at levels comparable to those in fibroblasts, highlighting its importance in both cell types. However, *IL6‐AS1* expression was notably lower in PMA‐induced THP‐1 macrophages compared with HFL1 cells, suggesting a potential transfer of *IL6‐AS1* from fibroblasts to macrophages, possibly mediated by extracellular vesicles or exosomes. Further investigation supported this hypothesis, as we found that *IL6‐AS1* may exert its effects on macrophages through exosomal transfer, and coculturing fibroblasts overexpressing *IL6‐AS1* with macrophages led to an increase in *IL6‐AS1* expression within macrophages.

There is increasing evidence suggesting that lncRNAs could be promising targets for disease intervention due to their high specificity and minimal interference with normal biological processes [47]. While S100A9 exhibits context‐dependent pro/anti‐inflammatory duality [43], broad inhibitors like paquinimod face specificity challenges given S100A9's ubiquitous distribution. Targeting *IL6‐AS1* offers precision by disrupting S100A9–AGER/TLR4 interactions and preventing pathological ECM deposition—a strategy requiring empirical validation. Previous studies have demonstrated that S100A9 can increase the expression of the RAGE receptor, thereby activating downstream Erk1/2 and NF‐κB pathways and leading to elevated expression of factors like IL‐6, IL‐8, and IL‐1β. This effect can be inhibited using RAGE‐neutralizing antibodies [48]. In our current study, we found that increased expression of *IL6‐AS1* promotes this effect. However, animal experiments showed that only the *IL6‐AS1* smoke group had a significant difference compared with the air group, while the two air groups showed differences that were not statistically significant. We hypothesize that *IL6‐AS1* may exert its proinflammatory effect in the lungs only when the S100A9 protein increases and infiltrates lung tissue, a process likely triggered by external stimuli or pathological conditions such as COPD. Alternatively, it may be that fibroblasts, in response to external stimuli, release more *IL6‐AS1* in exosome form, thereby enhancing its functional effects.

In this present study, we investigated the impact of *IL6‐AS1* on the binding of S100A9 to TLR4 and AGER receptors in macrophages and bronchial epithelial cells. TLR4 and AGER receptors are widely expressed in various lung cell types, including but not limited to endothelial cells, monocytes, dendritic cells, and alveolar epithelial cells [49, 50]. Therefore, determining whether the effects mediated by *IL6‐AS1* are consistent across different lung cell types would require extensive experimental investigation, which represents one of the limitations of our study. Additionally, it is noteworthy that neither truncating *IL6‐AS1* nor treatment with paquinimod appeared to influence the secretion of CCL‐2 in HFL1 and THP‐1 cells and that *IL6‐AS1* may regulate CCL‐2 through alternative mechanisms, although these mechanisms remain to be elucidated at this point.

While our study successfully induced predominant *IL6‐AS1* expression in murine lung fibroblasts through adenoviral manipulation, mimicking its human tissue localization, and observed resultant effects such as decreased pulmonary function, intensified inflammation, and COPD‐like pathological alterations, it is important to acknowledge that this model has limitations in representing the full functional relevance of *IL6‐AS1* in the human body. The adenoviral infection approach does not fully capture the nuanced spatial and temporal specificity of lncRNA expression within human cells and tissues, nor does it account for potential cis‐regulatory effects that are crucial for their functional consequences [51]. Future in vivo studies of human lncRNAs could benefit from the development of mouse models that incorporate human lncRNAs with cell‐ and chromatin‐specific precision. This approach may involve integrating regulatory elements and targeting sequences into the murine genome to better replicate the native context and functionality of human lncRNAs.

Human cohort analyses revealed COPD‐associated upregulation of *IL6‐AS1* in lung tissues and BALF, showing negative correlation with lung function parameters (FEV_1_/FVC). Our integrated MR analyses first established noncoding RNA causality in respiratory disease, demonstrating *IL6‐AS1*'s negative correlation with FEV_1_/FVC ratio and positive association with COPD diagnosis. Notably, rs2069832 and rs1474348 emerged as key functional SNPs within the *IL6‐AS1* promoter region, potentially mediating transcriptional regulation through TF binding alterations (NR3C1/ELF1).

Despite negative colocalization results, cross‐tissue MR validation confirmed *IL6‐AS1*'s systemic regulatory role, with peripheral blood findings paralleling lung tissue effects. COPD‐specific transcriptional network analyses revealed disease‐associated TF‐*IL6‐AS1* correlations, suggesting SNP‐mediated regulatory rewiring during pathogenesis. These findings position *IL6‐AS1* as a multitissue modulator of COPD progression, with GWAS‐validated SNPs serving as potential severity biomarkers. The conserved *IL6‐AS1*/S100A9–TLR4/RAGE axis across species highlights its clinical translatability, though further validation is required for diagnostic application.

Another key innovation of this study is the use of single‐cell sequencing to investigate lncRNAs, specifically in the context of COPD in human patients. Through single‐cell sequencing of lung tissue from COPD patients, we found that *IL6‐AS1* is predominantly expressed in lung macrophages. However, the scarcity of fibroblasts in the samples resulted in too few *IL6‐AS1*‐positive fibroblasts for meaningful analysis. A notable limitation of our study is the depth of single‐cell sequencing, which may not fully capture the expression levels of lncRNAs like *IL6‐AS1* in lung tissue cells. The observed number of cells expressing lncRNAs may underestimate their actual expression levels. Future studies using advanced high‐throughput single‐cell sequencing techniques could overcome these limitations and offer more comprehensive insights into the role of lncRNAs in lung tissue.

In summary, this study reports the significance of *IL6‐AS1* in the pathogenesis of COPD, as demonstrated by findings from patient cohorts, GWAS analyses, and experimental models. We identified fibroblast‐derived *IL6‐AS1* as an important regulator that, through exosome‐mediated transfer, facilitates the binding of S100A9 to TLR4 and AGER receptors on macrophages. This interaction amplifies inflammatory responses in fibroblasts and macrophages, which are key contributors to the pathological features of COPD (Figure 8H). The exacerbation of COPD‐like symptoms in *IL6‐AS1*‐overexpressing murine models provides further evidence of its in vivo relevance, consistent with in vitro observations. Treatment with paquinimod, a targeted inhibitor of S100A9 and TLR4/AGER, effectively reduced inflammation in these models, suggesting a potential therapeutic avenue. Moreover, single‐cell sequencing of COPD patient lung tissues and MR analyses linking *IL6‐AS1*‐related eQTLs to COPD outcomes corroborated these findings. Despite these advances, further studies are required to establish *IL6‐AS1* as a reliable diagnostic marker or prognostic indicator for COPD. These results position *IL6‐AS1* as a promising molecular target for therapeutic intervention in COPD.

## 4 Material and Methods

### 4.1 Animals and COPD Modeling

All female C57/BL6 mice were obtained from Gempharmatech (Jiangsu), aged 7 weeks and weighing between 22 and 25 g. AAV 9 encoding *IL6‐AS1* (AAV‐*IL6‐AS1*) and the empty vector were sourced from PackGene Biotech (Guangzhou). The mice received an intratracheal instillation of AAV at a dose of 2 × 10^11^ genomic copies while under anesthesia. Following a 2‐week recovery period, mice were used to develop a COPD model. The chronic CS‐induced COPD model was established as previously described. Mice were exposed to CS for 2 h both in the morning and afternoon, 6 days a week, over a period of 6 months. After this exposure, mice were harvested for subsequent analyses. Ethical approval for the animal study was obtained from The First Affiliated Hospital of Guangzhou Medical University (Reference number: SYXK 2020‐0227).

### 4.2 ChIRP Assay and MS

The ChIRP assay and MS were performed with minor modifications to established protocols [12]. ChIRP probes targeting LacZ control and IL6‐AS1 were designed and synthesized by GZSCBIO (Guangzhou). Cells were lysed using a lysis buffer and sonicated on ice with a Cole‐Parmer 130 instrument (USA) in 10‐s bursts followed by 15‐s pauses, for a total duration of 20 min. The sonicated lysate was incubated overnight at 4°C with a biotinylated DNA probe mixture specific for IL6‐AS1 to facilitate hybridization. Binding complexes were collected using magnetic beads that were conjugated with streptavidin, and the proteins were then released with an elution buffer containing Proteinase K at 0.2 U/µL.

### 4.3 Cell Culture and coculture

Human lung fibroblast 1 (HFL1) cells were sourced from ATCC (CCL‐153; Manassas, USA), which was cultured in F‐12K medium (Kaighn's modification of Ham's F‐12) supplemented with 10% FBS (Gibco) and 1% P/S (Invitrogen). BEAS‐2B (CRL‐9609) and A549 (CCL‐185) cells were cultured in RPMI‐1640 medium (30‐2001) with the same condition. THP‐1 cells obtained from Procell (Wuhan, China) were maintained in 1640 medium containing 0.05 mM β‐mercaptoethanol, 10% FBS, and 1% P/S. To differentiate THP‐1 cells into M0 macrophages, they were treated with 100 ng/mL PMA as previously described [52]. In the coculture setup, HFL1 cells were placed in the lower chamber of a transwell for transfection, while THP‐1 cells were added to the upper chamber to facilitate differentiation and adherence. After 48 h of transfection and 72 h of differentiation, THP‐1 cells were cocultured with HFL1 cells, and recombinant S100A9 (rp‐S100A9) was introduced into the lower chamber culture. All primary cells and cell lines were incubated at 37°C in a humidified atmosphere with 5% CO_2_.

### 4.4 Statistical Analysis

Statistical analyses were performed using GraphPad Prism v8.0.1 and SPSS v27.0. Significance between means was assessed using a two‐tailed paired Student's *t*‐test or a one‐way analysis of variance (ANOVA) test. The significance levels were represented as follows: ns (not significant) for *p* ≥ 0.05, *p < 0.05, **p < 0.01, and ***p < 0.001.

## Author Contributions

Erkang Yi, Pixin Ran, and Bing Li conceived and designed the experiments. Erkang Yi, Xiaoyu Wang, Yu Liu, Xinyue Mei, Ge Bai, Weitao Cao, Zihui Wang, Huahua Xu, Qingyang Li, Fan Wu, Jieda Cui, Qingyang Li, Haiqing Li, and Xinru Ran performed the experiments. Erkang Yi and Chegnshu Xie performed bioinformatics analysis. Erkang Yi, Zihui Wang, Fan Wu, and Zhishan Deng performed clinical correlation analysis. Erkang Yi and Xiaoyu Wang wrote the manuscript and analyzed the data. Erkang Yi, Qingyang Li, Yumin Zhou, Wei Hong, Ruiting Sun and Pixin Ran supervised the research and acquired the funding. Erkang Yi, Xiaoyu Wang, Yu Liu, and Zihui Wang contributed equally to this work. All authors have read and approved the final manuscript.

## Ethics Statement

Ethical approval for the animal experiment was obtained by The First Affiliated Hospital of Guangzhou Medical University (Reference number: SYXK 2020‐0227). This article does not contain any studies with human subjects.

## Conflicts of Interest

The authors declare no conflicts of interest or personal relationships that could have appeared to influence the work reported in this paper.

## Supporting information



Supporting Information

## Data Availability

All transcriptome sequencing and MS identification data referenced in our manuscript are available in the CNCB‐NGDC database, with the corresponding OMIX IDs: OMIX008536, OMIX008537, and OMIX008538. Additional publicly available sequencing data were obtained from the GEO database.
