# Supplementary material for: Long Noncoding RNA Interleukin 6 Antisense RNA 1 Promotes Inflammatory Effects in Lung Macrophages via Exosomes Through the S100A9/TLR4 Pathway in Chronic Obstructive Pulmonary Disease Progression
Source: MedComm (2020). 2025 Jun 6;6(6):e70204. doi: 10.1002/mco2.70204 (PMC12141923; doi:10.1002/mco2.70204)
Supplement: Supplementary file 1 — Supporting Information [file MCO2-6-e70204-s001.docx]

Title

Long non-coding RNA interleukin 6 antisense RNA 1 promotes inflammatory effects in lung macrophages via exosomes through the S100A9/TLR4 pathway in chronic obstructive pulmonary disease progression

**Authors**

Erkang Yi^1,2#^, Xiaoyu Wang^1#^, Yu Liu^1#^, Zihui Wang^1#^, Ge Bai^1^, Xinyue Mei^1^, Fan Wu^1^, Chengshu Xie^2^, QiYang Li^1^, Weitao Cao^1^, Huahua Xu^1^, Xinyuan Liu^1^, Jieda Cui^1^, Haiqing Li^1^, Ruiting Sun^1^, Xinru Ran^1,3^, Wei Hong^3^, Zhishan Deng^1^, Bing Li^3^, Yumin Zhou^1, 2^, and Pixin Ran^1, 2*^

**Affiliations**

^1^State Key Laboratory of Respiratory Disease, National Clinical Research Center for Respiratory Disease, Guangzhou Institute of Respiratory Health, The First Affiliated Hospital of Guangzhou Medical University, Guangzhou Medical University, No.195 DongFengXi Road, Guangzhou,510182, Guangdong, China.

^2^Guangzhou National Laboratory. No.9 XingDaoHuanBei Road, Guangzhou International BioIsland, Guangzhou 510005, Guangdong, China.

^3^GMU-GIBH Joint School of Life Sciences, Guangzhou Medical University, Guangzhou, Guangdong, China.

^#^ Correspondence.

Email address: Pixin Ran (pxran@gzhmu.edu.cn). State Key Laboratory of Respiratory Diseases, National Clinical Research Center for Respiratory Diseases, the First Affiliated Hospital of Guangzhou Medical University, 195 Dongfeng Xi Road, Guangzhou, Guangdong 510182, China.

**Supplemental Material and Methods**

**Lung function**

Lung function in mice was assessed using a specialized invasive pulmonary function system, as previously described[1]. After calibrating the system, mice were anesthetized with an intraperitoneal injection of sodium pentobarbital at a dosage of 50 mg/kg, followed by a tracheostomy. The mice were then placed in the DSI Buxco Pulmonary Function Test (PFT) apparatus (Buxco Research Systems, USA) for further evaluation. A range of measurements were taken, including lung chord compliance (Cchord), dynamic compliance (Cdyn), functional residual capacity (FRC), airway resistance (RI), and forced expiratory volume at 50 milliseconds (FEV_50)_.

**Co-immunoprecipitation (Co-IP)**

The Co-IP assay was performed using the Pierce Classic Magnetic IP/Co-IP Kit (Thermo Fischer Scientific, 88804) in accordance with the manufacturer's instructions, utilizing antibodies against IgG (Millipore Sigma 17-700) and S100A9 (73425S, CST). THP-1 and BEAS-2B cells transfected with the IL6-AS1 vector were collected and lysed in ice-cold lysis buffer. The cell lysates were mixed with Co-IP buffer and magnetic beads linked to either the target antibody or anti-IgG, then incubated overnight at 4°C. Following the washing of the beads, the protein-DNA complexes were eluted, and a Western blot analysis was performed to identify the denatured proteins.

**RNA extraction, cDNA synthesis, and qRT-qPCR**

Total RNA was extracted from lung tissues and cells using Trizol reagent (Invitrogen) according to established protocols. cDNA synthesis was performed with the Evo M-MLV RT Kit with gDNA Clean for qPCR (AG), utilizing 1,000 ng of total RNA as recommended by the manufacturer. qRT-PCR was subsequently carried out using the SYBR^®^ Green Premix Pro Taq HS qPCR Kit (AG) and analyzed on a CFX Connect real-time PCR detection system (Bio-Rad, USA). The expression levels of target genes relative to GAPDH, utilized as the internal control, were determined using the 2^-∆∆CT^ method. A comprehensive list of specific primers used in this study is provided in Supplementary Table 16.

**Gene overexpression**

Lentivirus overexpression vectors of IL6-AS1 (oe-IL6-AS1) and control (oe-NC) were obtained from Shanghai Ji Kai Gene Technology Co., Ltd. For the subsequent *in vitro* experiments, HFL1 cells were transfected with the overexpressing vector encoding IL6-AS1 using Lipofectamine 3000 (L3000015, Thermo, USA) following the manufacturer's instructions.

**RNA fluorescence *in situ* hybridization (FISH) and IF assay**

FISH probes specific to IL6-AS1 were obtained from RiboBio Technology Co., Ltd. To assess the cellular localization of IL6-AS1, we conducted the FISH assay following the manufacturer's protocol using the RiboBio FISH kit. The relative fluorescence of cells in each group was visualized with a Leica DM6 M microscope.

The immunofluorescence (IF) assay was performed according to previously described methods. Lung tissue sections were incubated with specific antibodies, including p-p65 (ab76302, Abcam), Phospho-p38 MAPK (#4511S, CST), S100A9 Polyclonal antibody (1:200, 26992-1-AP, Proteintech), TLR4 (1:300, 19811-1-AP, Proteintech), and AGER (1:300, 16346-1-AP, Proteintech) for 2 hours at 37°C. Following this, sections were treated with secondary antibodies: Alexa Fluor 488 goat anti-rabbit IgG (H+L) (1:500, Invitrogen) and Alexa Fluor 594 rabbit anti-mouse IgG (H+L) (1:500, Invitrogen) for 40 minutes. Images were captured using the Leica DM6 M microscope.

**Western blot (WB)**

Western blot analysis was conducted using established protocols[2]. Briefly, cells or lung tissues were lysed in RIPA lysis buffer (89901, Thermo, USA) supplemented with a protease inhibitor cocktail (78430, Thermo, USA) at 4°C for 20 minutes. Protein extracts were then separated by 10% SDS-polyacrylamide gel electrophoresis and transferred to polyvinylidene difluoride (PVDF) membranes (Bio-Rad, USA). After blocking, the membranes were incubated overnight at 4°C with antibodies against S100A9 (26992-1-AP, Proteintech), p-p65 (ab76302, Abcam, UK), p65 (ab32536, Abcam), p38 MAPK (#8690S, CST), Phospho-p38 MAPK (#4511S, CST), Actin (23660-1-AP, Proteintech), and GAPDH (10494-1-AP, Proteintech). Following washing, membranes were incubated with a peroxidase-conjugated secondary antibody (Proteintech). Protein bands were visualized using chemiluminescence on an Amersham Imager 680 (Thermo Fisher Scientific).

**Hematoxylin-Eosin (HE), Masson, Periodic Acid-Schiff stain (PAS) and Immunohistochemistry (IHC) staining**

HE, Masson, PAS, and IHC staining techniques were conducted as previously described[1]. In brief, tissue samples were embedded in paraffin, deparaffinized, and rehydrated before undergoing HE, PAS, and Masson staining. For immunohistochemistry (IHC), lung tissue sections were incubated with antibodies against mouse α-SMA (19245S, CST) and S100A9 (73425S, CST), followed by incubation with Biotin-conjugated Affinipure Goat Anti-Rabbit IgG (H+L) (SA00004-2, Proteintech). Stained sections were visualized using a Digital Pathology Scanner (Aperio CS2).

The mean linear intercept (MLI) was determined to estimate the average diameter of an individual alveolus, using the formula: MLI = total length / number of alveolar septa. To evaluate airway wall thickness, the segmental airway wall area percentage was calculated, defined by the equation: Segmental WA% = [outer bronchus area - airway luminal area] / outer bronchus area. Airway area and the Masson-positive area were measured with ImageJ software, enabling the calculation of the percentage of stained area in relation to the total airway wall area (collagen area / airway area). The same statistical methods were employed to analyze the results of immunohistochemistry for α-SMA (α-SMA area / airway area) and S100A9 (S100A9 area / airway area).

**ELISA assay and Cytometric bead array (CBA)**

Mouse IL-6 (EK206/3-96, Multi Sciences, China), MCP-1 (CCL-2, EK287/2-96, Multi Sciences) and TNF-α (EK182-96, Multi Sciences) ELISA kits were utilized to assess the expression of IL-6, TNF-α and CCL-2 in BALF, serum, lung tissue homogenates or cell supernatant, following the manufacturer's instructions. Human IL-6 (88-7066-88, ThermoFisher), CCL-2 (88-7399-88, ThermoFisher) and TNF-α (88-7346-88, ThermoFisher) were used to measure the secretion of IL-6, TNF-α and CCL-2 in cell culture supernatants from HFL1 and THP-1 cells.

Customized CBA kits were obtained from BD Biosciences and utilized to measure the levels of IL-6, CCL2 (MCP-1), TNF-α, CXCL-9, CSF-3, IL-10, IFN-γ, and CCL-5 following the manufacturer's instructions. The signal intensities of analytes were calculated with reference to the respective standards, and the absolute concentrations of individual analytes were determined using BD FCAP Array Software (BD Biosciences, USA).

The lung BALF, serum and lung tissue homogenates were prepared as previously described. Following lung function tests, BALF and whole blood were promptly collected from the mice. Their lungs were lavaged with two 0.5-ml instillations of ice-cold PBS, and serum was obtained by centrifuging whole blood at 2,000 g for 10 minutes at -4°C. The smallest lung lobe was excised and homogenized in 0.3 ml of ice-cold PBS. In the case of cell culture supernatant, it was collected by centrifugation at 2000×g for 10-15 minutes at room temperature. Murine serum and lung homogenates were diluted at a ratio of 1:9, while murine BALF and cell supernatant were not diluted.

**RNA immunoprecipitation (RIP)**

The RIP assay was performed as previously described protocol[3]. In summary, we utilized the Magna RIP RNA-Binding Protein Immunoprecipitation Kit (Millipore Sigma 17-700) for the procedure. Immunoprecipitation was carried out using antibodies against IgG (Millipore Sigma 17-700) and S100A9 (73425S, CST), according to the manufacturer’s instructions.

**RNA-seq** **analysis and Bioinformatics**

RNA samples were sequenced at BGI (China) using the BGISEQ-500 system, and the resulting RNA-seq data were aligned with the Ensembl v102 transcript annotations. To identify differentially expressed genes (DEGs), we employed the "Limma" package in R software. The differentially expressed mRNAs underwent Gene Ontology (GO) functional enrichment analysis via the Gene Ontology website (http://geneontology.org/). Additionally, Kyoto Encyclopedia of Genes and Genomes (KEGG) pathway analysis was conducted at http://www.kegg.jp/, while Reactome pathway analysis was performed using https://reactome.org/.Protein-protein interaction (PPI) networks for the relevant genes were constructed using data from the STRING database (http://string-db.org) and visualized with Cytoscape (v.3.6.1). For immune infiltration analysis of the sequencing results, we utilized the CIBERSORTx database (https://cibersortx.stanford.edu/). Gene correlations within lung tissues were assessed using the GEPIA database (http://gepia2.cancer-pku.cn/)[4].

Binding sites and scores for transcription factors (TFs) and upstream promoters were predicted using the JASPAR database[5]. The assessment of H3K27ac, H3K4me1, and H3K4me3 levels in chromatin was conducted using the ENCODE database, and SNP locations were identified through the UCSC Genome Browser ([https://genome.ucsc.edu/](https://genome.ucsc.edu/))[6]. TFs associated with SNPs were predicted using EnhancerDB[7] (http://lcbb.swjtu.edu.cn/EnhancerDB/). Lastly, SNP-to-phenotype correlations were analyzed from the COPDgene database[8] (http://www.copdgene.org/).

**Exosome extraction and characterization**

Exosomes were isolated from the conditioned media of HFL1 cells using the Exosome Isolation Reagent (C10130, Ribobio, China) according to the manufacturer’s protocol. The morphology of the isolated exosomes was analyzed using transmission electron microscopy (TEM), as previously described[9]. Nanoparticle Tracking Analysis (NTA; NS300, Malvern Panalytical, Germany) was performed to determine the size and concentration of the exosomes. The isolated exosomes underwent further characterization via qRT-PCR and Western blotting, using GAPDH as internal control. Extracellular vesicle-associated protein markers CD81 and CD63 were detected, while the absence of the exosome-negative protein H3 confirmed the purity of the exosome preparation.

**scRNA-seq analysis**

The single-cell RNA-seq data from COPD lung tissue was obtained from the GEO database (GSE173896[10]), and marker genes for each cell type had been previously described. Another set of single-cell RNA-seq data from COPD lung tissue was downloaded from the GEO database (GSE136861[11]). We reprocessed this data through cleaning and unsupervised clustering, focusing on samples from 13 healthy controls and 12 COPD patients. This reprocessing resulted in the identification of 22 distinct cell subgroups, including Macrophages_1-4, Monocytes_1-2, T cells, Dendritic Cells (DCs), Natural Killer (NK) cells, AT1, AT2, B cells, Fibroblasts, Proliferation cells, Vasendothelial cells, Ciliated cells, Plasma cells, Lymphendothelial cells, Multiplet cells, and Mast cells. The marker genes associated with these subgroups are detailed in the supplementary table.

Our analysis of the single-cell RNA-seq data included normalization, dimensionality reduction, and clustering, all conducted using R and the Seurat package (https://satijalab.org/seurat/). Intercellular communication was analyzed using the 'CellChat' R package (version 1.1.3)[12]. Finally, the results of the single-cell analysis were visualized using the 'ggplot2' R package.

**GEO Database Analysis**

Microarray datasets GSE47460[13], GSE76925[14], GSE103174[15], GSE8581[16], GSE13896[17], GSE37147[18], GSE130928[19], and GSE162635[20] were retrieved from the GEO database. The platforms used for each dataset were: GSE47460 on GPL14550, GSE76925 on GPL10558, GSE103174 on GPL13667, GSE8581 on GPL11153, GSE13896 on GPL570, GSE37147 on GPL13243, GSE130928 on GPL570, and GSE162635 on GPL570. Each dataset underwent standardization processing before analysis. Subsequent analyses were consistent with the RNA-seq Analysis section described in the Materials and Methods.

**GWAS Data sources**

The SNPs associated with the risk of FEV_1_/FVC, FEV_1_, FVC and doctor -diagnosed COPD were adjusted for age, sex, and genotype measurement batch. These SNPs were obtained from the IEU OpenGWAS project[21], which analyzes variables from the UK Biobank[22].

GWAS data were obtained from the UK Biobank[23] and included the following: FEV_1_/FVC ratio data from 321,047 individuals; FEV_1_ data from 321,047 individuals; FVC data from 321,047 individuals; and doctor-diagnosed COPD data from 353,315 individuals, comprising 1,658 cases and 110,925 controls. Notably, all GWAS datasets were obtained from the IEU GWAS database (https://gwas.mrcieu.ac.uk/), and the detailed information is provided in Supplemental Table 6.

**Instrumental variable (IV)**

eQTLs associated with IL6-AS1 were used as proxies for elevated IL6-AS1 exposure. Significant SNP sites from the GTEx_v8 database[24] were selected as IVs for IL6-AS1 expression in lung tissue, while SNP sites from the eQTLGen database[25] were used as IVs for IL6-AS1 expression in peripheral blood. The lung IVs included 120 SNPs, and the peripheral blood IVs included 57 SNPs.

**Summary-data-based Mendelian (SMR) randomization analyses**

The SMR and HEIDI (Heterogeneity In Dependent Instruments) tests were performed within cis regions using the SMR software[26]. This approach, rooted in the original SMR framework, utilizes a single-nucleotide variant (SNV) at a primary cis-eQTL as an instrumental variable (IV). By leveraging this IV alongside summary-level eQTL and GWAS data, the analysis explores potential causal or pleiotropic relationships between gene expression and various traits. However, the SMR method has limitations in differentiating causal associations from pleiotropy due to reliance on a single IV. The HEIDI test mitigates this issue by identifying linkage effects, which are generally considered less biologically relevant.

Additionally, summary-level sQTL data were utilized as the exposure variable for the SMR and HEIDI analyses. The SMR software's standard parameters were applied, which included a *p-value threshold of 5.0 × 10⁻⁸* for identifying prominent eQTLs and a 1 Mb window around the probe center for selecting cis-eQTLs. The analyses focused exclusively on cis regions, with a *p-value threshold of ≤ 0.05* indicating statistical significance in the SMR tests based on the utilization of a single probe. In the case of HEIDI, *a p-value of less than 0.05* was considered significant, suggesting that the identified association was unlikely to be attributed solely to causality.

**Two sample MR**

The inverse variance weighted (IVW) method served as the primary technique for our two-sample Mendelian Randomization (MR) analysis, supplemented by additional methods such as MR Egger, weighted median, simple mode, and weighted mode[27]. We evaluated heterogeneity in causal effects using the Cochran Q statistic within both the IVW and MR Egger frameworks. *A* *p-value of less than 0.05* from Cochran's Q test indicated significant heterogeneity. To assess horizontal pleiotropy, we employed MR Egger regression and MR-PRESSO (Pleiotropy Residual Sum and Outlier). An MR Egger intercept that approached zero, accompanied by *a p-value greater than 0.05*, suggested the absence of directional horizontal pleiotropy. Additionally, MR-PRESSO was used to identify and remove outliers, allowing for a repeated MR analysis that aimed to minimize bias. To ensure that no single genetic variant disproportionately influenced the results, leave-one-out sensitivity tests were conducted. The positive estimates from the remaining variants supported the robustness of the analysis. Finally, the Steiger filtering method was utilized to confirm the causal direction between the variables. Statistical analyses were performed in R using the 'TwoSampleMR' package[27] to implement all methods. *A p-value < 0.05* was considered statistically significant, and when significant heterogeneity was detected, a random effects model was used to calculate IVW estimates.

**Colocalization Analysis**

Colocalization analysis was used to evaluate whether two traits share a causal variant within a given genomic region[28]. This method considers five hypotheses: H0 (no causal variant), H1 (causal variant for trait 1 only), H2 (causal variant for trait 2 only), H3 (distinct causal variants for both traits), and H4 (shared causal variant for both traits). Using the 'coloc' R package, Bayesian colocalization analysis was performed to estimate the probability of shared causative variants. We tested hypotheses H3 and H4 to explore the relationship between IL6-AS1 expression and COPD outcomes. *A posterior probability for H4 (PPH4) above 80%* was considered evidence of significant colocalization between the traits. The analysis targeted a genomic region spanning 1 Mb on either side of the IL6-AS1 gene, with the variant showing the lowest p-value in this region selected from GWAS data for further evaluation.

**Supplemental Figures and Tables**


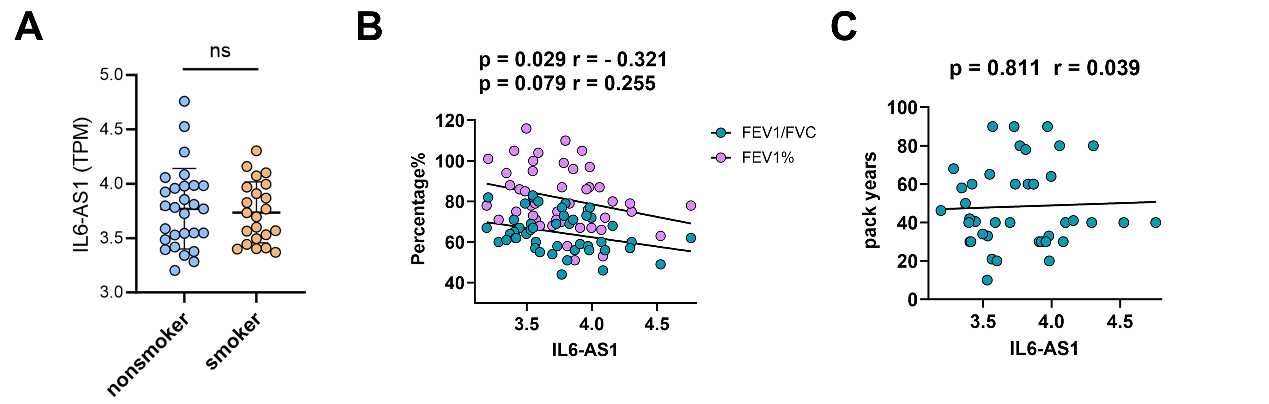


**Figure S1**

**A** Expression of IL6-AS1, in the lungs of non-smokers and smokers, taken from GSE103174.

**B-C** Analysis of the correlation between IL6-AS1 expression and various parameters, including FEV_1_%pre, FEV_1_/FVC ratio (B) and pack years (C) from GSE103174.

Data shown mean ± SD. *P values* shown in charts determined by two-tailed Mann–Whitney test (A), Pearson Correlation two-tailed (B-C).


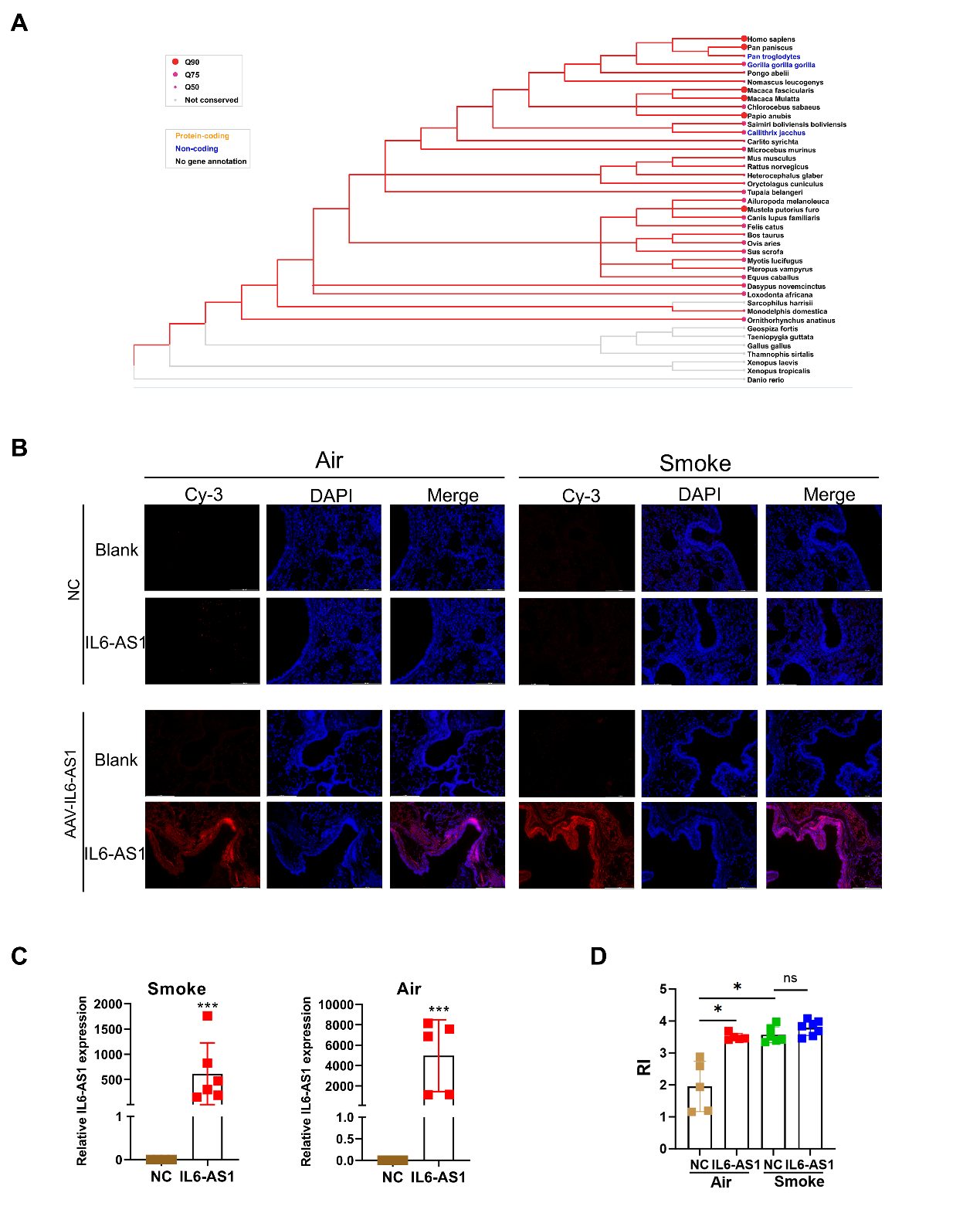


**Figure S2**

**A** Schematic representation of the conservation of IL6-AS1 across different species was assessed to determine its evolutionary conservation in AnnoLnc2 Database.

**B** FISH assay revealing *IL6-AS1* localization in mouse lung tissue sections. *IL6-AS1* is shown in red, and DAPI staining is shown in blue. (n=3 replicates, with 3 images observed).

**C** qRT-PCR analysis depicting *IL6-AS1* expression in wild-type and *IL6-AS1* mice. (n=5 for wild-type, n = 6 for *IL6-AS1* group).

**D** Lung RI performed in 4 groups of mice. RI, Respiratory Index. [(n=5, n = 7 (Smoke+IL6-AS1)]

Data shown mean ± SD. *P values* shown in charts determined by two-tailed Mann–Whitney test (C) and one-way ANOVA Bonferroni’s multiple comparisons test (D).


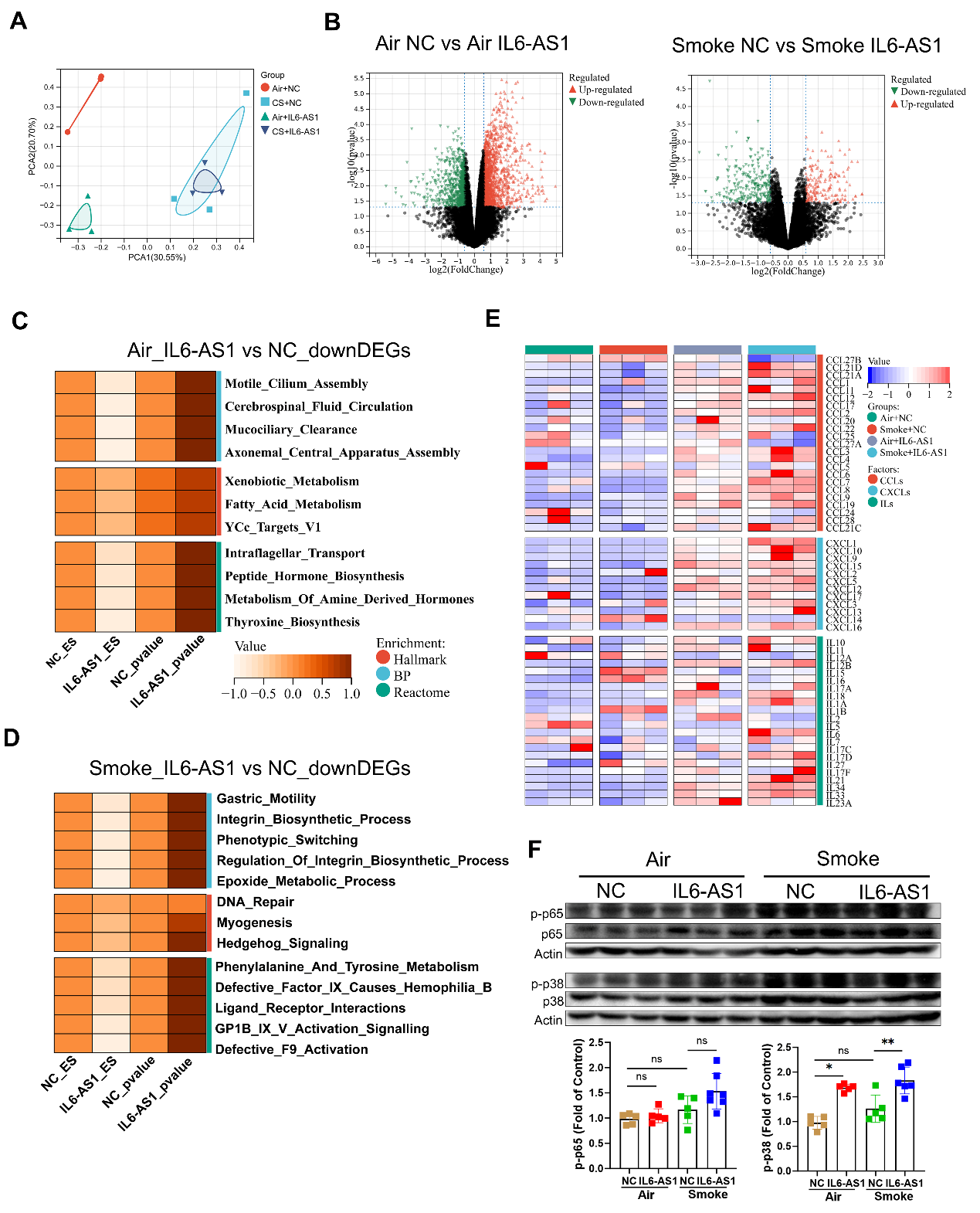


**Figure S3**

**A** The PCA plot of RNA-seq from the 4 groups of mice (n=3 in each group).

**B** Volcano plots illustrating RNA sequencing comparisons between Air+NC and Air+IL6-AS1 groups, and between Smoke+NC and Smoke+IL6-AS1 groups.

**C-D** Display of GSEA enrichment results for downregulated-DEGs in IL6-AS1 mice and wild-type mice from Air group (C) and Smoke group (D), across Hallmark, BP, and Reactome pathways. P-values are represented as log10 values.

**E** Heatmap illustrating the expression of CXCLs, ILs and CCLs families across the four mouse groups.

**F** Western blot analysis demonstrates the presence of phosphorylated p65 and p38 in the four distinct mouse groups, with Actin serving as a reference control. (n = 5 for wild-type, n = 7 for Smoke+ *IL6-AS1*).

Data shown mean ± SD. *P values* shown in charts determined by one-way ANOVA Bonferroni’s multiple comparisons test (F).


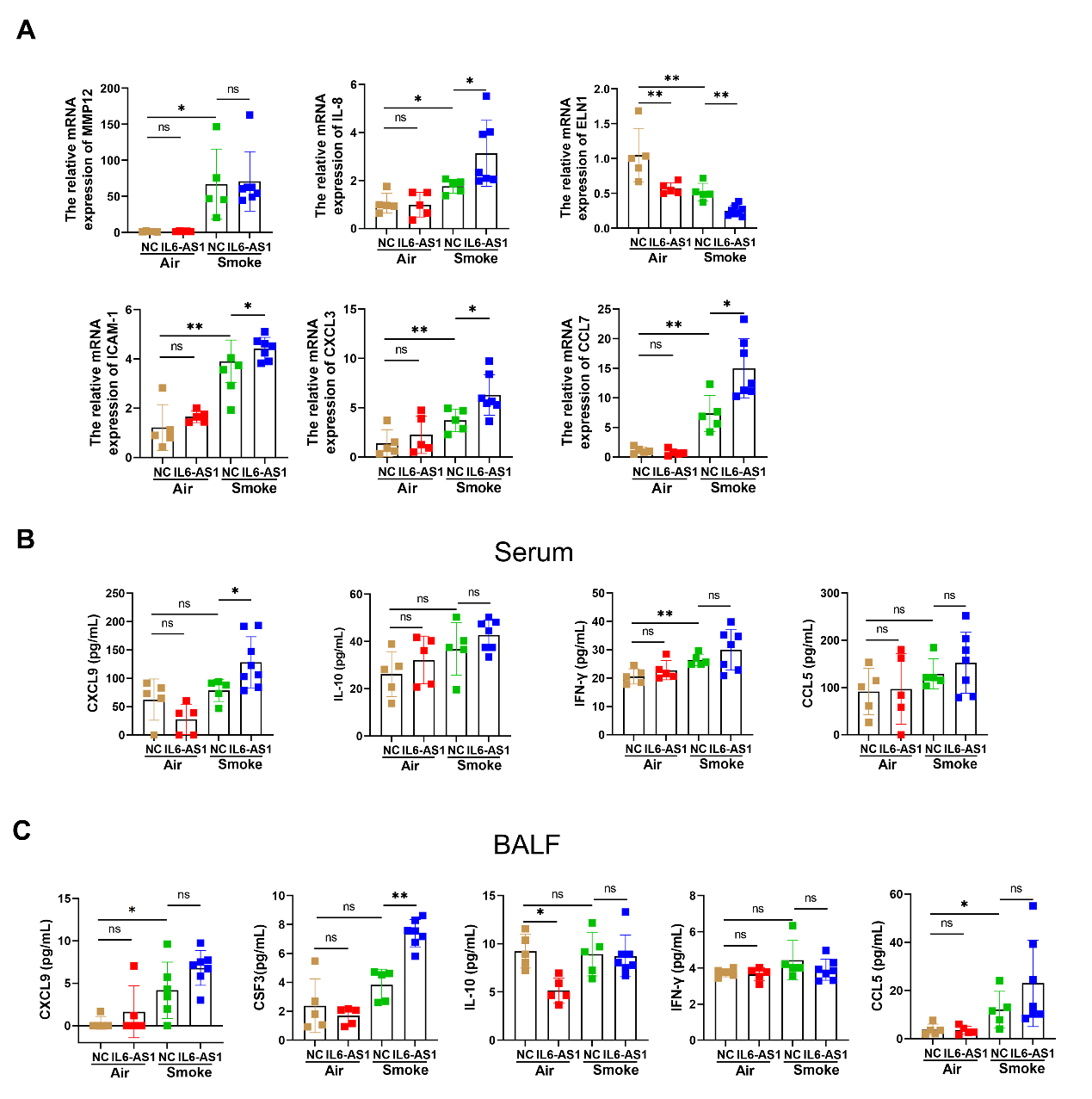


**Figure S4**

**A** qRT-PCR analysis of MMP-12, CXCL15(IL-8), ELN1, ICAM-1, CXCL-3 and CCL-7 in 4 groups mice. (n=5, n = 7 (Smoke+IL6-AS1))

**B** The secretion of CXCL-9, IL-10, IFN-γ, CCL-5 in serum were detected by CBA array. (n=5, n = 7 (Smoke+IL6-AS1))

**C** The secretion of CXCL-9, CSF-3, IL-10, IFN-γ, CCL-5 in BALF were detected by CBA array. (n=5, n = 7 (Smoke+IL6-AS1))

Data shown mean ± SD. *P values* shown in charts determined by two-tailed Mann–Whitney test (A) and one-way ANOVA Bonferroni’s multiple comparisons test (A-C).


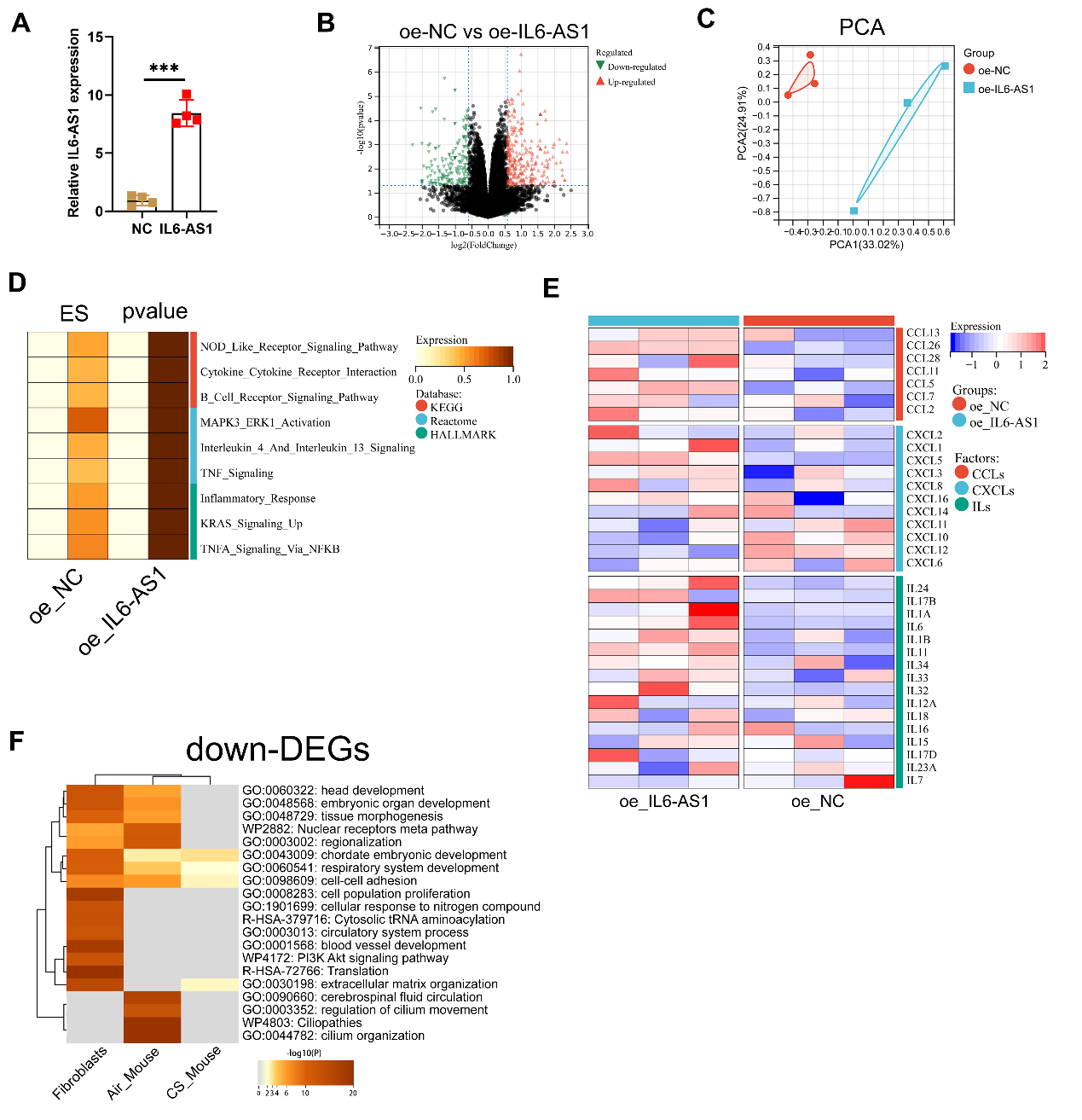


**Figure S5**

**A** qRT-PCR analysis of IL6-AS1 expression after IL6-AS1 overexpression using overexpression vector in HFL1 cells. (n=4 biological replicates, Student’s t-test)

**B-C** The　volcano plot　**(B)** and PCA plot **(C)** of RNA-seq between oe-NC and oe-IL6-AS1 groups (n=3 in each group).

**D** Display of GSEA enrichment results for downregulated-DEGs in IL6-AS1-overexpressing HF1 cells, across Hallmark, BP, and Reactome pathways. P-values are represented as log10 values.

**E** Heatmap showing the expression of CXCLs, ILs, and CCLs families in IL6-AS1-overexpressing HF1 cells.

**F** Metascape enrichment results for differentially downregulated genes (E) in IL6-AS1 mice in the Air group, IL6-AS1 mice in the Smoke group, and genes upregulated following IL6-AS1 overexpression in HFL cells.

Data shown mean ± SD. *P values* shown in charts determined by paired two-tailed Student’s t-test (A).


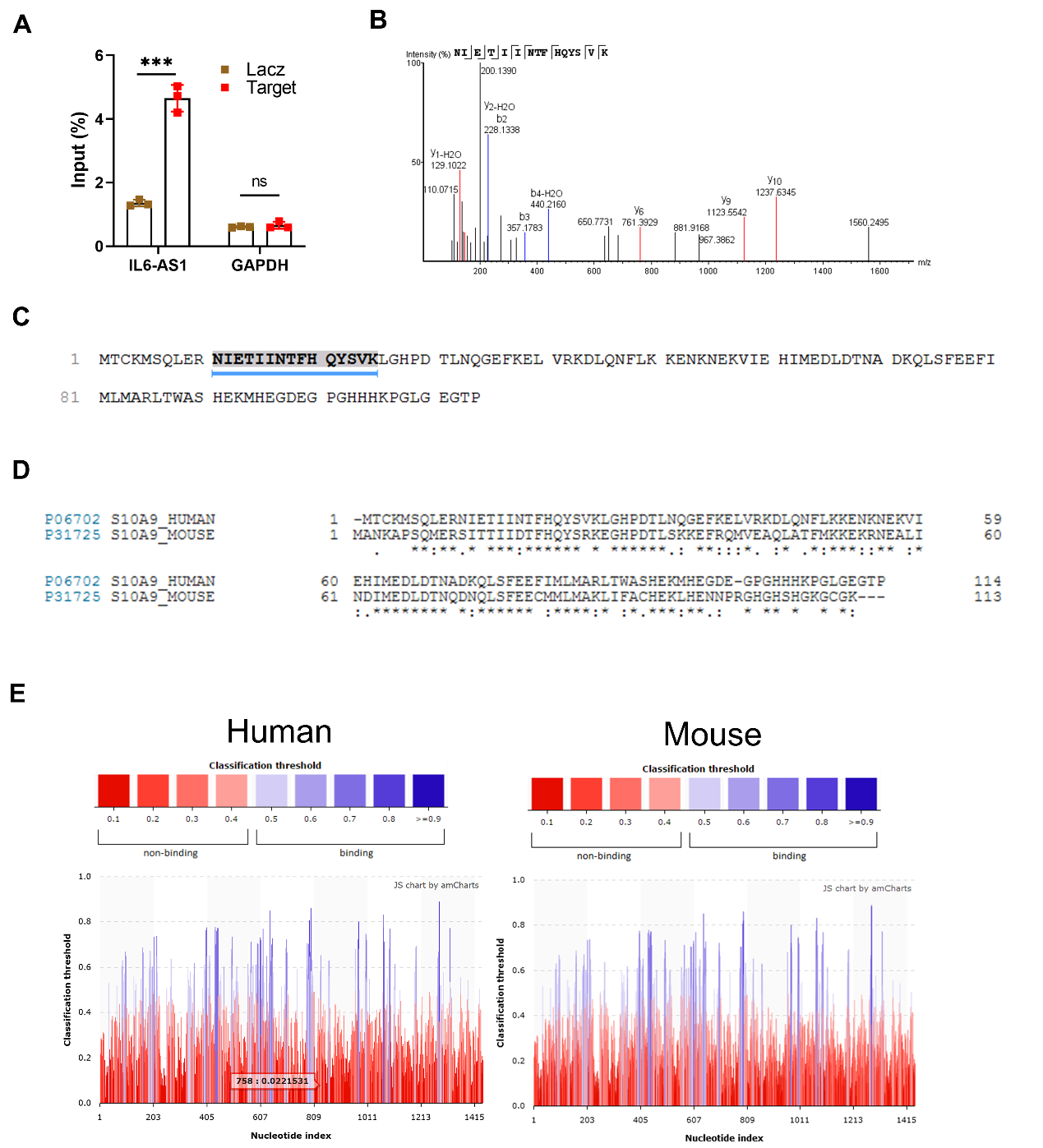
**Figure S6**

**A** The IL6-AS1 probe set was used as a ChIRP positive control; GAPDH was detected as a non-specific control (n=3 biological replicates).

**B-C** The peptide sequences of S100A9 protein in the results of mass spectrometric sequencing.

**D** The conservation of S100A9 protein between human and mouse.

E The bindings of IL6-AS1 to Human and Mouse S100A9 protein predicted via RNAInter.

Data shown mean ± SD. *P values* shown in charts determined by multiple two-tailed Student’s t-test (A).


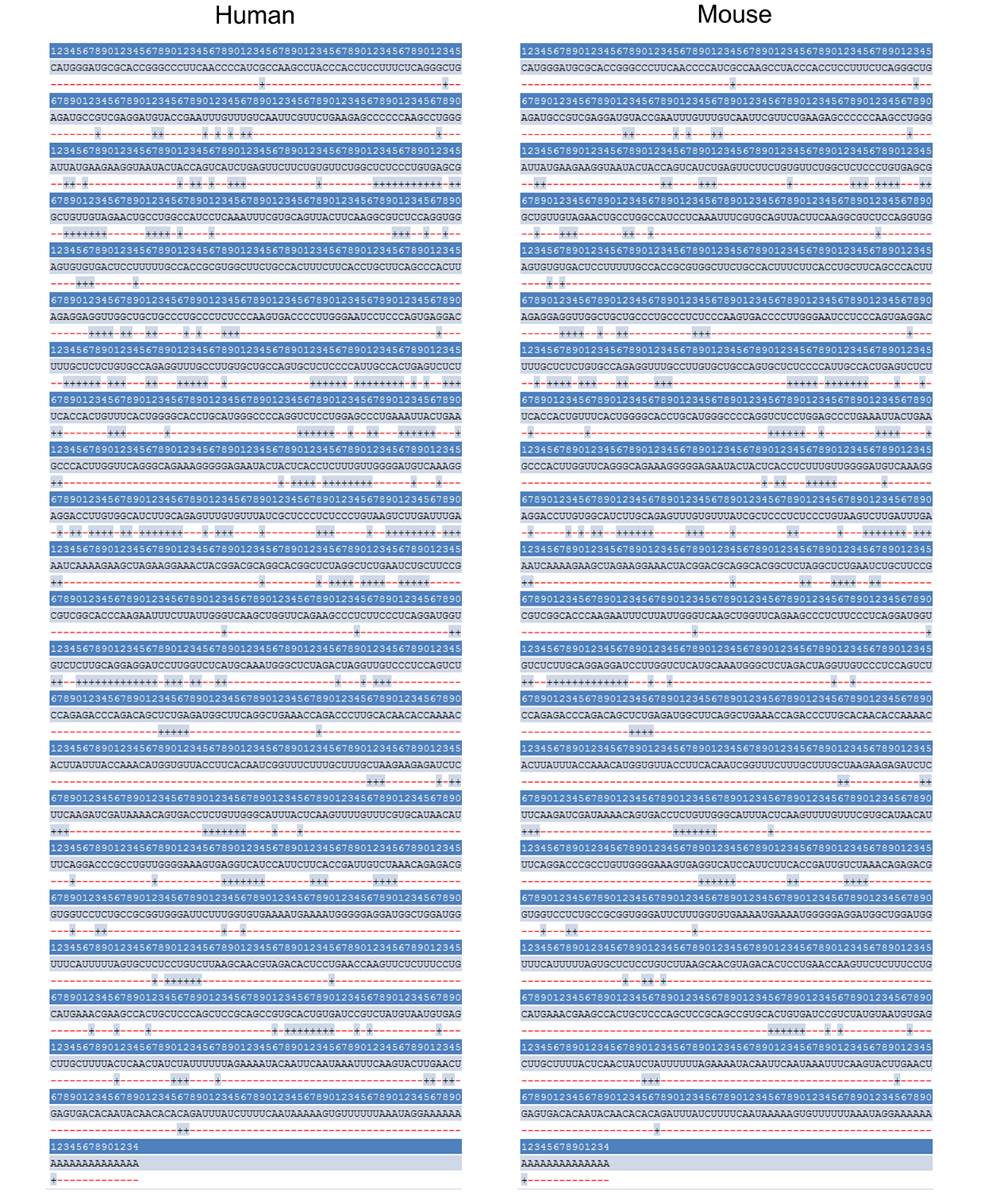


**Figure S7**

The binding of IL6-AS1 to Human and Mouse S100A9 protein were predicted by RPISeq (<http://pridb.gdcb.iastate.edu/RPISeq/index.html>).


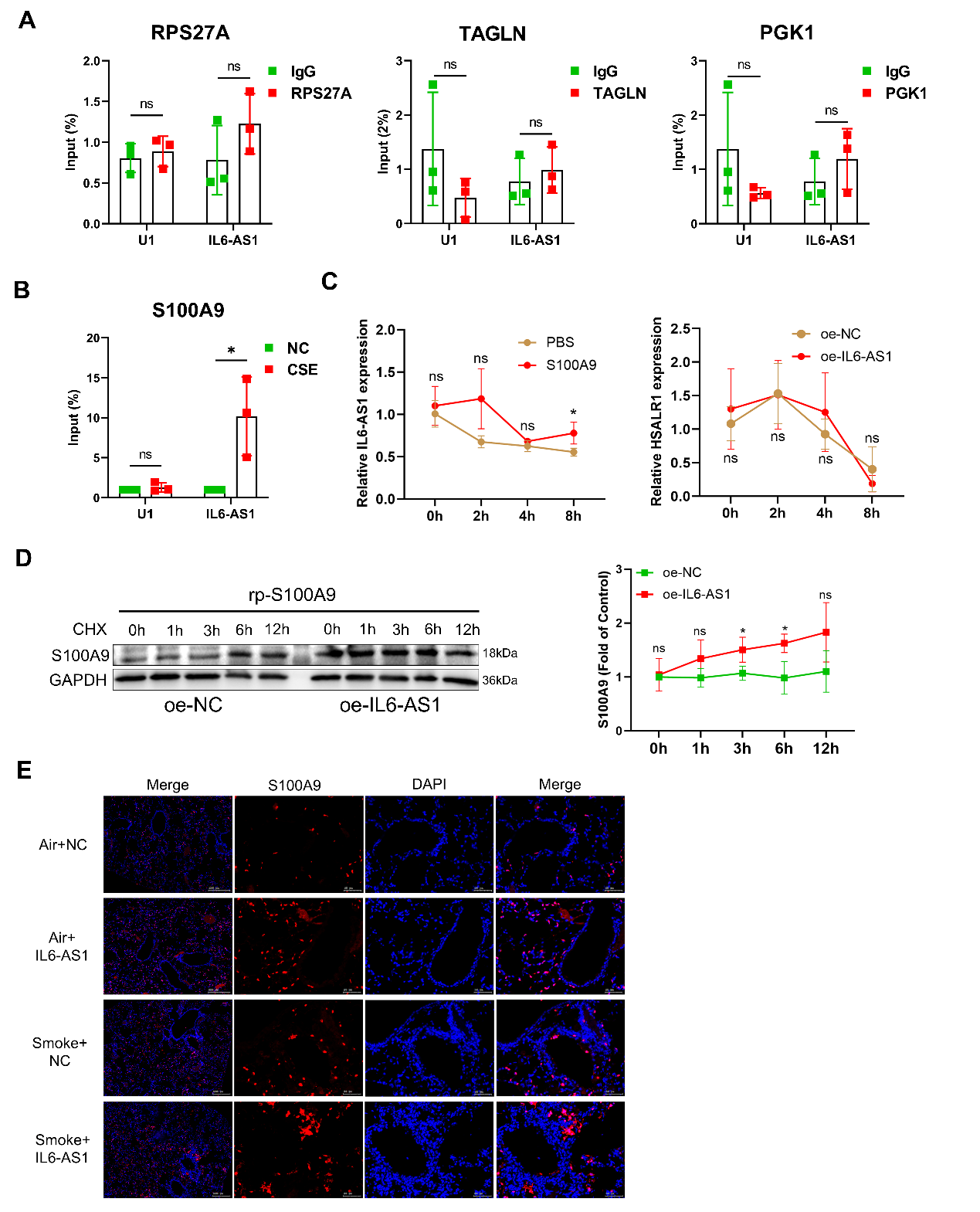


**Figure S8**

**A** RIP-qPCR analysis with anti-RPS27A antibody, anti-TAGLN antibody and anti-PGK1 antibody in HFL1 cells. Anti-IgG antibody serves and U1 as a negative control (n=3 biological replicates).

**B** RIP-qPCR analysis with anti-S100A9 antibody in HFL1 cells after stimulating with (n=3 biological replicates).

**C** HFL1 cells were treated with actinomycin D (Act D, 1 µg/mL) for the indicated time following stimulating with negative control (PBS) or rp-S100A9. IL6-AS1 and lncRNA HSALR1 expression was measured by qRT-PCR. (n=3 biological replicates).

**D** Forty-eight hours after the overexpression of IL6-AS1 and twenty-four hours after stimulation with rp-S100A9, HFL1 cells were treated with cycloheximide (CHX, 40μg/ml) and a negative control (DMSO), then collected at intervals of 0, 1, 3, 6, 12h, using GAPDH as the reference. (n=3 biological replicates).

**E** IF staining was performed by using anti-S100A9 antibody in mouse lung tissue sections from 4 groups of mice. Red: S100A9; Blue: DAPI. (n=3)

Data shown mean ± SD. *P values* shown in charts determined by multiple two-tailed Student’s t-test (A, B) and one-way ANOVA Tukey’s multiple comparisons test (C, D).


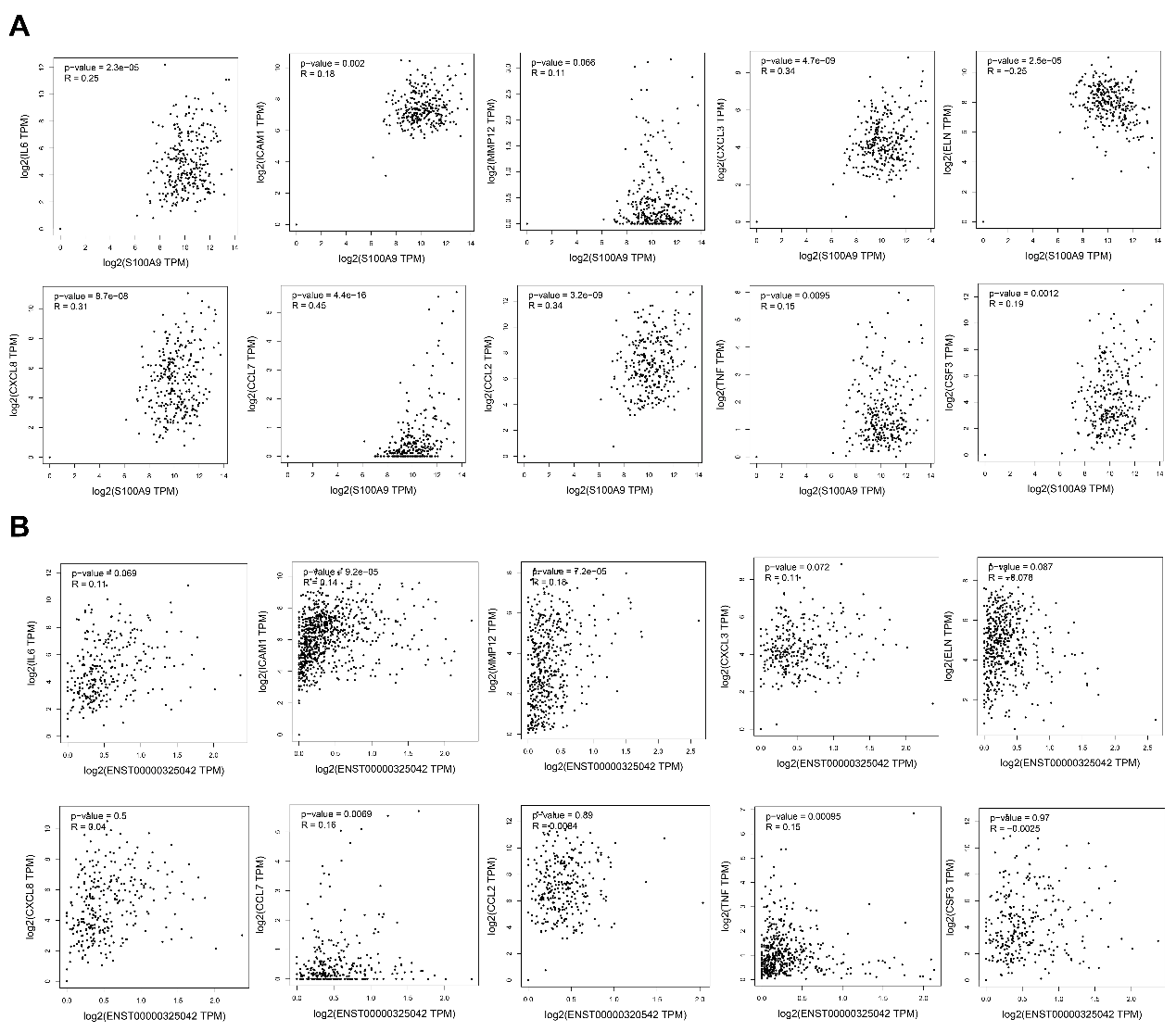


**Figure S9**

**A** Correlation analysis between the expression of IL6-AS1 and related DEGs (IL-6, ICAM-1, MMP12, ELN1, CXCL8, CSF3, CCL2, CXCL3, CCL7, TNF-α) in lung tissues based on GEPIA database.

**B** Correlation analysis between the expression of S100A9 and related DEGs (IL-6, ICAM-1, MMP12, ELN1, CXCL8, CSF3, CCL2, CXCL3, CCL7, TNF-α) in lung tissues based on GEPIA database.

*P values* shown in charts determined by Pearson Correlation two-tailed (A, B).


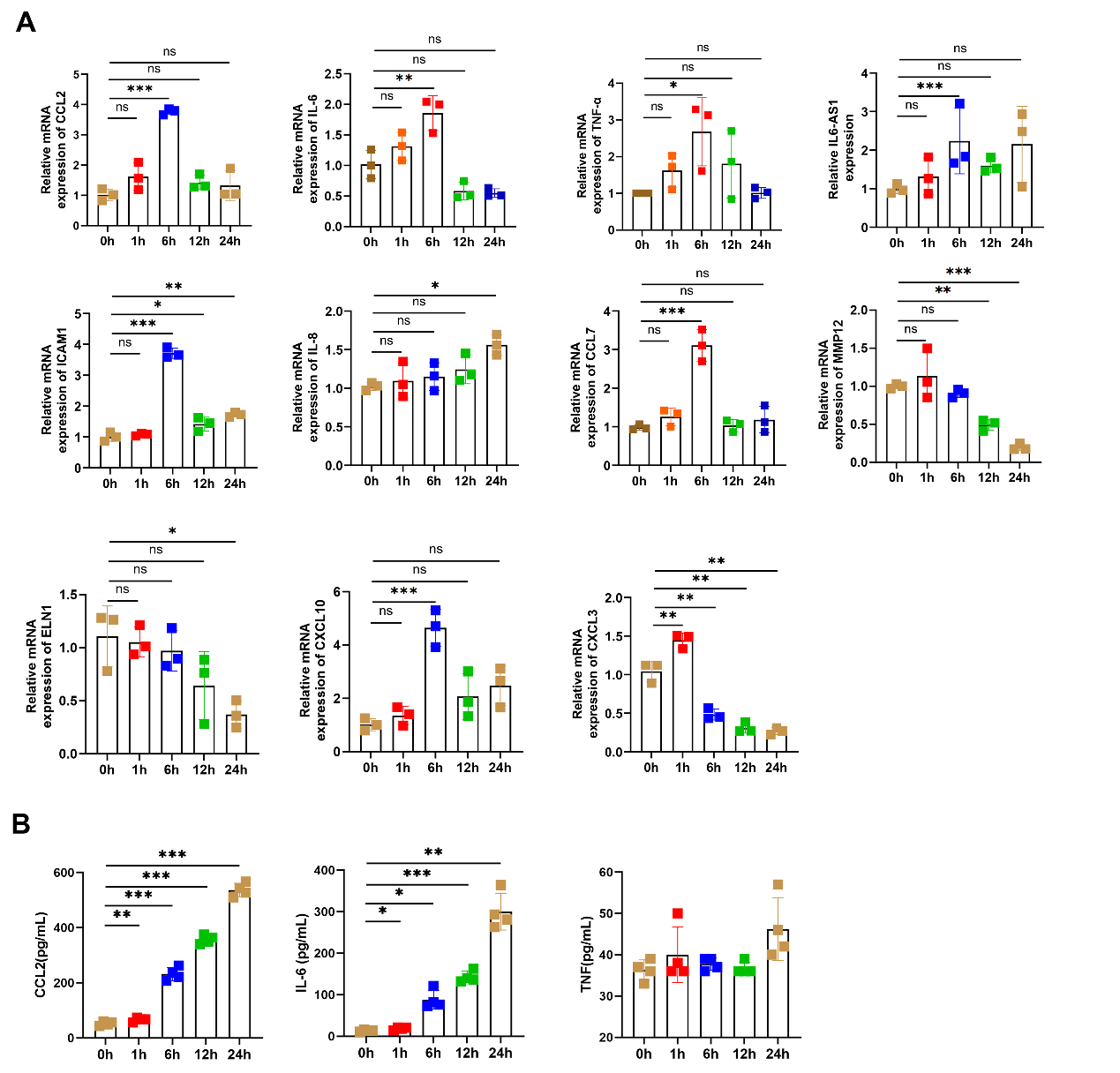


**Figure S10**

A qRT-PCR assay of the expression of IL6-AS1 and related DEGs (CCL2, IL-6, TNF-α, IL-8, ICAM-1, MMP-12, ELN1, CXCL8, CSF3, CXCL3, CCL7 and CXCL10) in HFL1 cells after stimulating with rp-S100A9 for 0, 1, 6, 12 and 24 hours. (n=3 biological replicates)

B The secretion of CCL2, IL-6 and TNF-α in HFL1 cells after stimulating with rp-S100A9 for 0, 1, 6, 12 and 24 hours were detected by ELISA assay. (n=4 biological replicates).

Data shown mean ± SD. *P values* shown in charts determined by one-way ANOVA Tukey’s multiple comparisons test (A, B).


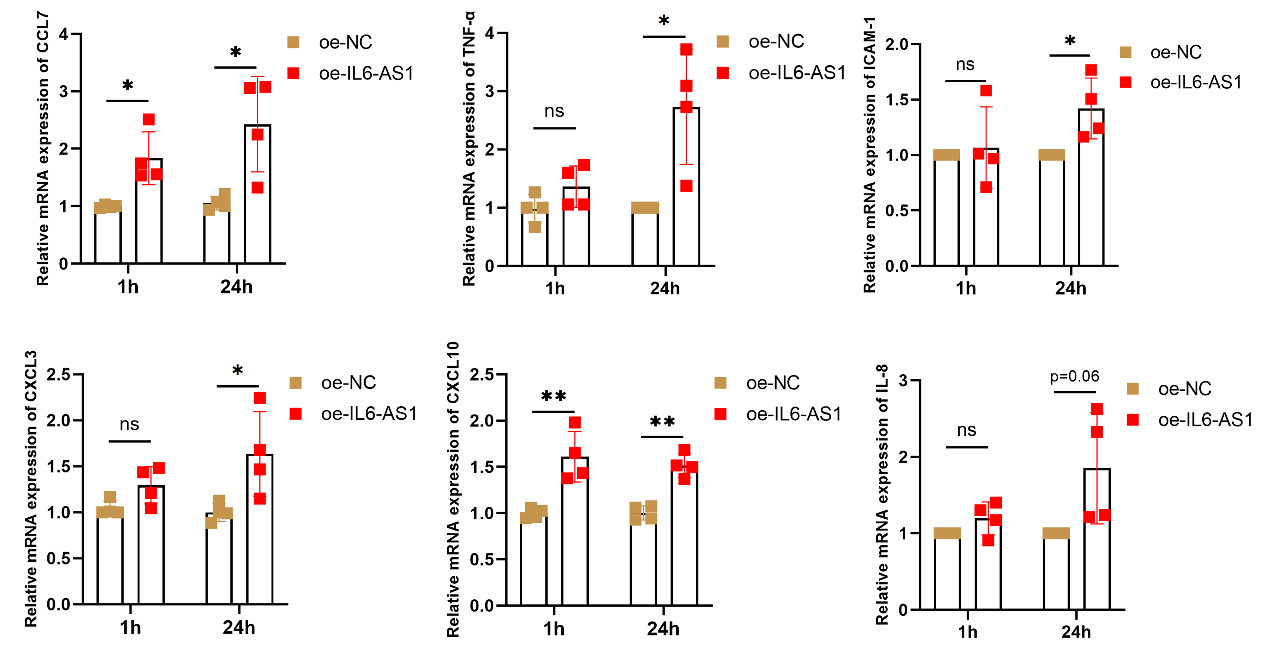


**Figure S11**

qRT-PCR assay of the expression of CCL7, TNF-α, ICAM-1, IL-8, CXCL3 and CXCL10 in IL6-AS1 overexpressed HFL1 cells after stimulating with rp-S100A9 for 1 and 24 hours. (n=4 biological replicates).

Data shown mean ± SD. *P values* shown in charts determined by multiple two-tailed Student’s t-test.


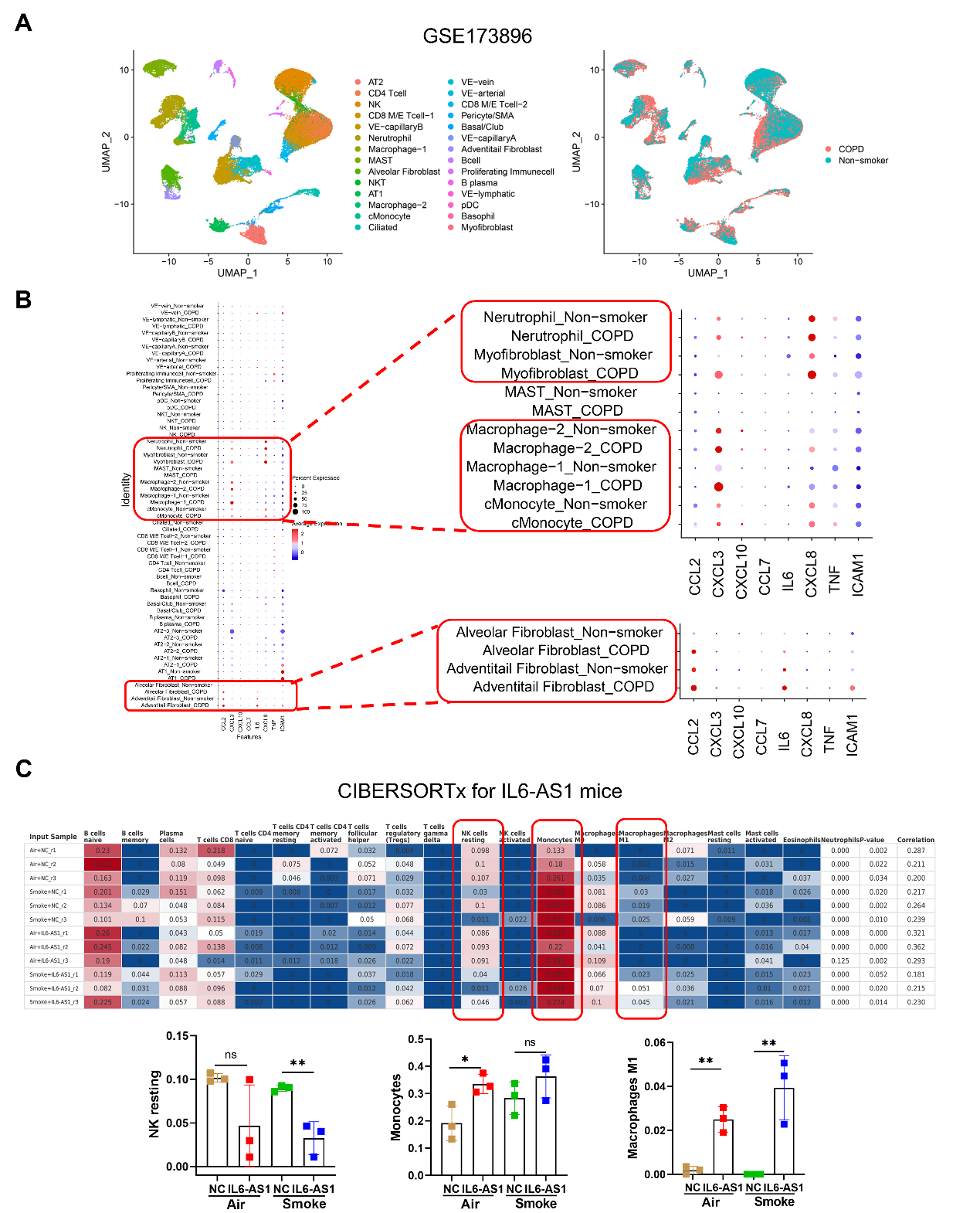


**Figure S12**

A Umap plot of single-cell transcriptomes from lung tissues between non-COPD and COPD patients from GSE173896.

**B** Dotplot of the expression of CCL-2/7, CXCL-3/8/10, IL-6, TNF and ICAM-1 in different cell types from GSE173896.

**C** Cell type deconvolution was performed using CIBERSORTx from the 4 groups of mice.

Data shown mean ± SD. *P values* shown in charts determined by multiple two-tailed Student’s t-test (C).


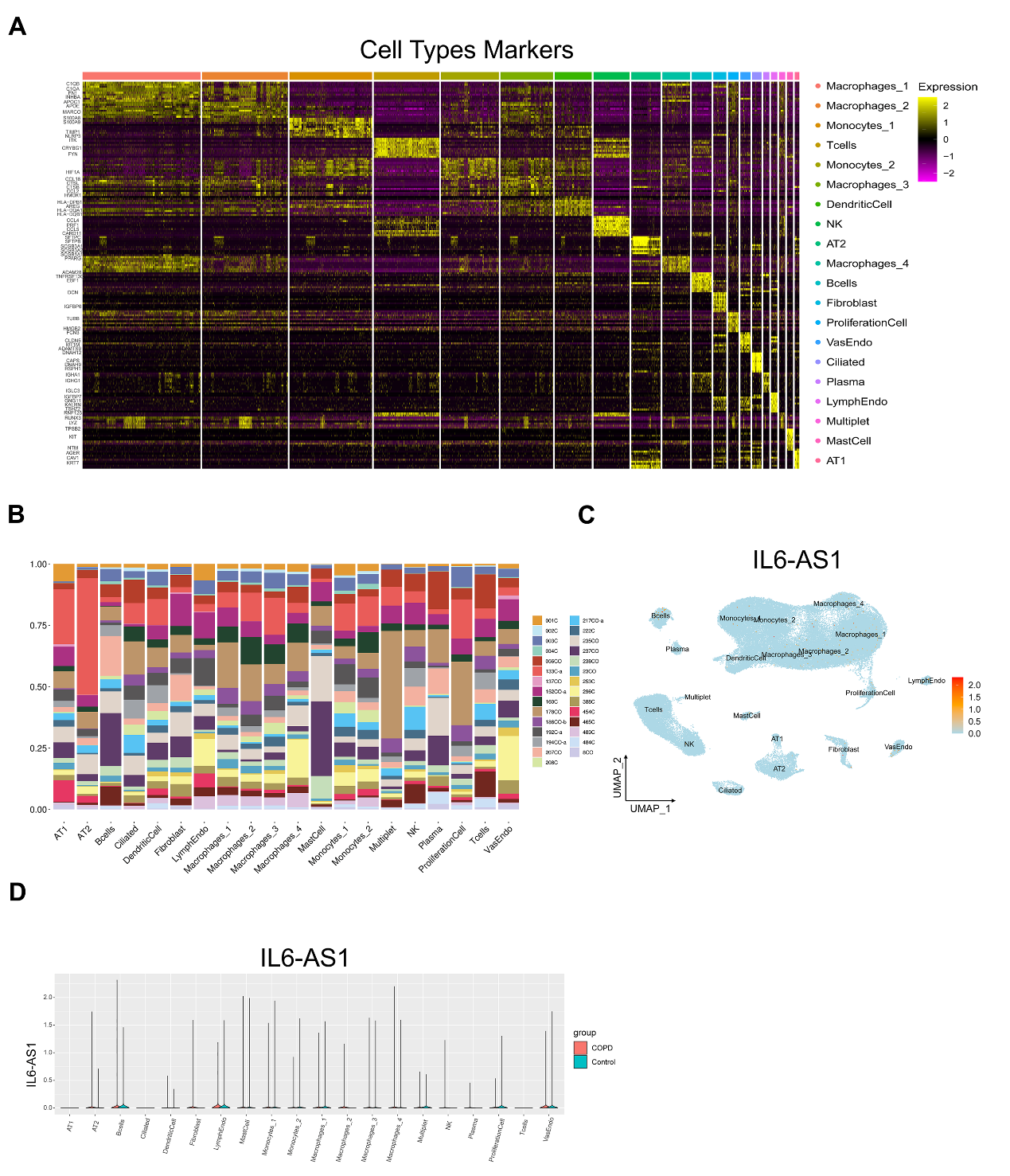


**Figure S13**

**A** Heatmap displaying marker genes across various cellular subgroups.

**B** Proportional Distribution of Cell Subgroups in Each Patient Sample.

**C** Umap plot illustrating the expression distribution of IL6-AS1 across single-cell transcriptomes.

**D** Expression of IL6-AS1 in different cellular subgroups.


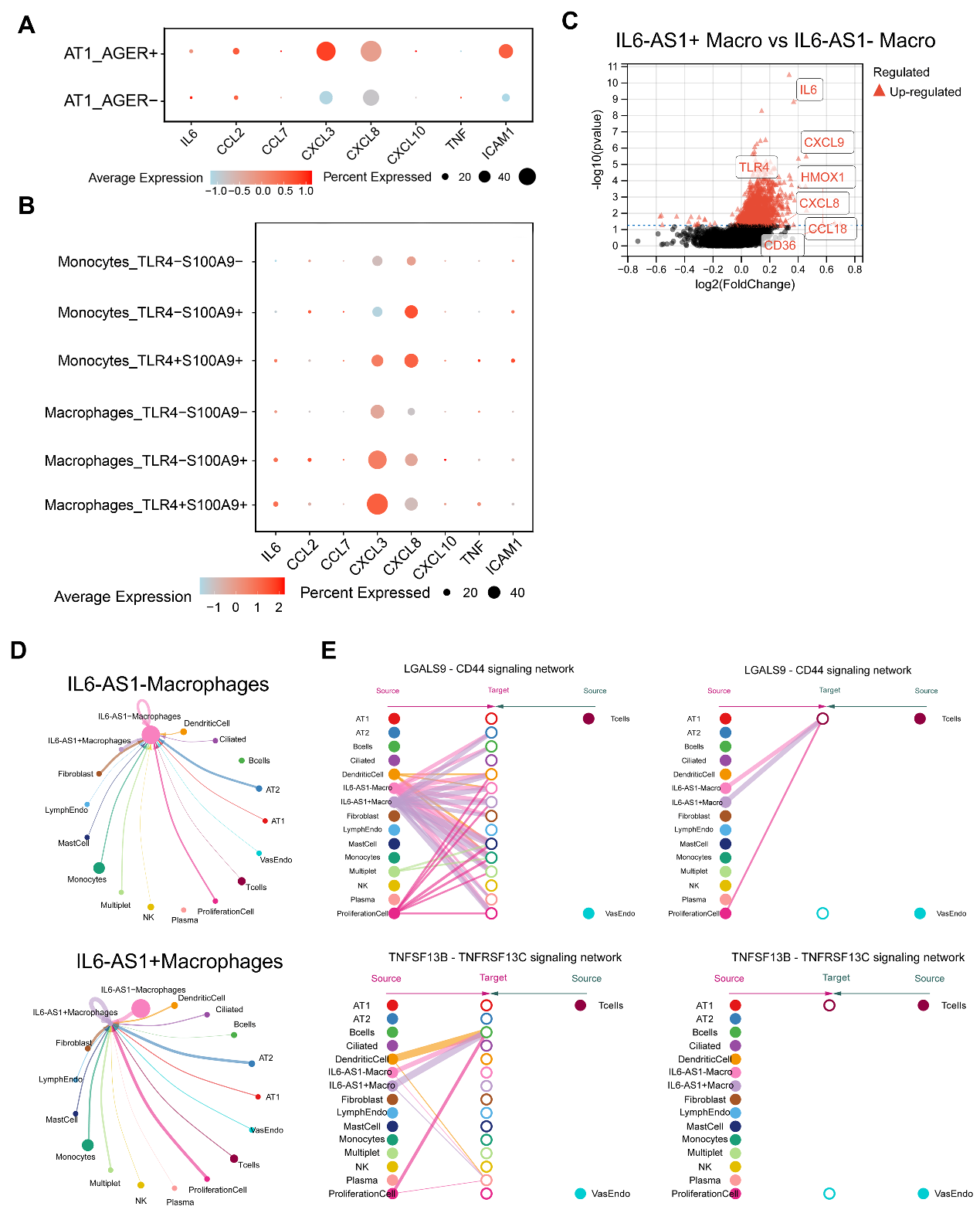


**Figure S14**

**A** Bubble chart illustrating the enrichment of related inflammatory genes (including IL-6, CCL2, CCL7, CXCL10, IL-6, ICAM-1, CXCL3, and TNF) in the distribution and expression profiles between AGER+ and AGER– AT1 cells, as identified in single-cell transcriptomes.

**B** Bubble chart illustrating the enrichment of related inflammatory genes (including IL-6, CCL2, CCL7, CXCL10, IL-6, ICAM-1, CXCL3, and TNF) in the distribution and expression profiles between TLR4-/S100A9-, TLR4-/S100A9+ and TLR4+/S100A9+ macrophages, as identified in single-cell transcriptomes.

**C** The volcano plot illustrates the DEGs between IL6-AS1+ and IL6-AS1- macrophages.

**D** Network graphs compare pulmonary cell interactions in IL6-AS1- (left) and IL6-AS1+ (right) macrophages as receptor cells. Each vertex represents a cellular subpopulation; edges signify ligand-receptor interactions. The thickness of the edges quantifies the cumulative expression of ligand-receptor genes, while the size of each vertex reflects Kleinberg centrality, indicating the cell's role in signaling. Cellular subpopulations are differentiated by color and number.

**E** Comparison of TNFSF13B-TNFSF13C and LGALS9-CD44 signaling interactions between IL6-AS1+ and IL-6AS1- macrophages and other cell subpopulations.


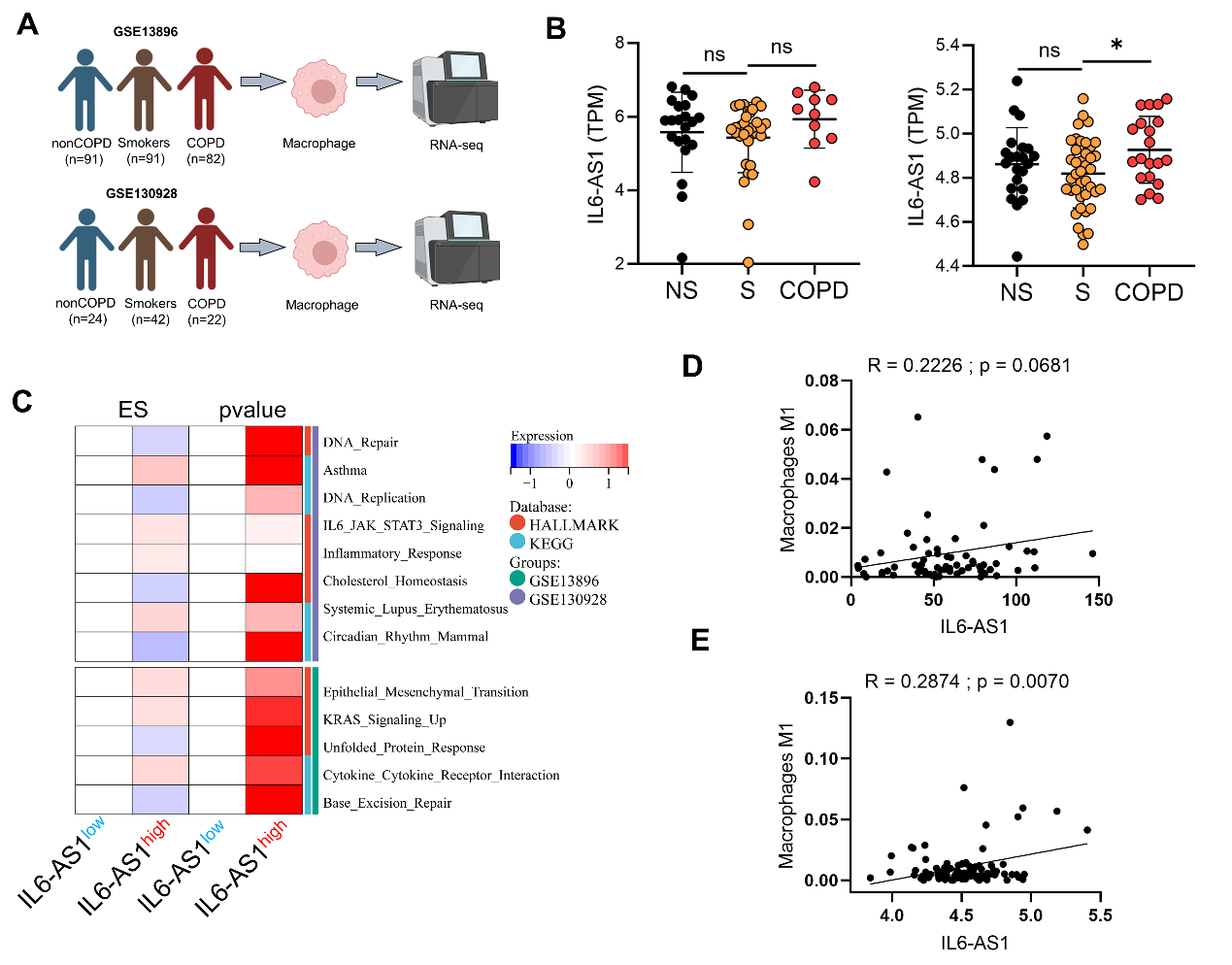


**Figure S15**

**A** RNA sequencing of macrophages from BALF of COPD patients retrieved from two independent cohorts (GSE13896 and GSE130928) in the GEO database.

**B** Expression of IL6-AS1, in the macrophages of non-smokers, healthy smokers and COPD patients, individuals are shown, taken from GSE13896 (n = 40 smokers and n = 111 COPD patients) (left) and GSE130928 (n = 18 smokers, n = 23 PRISm and n = 16 COPD patients) (right).

**C** A heatmap illustrates the most significantly enriched gene lists in the marcophages of populations with high and low IL6-AS1 expression, as determined by GSEA of the GO molecular function (MF) set using the GSE13896 and GSE 130928 dataset. P-values are represented as log10 values.

**D-E** Analysis of the correlation between IL6-AS1 expression and macrophage M1 score in CIBERSORTx scores from GSE13896 and GSE130928.

Data are presented as mean ± SD. *P values* in charts were determined by paired two-tailed Student's t-test (B), two-tailed Mann–Whitney test (F), and Pearson Correlation two-tailed (D-E).


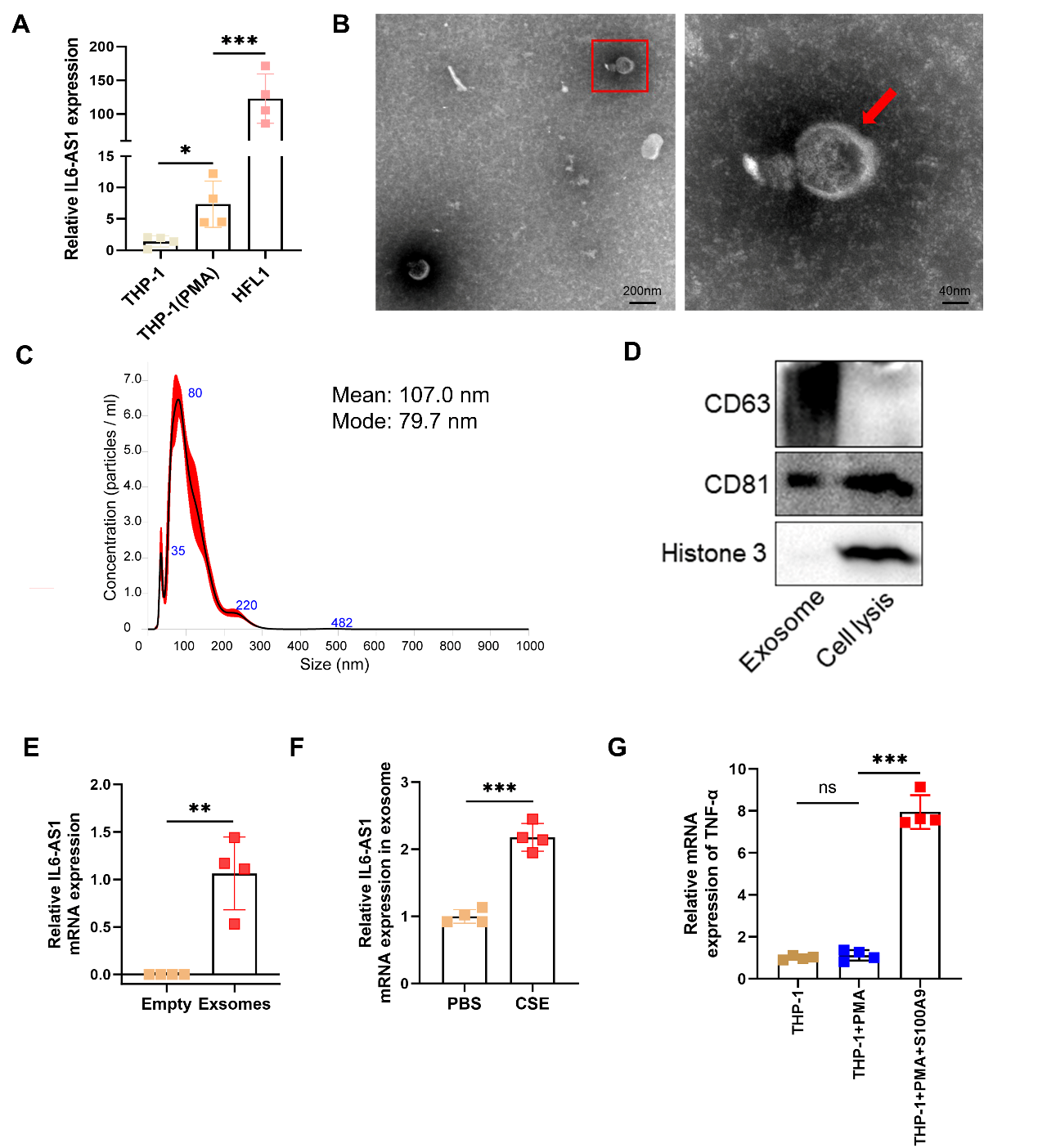


**Figure S16**

**A** Relative expression of IL6-AS in THP-1, PMA-stimulated THP-1, and HFL1 cells. (n=4 biological replicates)

**B** Representative images of HFL1 cell exosome structure captured by electron microscopy. Red arrows indicate the exosomes.

**C** Nanosight observation and detection of exosome particle size through particle size experiments.

**D** Western blot validation of CD81, CD63, and Histone 3 in exosomes and HFL1 cells.

**E** RT-qPCR assessment of IL6-AS1 levels in exosomes derived from HFL1 cells, using Empty as a negative control without cDNA. (n=4 biological replicates).

**F** RT-qPCR assessment of IL6-AS1 levels in HFL1 exosomes with and without CSE stimulation. (n=4 biological replicates).

**G** qRT-PCR assay of the expression of TNF-α in Thp-1 cells following co-culture after stimulating with PMA alone or PMA in combination rp-S100A9. (n=4 biological replicates).

Data are presented as mean ± SD. *P values* in charts were determined by paired two-tailed Student's t-test (A, E, F, G)


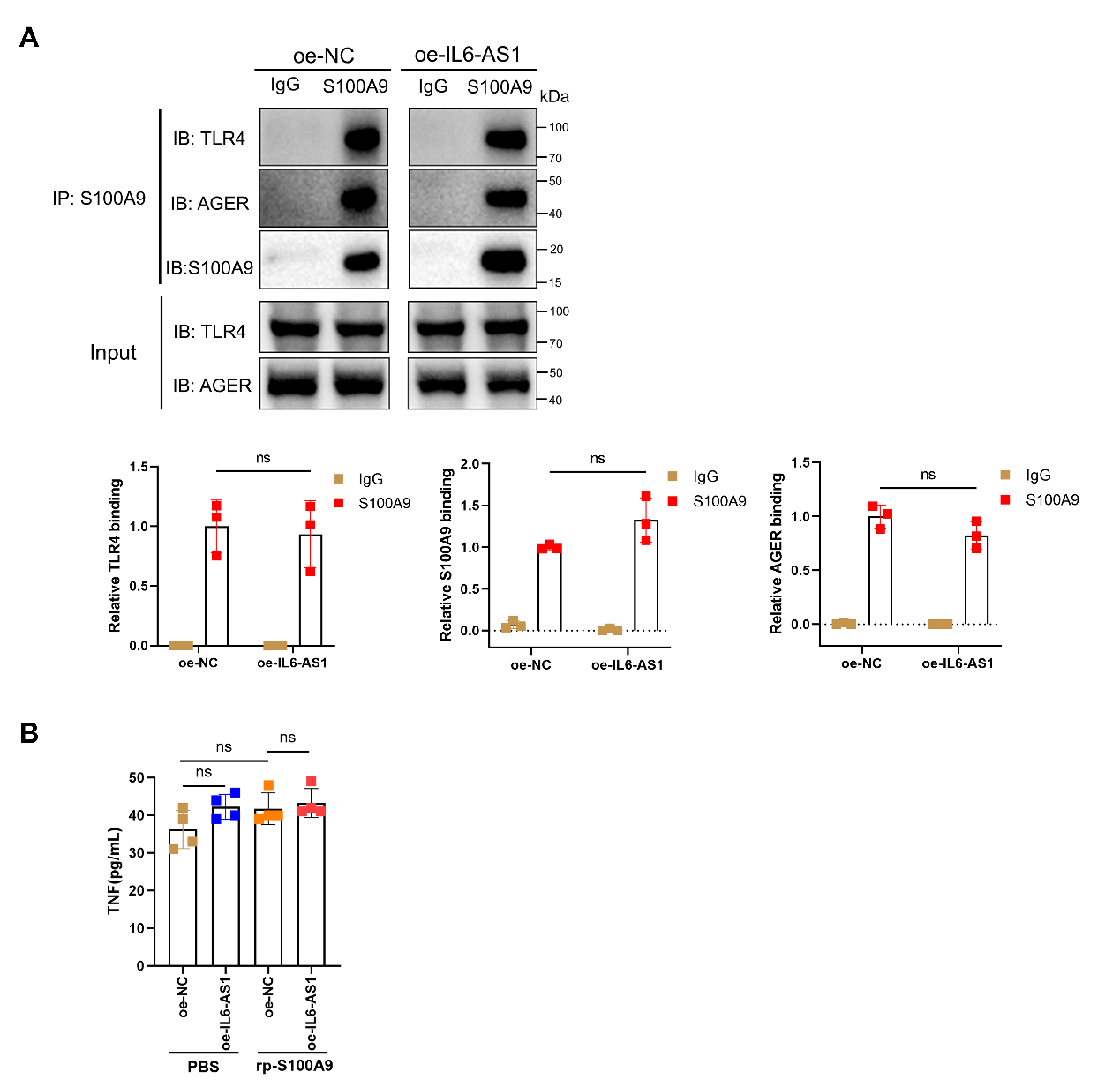


**Figure S17**

**A** Co-IP analysis of BEAS-2B co-cultured with IL6-AS1-overexpressed HFL1 using anti-S100A9 antibody. Western blot was used to verify the Co-IP results with anti-S100A9, anti-TLR4 and anti-AGER antibodies. (n=3 biological replicates)

**B** The ELISA assay was employed to detect the secretion of TNF-α in HFL1 cells following co-cultivation and stimulation with rp-S100A9. (n=4 biological replicates)

Data shown mean ± SD. *P values* shown in charts determined by one-way ANOVA Tukey’s multiple comparisons test (B) and multiple two-tailed Student’s t-test (A).


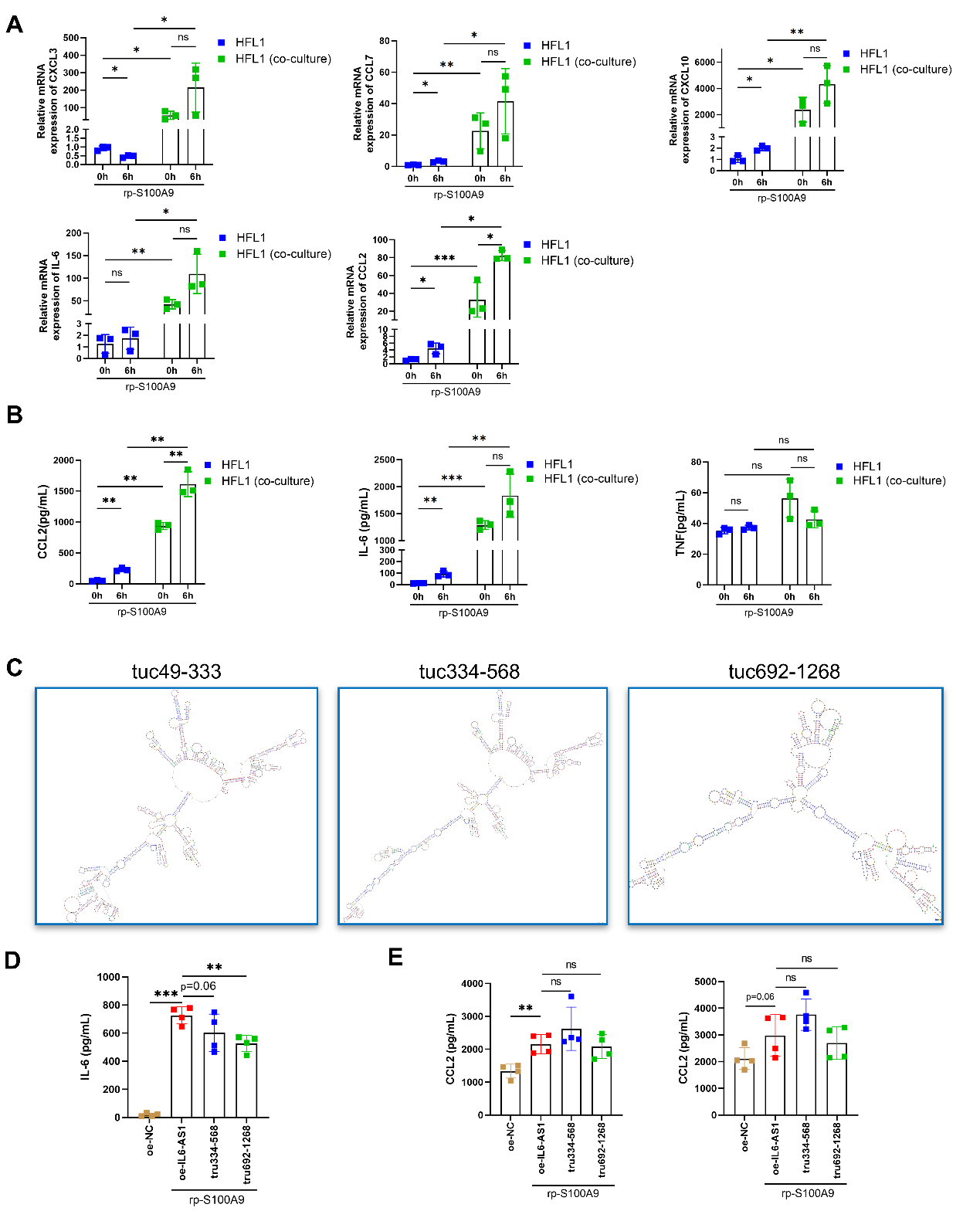


**Figure S18**

**A** qRT-PCR assay of the expression of CXCL-3/10, CCL-2/7 and IL-6 in isolated and co-cultured fibroblasts after stimulating with rp-S100A9. (n=3 biological replicates, Student’s t-test)

**B** The secretion of CCL-2, IL-6 and TNF in isolated and co-cultured fibroblasts after stimulating with rp-S100A9, were detected by ELISA array. (n=3 biological replicates)

**C** Schematic representation of three different truncated IL6-AS1 (tuc49-333, tuc334-568 and tuc692-1268) secondary structures are predicted.

**D** The secretion of IL-6 and CCL-2 in HFL1 cells and CCL-2 in THP-1 cells following co-culture after transfected with wily-type vector or truncation vector of *IL6-AS1* were detected by ELISA assay. (n=4 biological replicates).

Data shown mean ± SD. *P values* shown in charts determined by one-way ANOVA Tukey’s multiple comparisons test (D, E) and multiple two-tailed Student’s t-test (A, B).


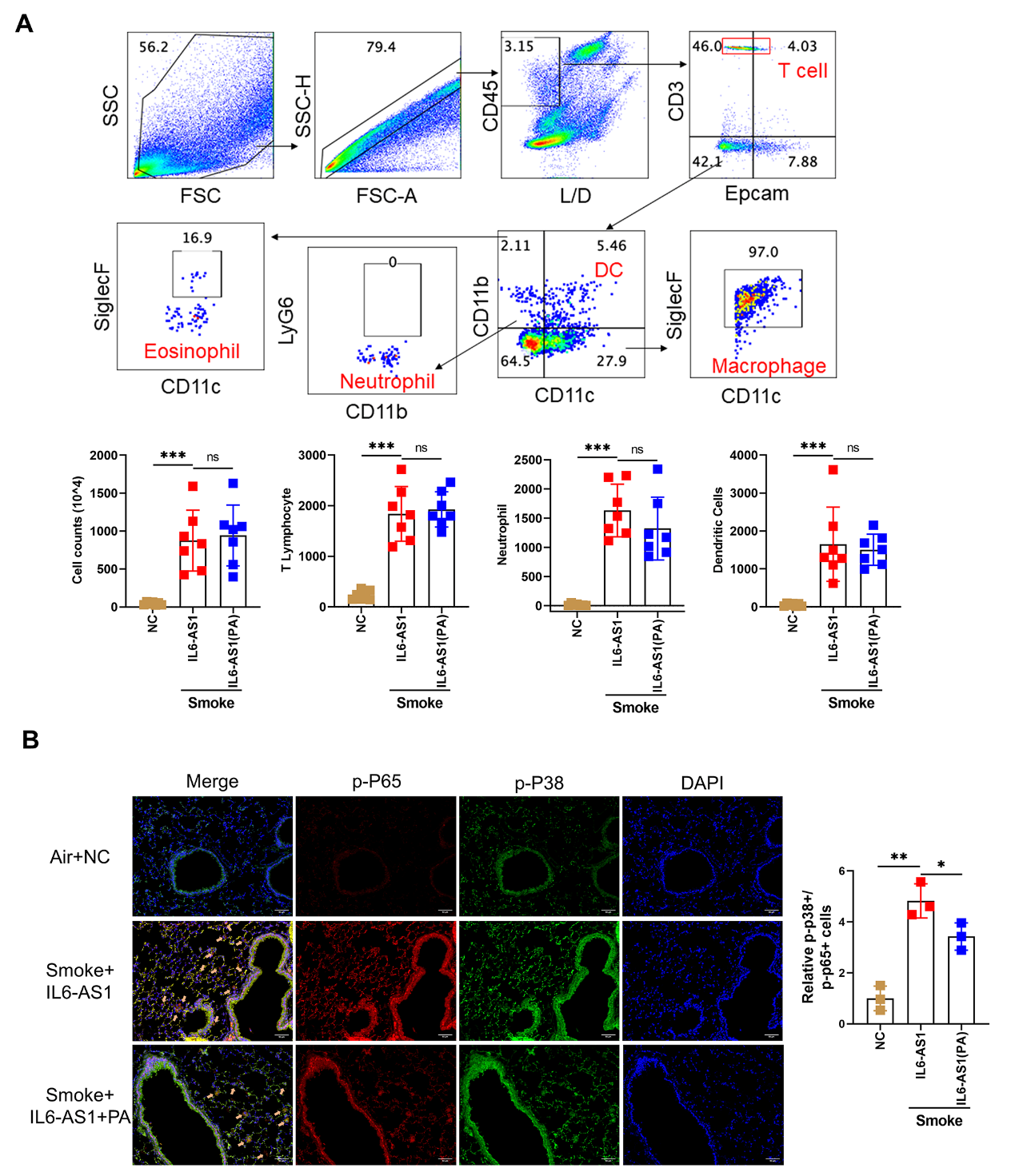


**Figure S19**

A The gating strategy of flow cytometry was shown and flow cytometry cell sorting was performed to quantify the total cell numbers and cell populations of T cells, Neutrophils and DC cells. (n=7, Student’s t-test)

B IF double staining was performed by using anti-phospho-p65/anti--phospho-p38 antibody in mouse lung tissue sections extracted from 3 groups of mice. (n=3)

Data shown mean ± SD. *P values* shown in charts determined by one-way ANOVA Tukey’s multiple comparisons test (A, B).


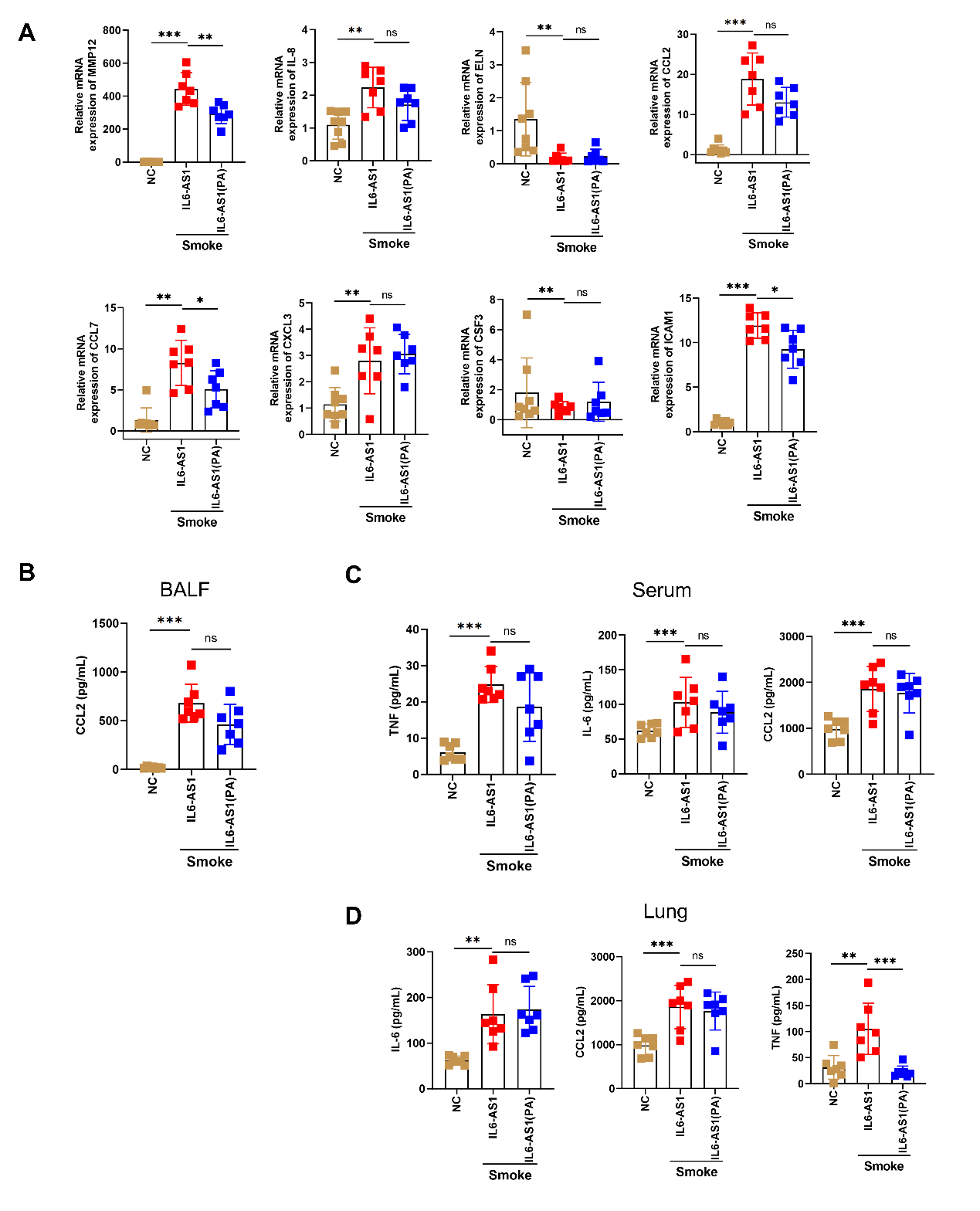


**Figure S20**

**A** qRT-PCR analysis of the expression of CCL-2/7, ICAM-1, MMP-12, IL-8, ELN, CXCL-3 and CSF-3 in 3 groups of mice. (n=7)

B The secretion of CCL-2 in BALF were detected by ELISA array. (n=7)

**C** The secretion of IL-6, CCL-2 and TNF-α in serum were detected by ELISA array. (n=7)

**D** The secretion of IL-6, CCL-2 and TNF-α in lung tissue homogenates were detected by ELISA array. (n=7)

Data shown mean ± SD. *P values* shown in charts determined by one-way ANOVA Tukey’s multiple comparisons test (A, B, C, D).


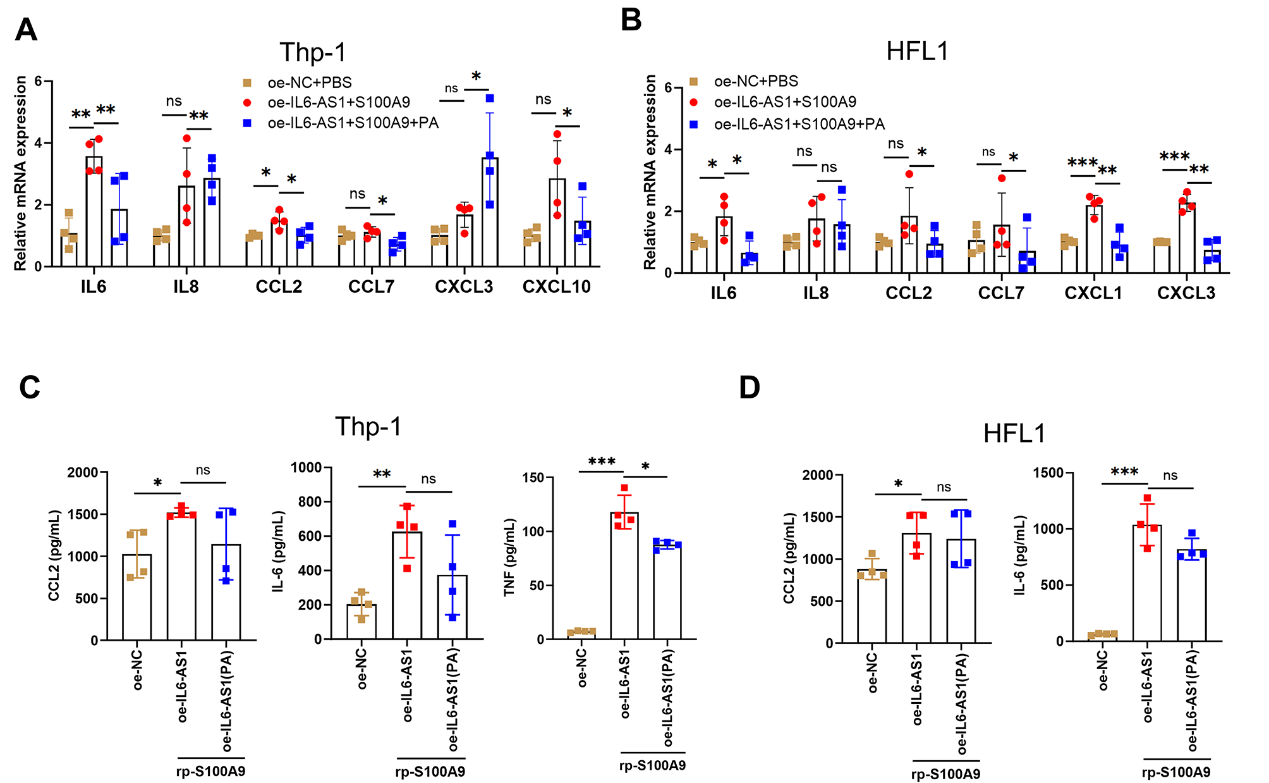


**Figure S21**

**A-B** qRT-PCR assay of the expression of inflammation-related genes (IL-6, IL-8, CCL-2/7 and CXCL-3/10) in THP-1 cells (A) and HFL1 (B) cells following co-culture after transfected with *IL6-AS1* overexpression vector for 48h, treatment with paquinimod and negative control (DMSO) for 2h and stimulating with rp-S100A9 for 6h. (n=4 biological replicates).

**C-D** The secretion of CCL2, IL-6 and TNF-α in THP-1 cells (C), and IL-6 and CCL2 in HFL1 cells (D), subsequent to co-culture, transfection with the *IL6-AS1* overexpression vector for 48 hours, post-treatment with paquinimod and negative control (DMSO) for 2 hours, and stimulation with rp-S100A9 for 6 hours, was ascertained through an ELISA assay. (n=4 biological replicates).

Data shown mean ± SD. *P values* shown in charts determined by one-way ANOVA Tukey’s multiple comparisons test (A, B, C, D).


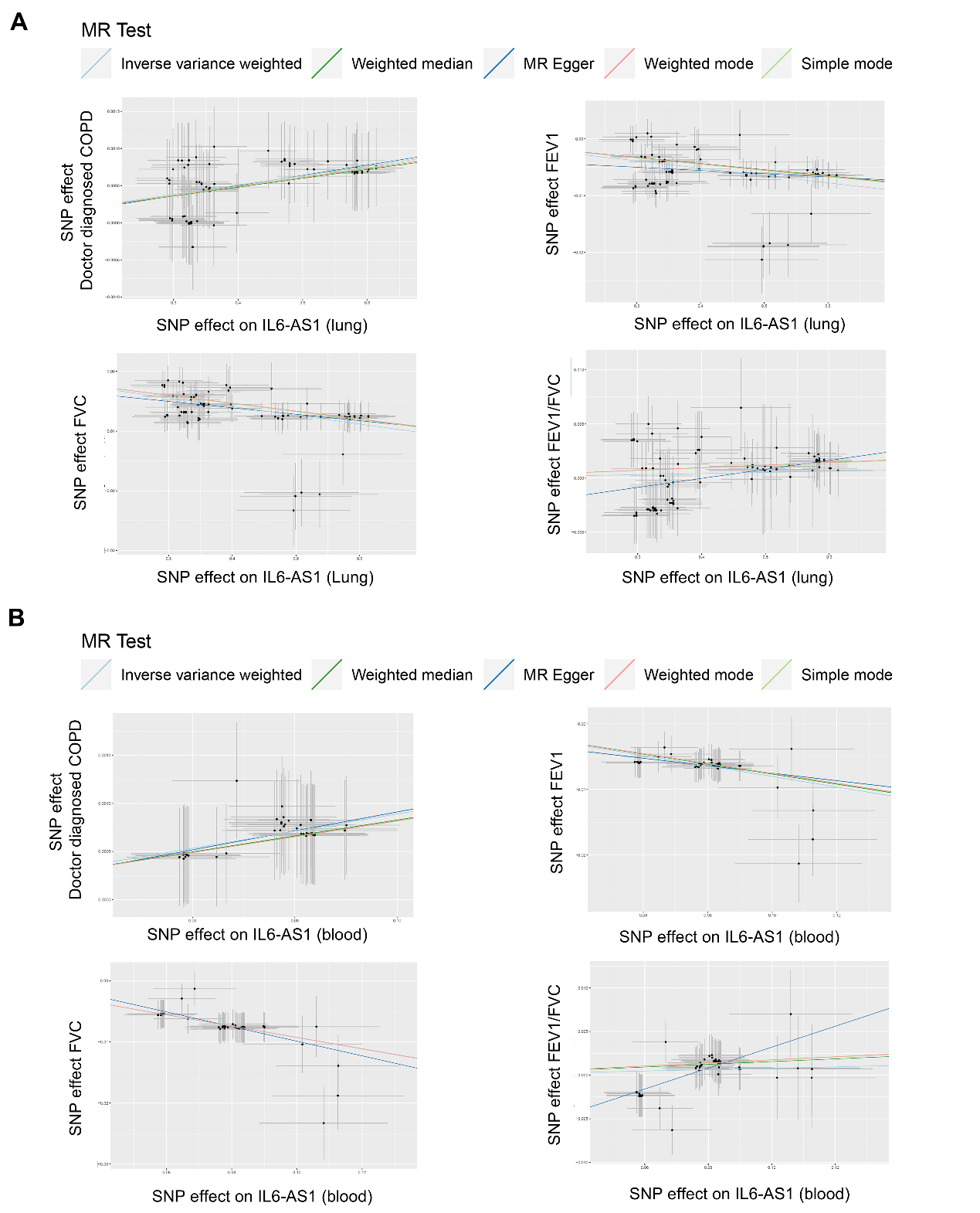


**Figure S22**

**A-D** Mendelian analysis results of scatter chart s used TwoSampleMR package on the association between the expression of IL6-AS1 in lung and doctor diagnosed COPD (A), FEV_1_ (B), FVC (C) and FEV_1_/FVC (D).

**E-H** Mendelian analysis results of scatter chart s used TwoSampleMR package on the association between the expression of IL6-AS1 in blood and doctor diagnosed COPD (E), FEV_1_ (F), FVC (G) and FEV_1_/FVC (H).


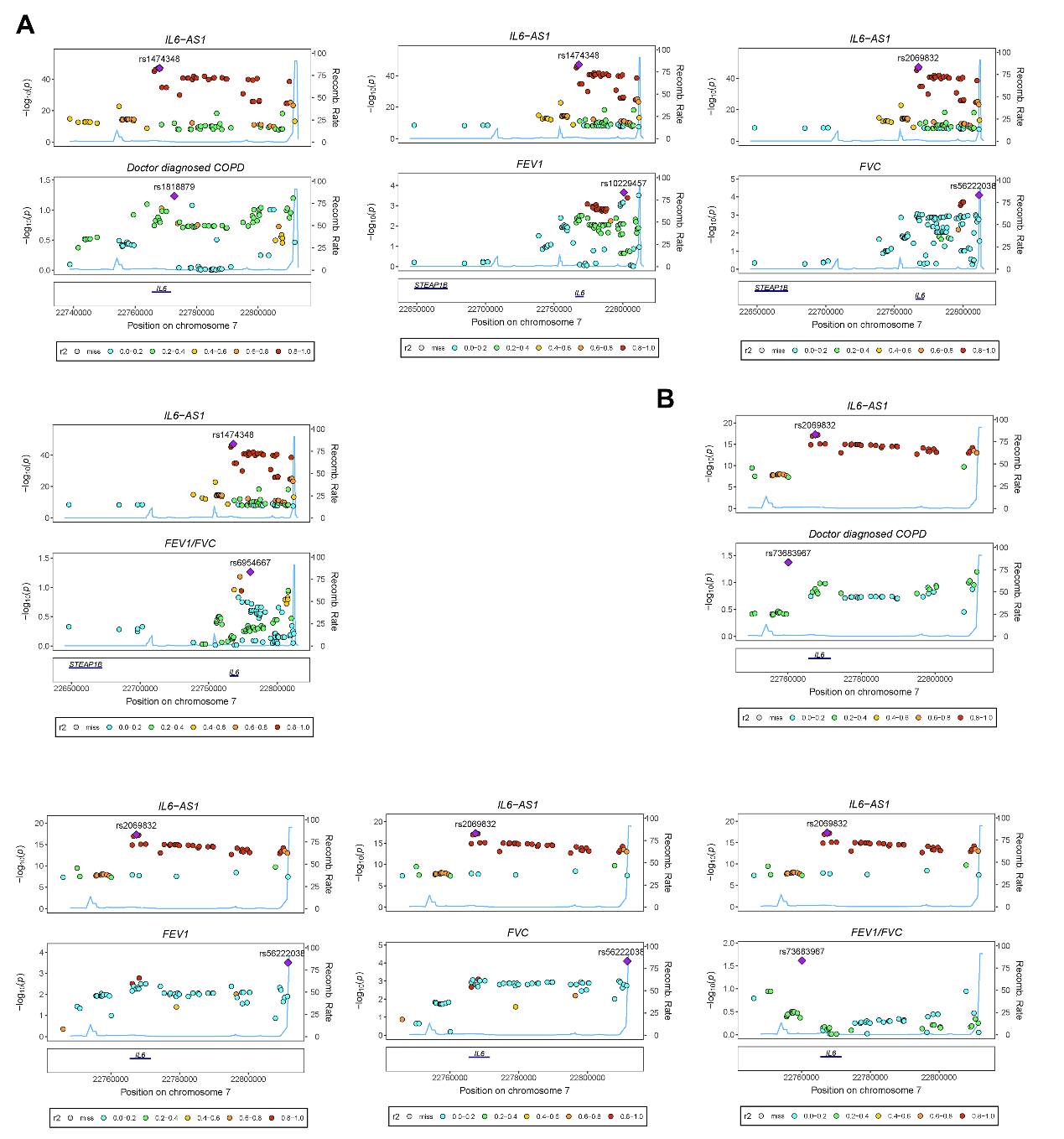


**Figure S23**

**A-B** Regional plots for the association with doctor diagnosed COPD, FEV1, FVC and FEV1/FVC within ± 1 MB of IL6-AS1 in lung **(A)** and in blood **(B)** from GTEx_v8 database showing LD patterns. The variants used as IVs in MR analysis were labeled and colored purple, the genomic positions (GRCh37) on chromosome 7 are shown on the x-axis, and *−log10 p-values* for SNVs from the GWAS on COPD associated phenotypes (top) and eQTL study for IL6-AS1 gene (bottom) are shown on the y-axis.


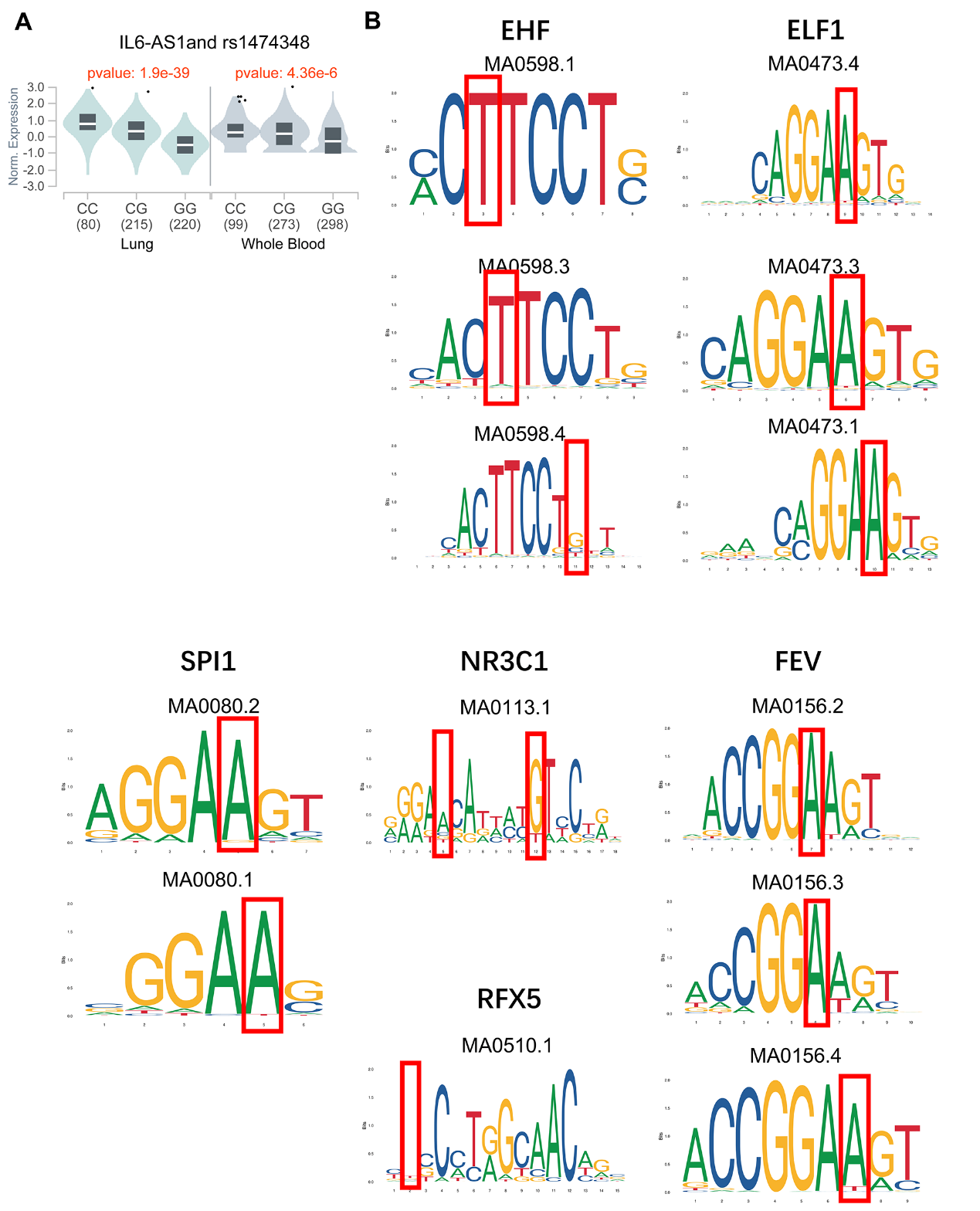


**Figure S24**

**A** Correlation of allelic and replacement bases at rs1474248 with IL6-AS1 expression in lung and whole blood according to the GTEx database.

**B** Binding sites of EHF, ELF1, RFX5, SPI1, NR3C1, and FEV in the JASPAR database. The red box indicates the binding site at rs2069832.

Data are presented as mean ± SD. *P values* in charts were determined by one-way ANOVA Bonferroni's multiple comparisons test (A)


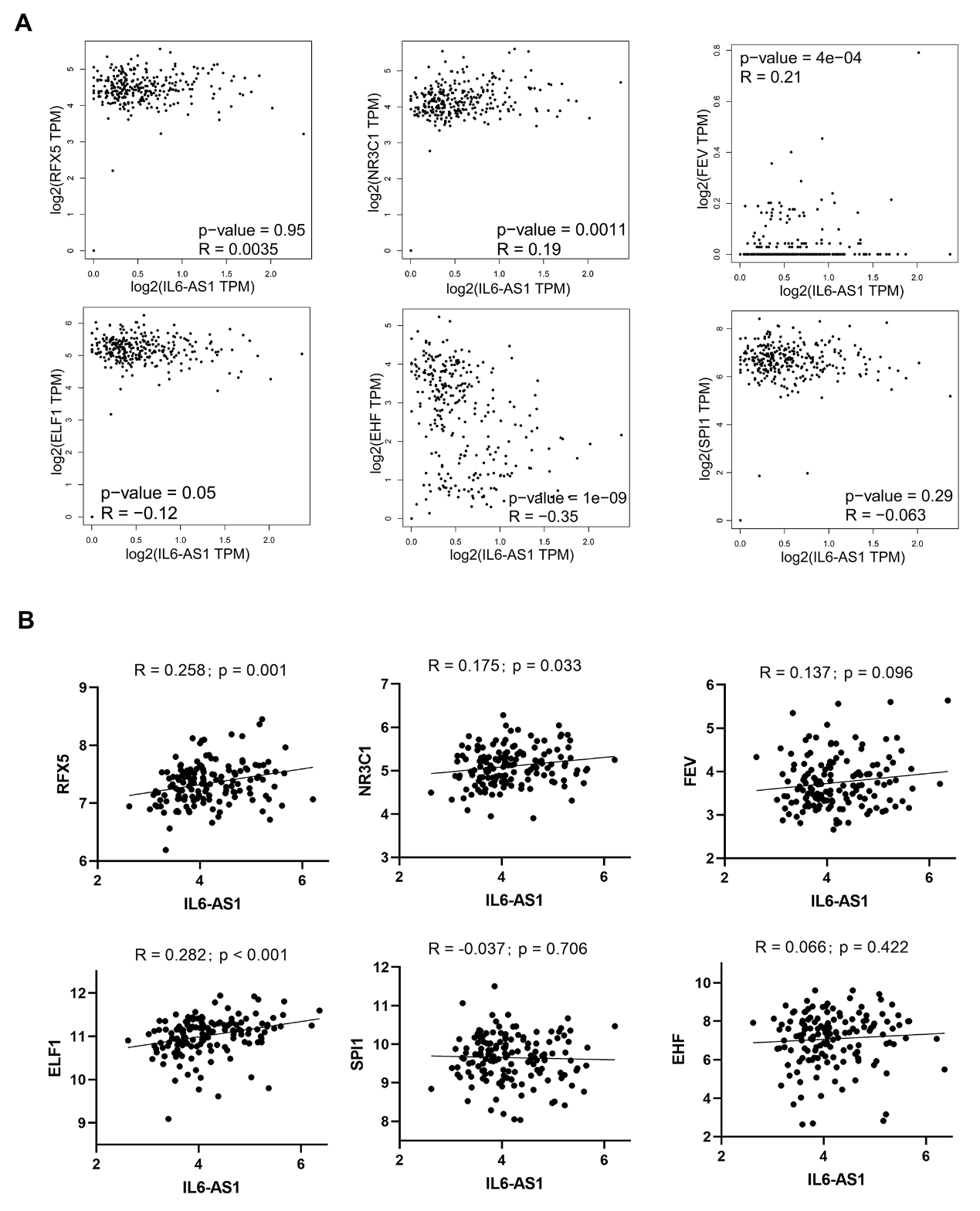


**Figure S25**

**A** Correlation between EHF, ELF1, RFX5, SPI1, NR3C1, and FEV genes with IL6-AS1 expression in lung tissue, as analyzed in the GEPIA database.

**B** Correlation of EHF, ELF1, RFX5, SPI1, NR3C1, and FEV genes with IL6-AS1 expression in lung tissue from the GSE76925 dataset.

*P values* shown in charts determined by Pearson Correlation two-tailed (A, B).

**Supplemental Tables**

**Table S1.** COPD-related gene changes between IL6-AS1 and wild-type smoke-exposed mice

| Gene Symbol | | logFC | | P.Value |
| --- | --- | --- | --- | --- |
| IL6 | | 1.894639 | 0.002175 | |
| TNF | 0.448012 | | 0.049233 | |
| CCL7 | 0.994675 | | 0.005039 | |
| CCL2 | 0.671354 | | 0.027593 | |
| CXCL15 | 1.127956 | | 0.000519 | |
| CXCL3 | 0.878643 | | 0.010163 | |
| ICAM1 | 0.308397 | | 0.042972 | |
| MMP12 | 0.647333 | | 0.058367 | |
| CSF3 | 1.841065 | | 0.006819 | |
| ELN1 | -0.11018 | | 0.452856 | |

**Table S2.** Specific binding proteins for IL6-AS1 identified by ChIRP-MS

| Coverage (%) | Coverage (%) Target | #Peptides | #Unique | Gene Symbol |
| --- | --- | --- | --- | --- |
| 52 | 52 | 15 | 14 | UBA52 |
| 43 | 43 | 15 | 14 | RPS27A |
| 15 | 15 | 12 | 2 | ALB |
| 11 | 11 | 13 | 1 | KRT3 |
| 10 | 10 | 7 | 1 | KRT28 |
| 2 | 2 | 6 | 3 | FLNC |
| 2 | 2 | 6 | 5 | TLN1 |
| 2 | 2 | 3 | 2 | MYH9 |
| 4 | 4 | 5 | 1 | KRT74 |
| 2 | 2 | 4 | 2 | VCL |
| 7 | 7 | 3 | 1 | INA |
| 8 | 8 | 4 | 1 | KRT4 |
| 10 | 10 | 2 | 2 | HIST1H1B |
| 4 | 4 | 3 | 3 | NCL |
| 10 | 10 | 2 | 2 | TAGLN |
| 5 | 5 | 2 | 1 | PGK1 |
| 12 | 12 | 3 | 1 | HSP90AB2P |
| 8 | 8 | 3 | 1 | LDHB |
| 3 | 3 | 3 | 1 | ACTN4 |
| 3 | 3 | 3 | 1 | ACTN3 |
| 4 | 4 | 4 | 3 | CLTC |
| 8 | 8 | 2 | 2 | RPL7 |
| 6 | 6 | 2 | 2 | SLC25A5 |
| 4 | 4 | 2 | 2 | XP32 |
| **21** | **21** | **3** | **3** | **S100A9** |
| 3 | 3 | 3 | 1 | HNRNPR |
| 4 | 4 | 3 | 3 | MSN |
| 3 | 3 | 2 | 1 | RPL3 |
| 8 | 8 | 2 | 2 | RPL13A |
| 2 | 2 | 2 | 1 | DPYSL2 |
| 1 | 1 | 2 | 1 | LMO7 |
| 0 | 0 | 2 | 1 | STARD9 |
| 2 | 2 | 2 | 1 | ADARB1 |
| 5 | 5 | 2 | 2 | CLIC4 |
| 1 | 1 | 2 | 1 | EFCAB6 |
| 2 | 2 | 2 | 1 | RASGRP2 |
| 13 | 13 | 2 | 2 | RPS13 |
| 1 | 1 | 2 | 1 | ARHGAP44 |
| 1 | 1 | 2 | 1 | ATAD5 |
| 2 | 2 | 2 | 1 | OFD1 |
| 1 | 1 | 2 | 2 | TBC1D31 |
| 1 | 1 | 2 | 1 | KDM6B |
| 1 | 1 | 2 | 1 | COL12A1 |

**Table S3.** Intersection of differentially expressed genes in IL6-AS1 mice exposed to smoke with genes previously reported in association with COPD.

| **Gene Symbol** | |  | |  |  |  |
| --- | --- | --- | --- | --- | --- | --- |
| IL-6 | CSF3 | | ALB | CXCL10 | MEFG10 | AREG |
| JUN | RAD51 | | IL5 | CCL2 | RET | CTSG |

**Table S4.** The number of IL6-AS1 positive and negative cells in each cellular subgroup from GSE136861

| cluster | IL6-AS1 negative | IL6-AS1 positive |
| --- | --- | --- |
| Macrophages_1 | 14350 | 38 |
| Macrophages_2 | 10431 | 28 |
| Monocytes_1 | 10071 | 6 |
| Tcells | 7982 | 0 |
| Monocytes_2 | 7067 | 4 |
| Macrophages_3 | 6391 | 17 |
| DendriticCell | 4519 | 2 |
| NK | 4399 | 1 |
| AT2 | 3577 | 4 |
| Macrophages_4 | 3377 | 8 |
| Bcells | 2404 | 14 |
| Fibroblast | 1587 | 6 |
| ProliferationCell | 1287 | 9 |
| VasEndo | 1230 | 7 |
| Ciliated | 1134 | 0 |
| Plasma | 751 | 1 |
| LymphEndo | 761 | 5 |
| Multiplet | 745 | 2 |
| MastCell | 740 | 1 |
| AT1 | 563 | 0 |

**Table S5.** Relative DEGs between IL6-AS1 positive and negative cells in macrophages from GSE136861

| Genes | p_value | avg_log_2_FC | pct.1 | pct.2 | pct.1-2 |
| --- | --- | --- | --- | --- | --- |
| CCL2 | 8.72E-05 | 0.782159 | 0.187 | 0.076 | 0.111 |
| CCL18 | 0.043873 | 0.699122 | 0.681 | 0.586 | 0.095 |
| CCL23 | 6.65E-06 | 0.204012 | 0.242 | 0.098 | 0.144 |
| IL6 | 1.33E-09 | 0.367963 | 0.319 | 0.112 | 0.207 |
| CXCL9 | 3.04E-06 | 0.456803 | 0.11 | 0.028 | 0.082 |
| CXCL2 | 0.03471 | 0.099082 | 0.659 | 0.512 | 0.147 |

**Table S6.** **Information of QTL and GWAS datasets.**

| **Type of dataset** | **Data subtype** | **Resource** | **ID** | **Sample size** |
| --- | --- | --- | --- | --- |
| QTL | eQTL | eQTLGen | AC073072.5 | - |
| QTL | eQTL | GTEx_v8 | AC073072.5 | - |
| GWAS summary | FEV1/FVC | IEU OpenGWAS project | GCST007431 | 321,047 |
| GWAS summary | FEV1 | IEU OpenGWAS project | GCST007432 | 321,047 |
| GWAS summary | FVC | IEU OpenGWAS project | GCST007429 | 321,047 |
| GWAS summary | Diagnosed COPD | IEU OpenGWAS project | ukb-b-20464 | 321,047 |

**Table S7.** **SMR results of the association between expression of IL6-AS in lung and whole blood and COPD outcomes.**

| **Consortium** | **ID** | **Outcome** | **topSNP** | **A1** | **A2** | **Freq** |
| --- | --- | --- | --- | --- | --- | --- |
| GTEx.v8_lung | ukb-b-20464 | Doctor diagnosed COPD | rs1474348 | G | C | 0.5894 |
| GTEx.v8_lung | GCST007429 | FVC | rs1474348 | G | C | 0.5894 |
| GTEx.v8_lung | GCST007431 | FEV_1_/FVC | rs1474348 | G | C | 0.5894 |
| GTEx.v8_lung | GCST007432 | FEV_1_ | rs1474348 | G | C | 0.5894 |
| eQTLGen | ukb-b-20464 | Doctor diagnosed COPD | rs2069832 | A | G | 0.4105 |
| eQTLGen | GCST007429 | FVC | rs2069832 | A | G | 0.4105 |
| eQTLGen | GCST007431 | FEV_1_/FVC | rs2069832 | A | G | 0.4105 |
| eQTLGen | GCST007432 | FEV_1_ | rs2069832 | A | G | 0.4105 |

| **b_GWAS** | **se_GWAS** | **b_eQTL** | **se_eQTL** | **b_SMR** | **se_SMR** | **p_SMR** | **p_HEIDI** | **nsnp_HEIDI** |
| --- | --- | --- | --- | --- | --- | --- | --- | --- |
| -0.0007 | 0.0005 | -0.6136 | 0.04669 | 0.0012 | 0.0008 | 0.1518 | 0.510 | 20 |
| 0.008 | 0.0024 | -0.6136 | 0.04669 | -0.0123 | 0.0040 | 0.0020 | 0.529 | 20 |
| -0.0008 | 0.0024 | -0.6136 | 0.04669 | 0.0013 | 0.0039 | 0.7389 | 0.202 | 20 |
| 0.0065 | 0.0023 | -0.6136 | 0.04669 | -0.0105 | 0.0038 | 0.0057 | 0.242 | 20 |
| 0.0007 | 0.0005 | 0.0901 | 0.0104 | 0.0085 | 0.0057 | 0.1373 | 0.8355 | 8 |
| -0.0076 | 0.0024 | 0.09011 | 0.0104 | -0.0843 | 0.0283 | 0.0029 | 0.5920 | 10 |
| 0.0007 | 0.0024 | 0.09011 | 0.0104 | 0.0077 | 0.0266 | 0.7707 | 0.0571 | 10 |
| -0.0064 | 0.0023 | 0.09011 | 0.0104 | -0.0710 | 0.0268 | 0.0081 | 0.6639 | 20 |

**Table S8.** **Sensitivity analysis used TwosampleMR package on the association between expression of IL6-AS1 and lung function traits (FEV1, FEV1/FVC and FVC) and** **Doctor diagnosed COPD (outcome).**

| **id.exposure** | **id.outcome** | **method** | **nsnp** | **b** | **se** | **pval** |
| --- | --- | --- | --- | --- | --- | --- |
| IL6-AS1_GTEx.v8_lung | ukb-b-20464 | MR Egger | 85 | 0.0013799 | 0.0004993 | 0.0070387 |
| IL6-AS1_GTEx.v8_lung | ukb-b-20464 | Weighted median | 85 | 0.0011964 | 0.0001691 | 0 |
| IL6-AS1_GTEx.v8_lung | ukb-b-20464 | Inverse variance weighted | 85 | 0.001267 | 0.0001284 | 0 |
| IL6-AS1_GTEx.v8_lung | ukb-b-20464 | Simple mode | 85 | 0.0012332 | 0.0003176 | 0.000205 |
| IL6-AS1_GTEx.v8_lung | ukb-b-20464 | Weighted mode | 85 | 0.0012146 | 0.0002832 | 0.0000477 |
| IL6-AS1_GTEx.v8_lung | ukb-b-20464 | MR-PRESSO | NA | NA | NA | 1 |
| IL6-AS1_GTEx.v8_lung | GCST007429 | MR Egger | 98 | -0.0108279 | 0.0022896 | 7.70E-06 |
| IL6-AS1_GTEx.v8_lung | GCST007429 | Weighted median | 98 | -0.0133141 | 0.0007857 | 0 |
| IL6-AS1_GTEx.v8_lung | GCST007429 | Inverse variance weighted | 98 | -0.0147115 | 0.0005806 | 0 |
| IL6-AS1_GTEx.v8_lung | GCST007429 | Simple mode | 98 | -0.0134717 | 0.001464 | 0 |
| IL6-AS1_GTEx.v8_lung | GCST007429 | Weighted mode | 98 | -0.0132785 | 0.0012791 | 0 |
| IL6-AS1_GTEx.v8_lung | GCST007429 | MR-PRESSO | NA | NA | NA | 1 |
| IL6-AS1_GTEx.v8_lung | GCST007432 | MR Egger | 98 | -0.0056925 | 0.0023357 | 0.0166423 |
| IL6-AS1_GTEx.v8_lung | GCST007432 | Weighted median | 98 | -0.0108357 | 0.0008343 | 0 |
| IL6-AS1_GTEx.v8_lung | GCST007432 | Inverse variance weighted | 98 | -0.012893 | 0.0006198 | 0 |
| IL6-AS1_GTEx.v8_lung | GCST007432 | Simple mode | 98 | -0.0111078 | 0.0014127 | 0 |
| IL6-AS1_GTEx.v8_lung | GCST007432 | Weighted mode | 98 | -0.0109946 | 0.0011121 | 0 |
| IL6-AS1_GTEx.v8_lung | GCST007432 | MR-PRESSO | NA | NA | NA | 1.56E-37 |
| IL6-AS1_GTEx.v8_lung | GCST007431 | MR Egger | 95 | 0.0083415 | 0.0023377 | 0.0005706 |
| IL6-AS1_GTEx.v8_lung | GCST007431 | Weighted median | 95 | 0.002395 | 0.0008456 | 0.0046232 |
| IL6-AS1_GTEx.v8_lung | GCST007431 | Inverse variance weighted | 95 | 0.0010362 | 0.0005938 | 0.0809794 |
| IL6-AS1_GTEx.v8_lung | GCST007431 | Simple mode | 95 | 0.0023287 | 0.0015581 | 0.1383932 |
| IL6-AS1_GTEx.v8_lung | GCST007431 | Weighted mode | 95 | 0.0023973 | 0.0012231 | 0.0529562 |
| IL6-AS1_GTEx.v8_lung | GCST007431 | MR-PRESSO | NA | NA | NA | 0.0406 |
| IL6-AS1_eQTLGen | ukb-b-20464 | MR Egger | 45 | 0.0098382 | 0.0075228 | 0.1978961 |
| IL6-AS1_eQTLGen | ukb-b-20464 | Weighted median | 45 | 0.0082768 | 0.0012668 | 0 |
| IL6-AS1_eQTLGen | ukb-b-20464 | Inverse variance weighted | 45 | 0.0089005 | 0.0009995 | 0 |
| IL6-AS1_eQTLGen | ukb-b-20464 | Simple mode | 45 | 0.0081585 | 0.0024806 | 0.0019839 |
| IL6-AS1_eQTLGen | ukb-b-20464 | Weighted mode | 45 | 0.0081832 | 0.0025012 | 0.0020833 |
| IL6-AS1_eQTLGen | ukb-b-20464 | MR-PRESSO | NA | NA | NA | 1 |
| IL6-AS1_eQTLGen | GCST007429 | MR Egger | 50 | -0.1198202 | 0.0311526 | 0.0003532 |
| IL6-AS1_eQTLGen | GCST007429 | Weighted median | 50 | -0.0927343 | 0.0061139 | 0 |
| IL6-AS1_eQTLGen | GCST007429 | Inverse variance weighted | 50 | -0.0929625 | 0.0045166 | 0 |
| IL6-AS1_eQTLGen | GCST007429 | Simple mode | 50 | -0.0925173 | 0.011742 | 0 |
| IL6-AS1_eQTLGen | GCST007429 | Weighted mode | 50 | -0.0925173 | 0.0114574 | 0 |
| IL6-AS1_eQTLGen | GCST007429 | MR-PRESSO | NA | NA | NA | 1 |
| IL6-AS1_eQTLGen | GCST007432 | MR Egger | 50 | -0.057153 | 0.0310086 | 0.0714892 |
| IL6-AS1_eQTLGen | GCST007432 | Weighted median | 50 | -0.0765892 | 0.0059154 | 0 |
| IL6-AS1_eQTLGen | GCST007432 | Inverse variance weighted | 50 | -0.0801744 | 0.0044822 | 0 |
| IL6-AS1_eQTLGen | GCST007432 | Simple mode | 50 | -0.0757671 | 0.011457 | 0 |
| IL6-AS1_eQTLGen | GCST007432 | Weighted mode | 50 | -0.0754127 | 0.0104465 | 0 |
| IL6-AS1_eQTLGen | GCST007432 | MR-PRESSO | NA | NA | NA | 1 |
| IL6-AS1_eQTLGen | GCST007431 | MR Egger | 50 | 0.1199677 | 0.0319966 | 0.0004767 |
| IL6-AS1_eQTLGen | GCST007431 | Weighted median | 50 | 0.0155422 | 0.0059531 | 0.0090336 |
| IL6-AS1_eQTLGen | GCST007431 | Inverse variance weighted | 50 | 0.0079429 | 0.0045692 | 0.0821473 |
| IL6-AS1_eQTLGen | GCST007431 | Simple mode | 50 | 0.0172799 | 0.0114657 | 0.1382069 |
| IL6-AS1_eQTLGen | GCST007431 | Weighted mode | 50 | 0.0176367 | 0.010527 | 0.1002315 |
| IL6-AS1_eQTLGen | GCST007431 | MR-PRESSO | NA | NA | NA | 0.0257 |

**Table S9.** **Heterogeneity test in inverse variance weight analysis and MR Egger.**

| **id.exposure** | **id.outcome** | **method** | **Q_value** | **degree of freedom** | **p_value** |
| --- | --- | --- | --- | --- | --- |
| IL6-AS1_GTEx.v8_lung | ukb-b-20464 | MR Egger | 21.1001 | 83 | 1 |
| IL6-AS1_GTEx.v8_lung | ukb-b-20464 | Inverse variance weighted | 21.15482 | 84 | 1 |
| IL6-AS1_GTEx.v8_lung | GCST007429 | MR Egger | 102.1201 | 96 | 0.3154 |
| IL6-AS1_GTEx.v8_lung | GCST007429 | Inverse variance weighted | 112.9251 | 97 | 0.1285 |
| IL6-AS1_GTEx.v8_lung | GCST007432 | MR Egger | 56.00069 | 93 | 0.9991 |
| IL6-AS1_GTEx.v8_lung | GCST007432 | Inverse variance weighted | 66.43967 | 94 | 0.9860 |
| IL6-AS1_GTEx.v8_lung | GCST007431 | MR Egger | 63.10117 | 96 | 0.9961 |
| IL6-AS1_GTEx.v8_lung | GCST007431 | Inverse variance weighted | 66.17595 | 97 | 0.9929 |
| IL6-AS1_eQTLGen | ukb-b-20464 | MR Egger | 2.313385 | 43 | 1 |
| IL6-AS1_eQTLGen | ukb-b-20464 | Inverse variance weighted | 2.329201 | 44 | 1 |
| IL6-AS1_eQTLGen | GCST007429 | MR Egger | 12.47015 | 48 | 1 |
| IL6-AS1_eQTLGen | GCST007429 | Inverse variance weighted | 13.0331 | 49 | 0.9999 |
| IL6-AS1_eQTLGen | GCST007432 | MR Egger | 15.49522 | 48 | 0.9999 |
| IL6-AS1_eQTLGen | GCST007432 | Inverse variance weighted | 28.00846 | 49 | 0.9931 |
| IL6-AS1_eQTLGen | GCST007431 | MR Egger | 12.99034 | 48 | 0.9999 |
| IL6-AS1_eQTLGen | GCST007431 | Inverse variance weighted | 13.74958 | 49 | 0.9999 |

**Table S10.** **Test for directional horizontal pleiotropy by the intercept of MR Egger**

| **id.exposure** | **id.outcome** | **egger_intercept** | **se** | **pval** | |
| --- | --- | --- | --- | --- | --- |
| IL6-AS1_GTEx.v8_lung | ukb-b-20464 | -5.18E-05 | 0.0002 | | 0.8156 |
| IL6-AS1_GTEx.v8_lung | GCST007429 | -0.0032 | 0.0010 | | 0.0019 |
| IL6-AS1_GTEx.v8_lung | GCST007432 | -0.0033 | 0.0010 | | 0.0017 |
| IL6-AS1_GTEx.v8_lung | GCST007431 | -0.0017 | 0.0010 | | 0.0827 |
| IL6-AS1_eQTLGen | ukb-b-20464 | -7.26E-05 | 0.0005 | | 0.9005 |
| IL6-AS1_eQTLGen | GCST007429 | -0.0018 | 0.0024 | | 0.4567 |
| IL6-AS1_eQTLGen | GCST007432 | -0.0087 | 0.0024 | | 0.0009 |
| IL6-AS1_eQTLGen | GCST007431 | 0.0021 | 0.0024 | | 0.3879 |

**Table S11. colocalization results of the association between expression of IL6-AS1 and lung function traits (FEV_1_, FEV_1_/FVC and FVC) and Doctor diagnosed COPD (outcome).**

| **id.exposure** | **id.outcome** | **PP.H0** | **PP.H1** | **PP.H2** | **PP.H3** | **PP.H4** |
| --- | --- | --- | --- | --- | --- | --- |
| IL6-AS1_GTEx.v8_lung | ukb-b-20464 | 1.40E-36 | 0.999211 | 7.85E-41 | 5.54E-05 | 0.000733 |
| IL6-AS1_GTEx.v8_lung | GCST007429 | 1.05E-36 | 0.777804 | 4.03E-38 | 0.029621 | 0.192576 |
| IL6-AS1_GTEx.v8_lung | GCST007432 | 1.24E-36 | 0.908084 | 2.28E-38 | 0.016602 | 0.075314 |
| IL6-AS1_GTEx.v8_lung | GCST007431 | 1.41E-36 | 0.997917 | 4.50E-40 | 0.000318 | 0.001766 |
| IL6-AS1_eQTLGen | ukb-b-20464 | 2.96E-12 | 0.999233 | 1.03E-16 | 3.39E-05 | 0.000733 |
| IL6-AS1_eQTLGen | GCST007429 | 2.31E-12 | 0.779838 | 5.23E-14 | 0.017443 | 0.202719 |
| IL6-AS1_eQTLGen | GCST007432 | 2.72E-12 | 0.917733 | 1.78E-14 | 0.005941 | 0.076326 |
| IL6-AS1_eQTLGen | GCST007431 | 2.96E-12 | 0.998053 | 5.07E-16 | 0.000169 | 0.001778 |

**Table S12. colocalization results of the top 2 SNPs between expression of IL6-AS1 and lung function traits (FEV_1_, FEV_1_/FVC and FVC) and Doctor diagnosed COPD (outcome).**

| **id.exposure** | **id.outcome** | **snp** | **SNP.PP.H4** |
| --- | --- | --- | --- |
| IL6-AS1_GTEx.v8_lung | ukb-b-20464 | rs2069832 | 0.278519799 |
| IL6-AS1_GTEx.v8_lung | ukb-b-20464 | rs1474348 | 0.252397319 |
| IL6-AS1_GTEx.v8_lung | GCST007429 | rs2069832 | 0.264614284 |
| IL6-AS1_GTEx.v8_lung | GCST007429 | rs1474348 | 0.264614284 |
| IL6-AS1_GTEx.v8_lung | GCST007432 | rs1474348 | 0.275985033 |
| IL6-AS1_GTEx.v8_lung | GCST007432 | rs2069832 | 0.244313382 |
| IL6-AS1_GTEx.v8_lung | GCST007431 | rs1474348 | 0.256245735 |
| IL6-AS1_GTEx.v8_lung | GCST007431 | rs2069832 | 0.252931757 |
| IL6-AS1_eQTLGen | ukb-b-20464 | rs2069832 | 0.292540708 |
| IL6-AS1_eQTLGen | ukb-b-20464 | rs1474348 | 0.193359036 |
| IL6-AS1_eQTLGen | GCST007429 | rs2069832 | 0.264518584 |
| IL6-AS1_eQTLGen | GCST007429 | rs1474348 | 0.157528658 |
| IL6-AS1_eQTLGen | GCST007432 | rs2069832 | 0.254839684 |
| IL6-AS1_eQTLGen | GCST007432 | rs1474348 | 0.197760139 |
| IL6-AS1_eQTLGen | GCST007431 | rs2069832 | 0.264185918 |
| IL6-AS1_eQTLGen | GCST007431 | rs1474347 | 0.209864041 |

**Table S13. Correlation of rs2069832 and rs1474348 with phenotypes in GWAS datasets**

| SNP | Dataset | Phenotype | Pvalue | Beta | Number |
| --- | --- | --- | --- | --- | --- |
| rs1474348 | GWAS_SpiroMeta_eu_UKBiobank | FVC | 0.0049 | 0.0076 | 321047 |
| rs1474348 | GWAS_SpiroMeta_eu_UKBiobank | FEV1 | 0.0189 | 0.0064 | 321047 |
| rs1474348 | GWAS_SpiroMeta_eu | PEF | 0.2340 | 0.0030 | 345265 |
| rs1474348 | Zhou2022_COPD_Mixed | COPD | 0.0571 | -0.021 | 108542 |
| rs1474348 | GWAS_SpiroMeta_eu_UKBiobank | FEV1 to FVC | 0.8130 | -9E-04 | 321047 |
| rs2069832 | GWAS_SpiroMeta_eu_UKBiobank | FVC | 0.0013 | 0.0076 | 403911 |
| rs2069832 | GWAS_SpiroMeta_eu_UKBiobank | FEV1 | 0.0062 | 0.0064 | 404164 |
| rs2069832 | GWAS_SpiroMeta_eu | PEF | 0.1490 | 0.0038 | 321047 |
| rs2069832 | Zhou2022_COPD_Mixed | COPD | 0.0389 | -0.165 | 7384 |
| rs2069832 | GWAS_SpiroMeta_eu_UKBiobank | FEV1 to FVC | 0.76 | -7E-04 | 404127 |

**Table S14. TFs containing the rs2069832 site were predicted using the EnhancerDB database**

| **Chrom.** | **Start** | **End** | **TF name** | **Source** | **id** |
| --- | --- | --- | --- | --- | --- |
| chr7 | 22767419 | 22767434 | RFX5 | JASPAR | 13480559 |
| chr7 | 22767423 | 22767436 | ELF1 | JASPAR | 13480560 |
| chr7 | 22767426 | 22767444 | NR3C1 | JASPAR | 13480561 |
| chr7 | 22767427 | 22767435 | EHF | JASPAR | 13480562 |
| chr7 | 22767427 | 22767435 | FEV | JASPAR | 13480563 |
| chr7 | 22767428 | 22767435 | SPI1 | JASPAR | 13480564 |

**Table S15.** JASPAR predicted binding sites of transcription factors EHF, ELF1, SPI1, NR3C1, RFX5, and FEV within 2000bp upstream of IL6-AS1

| **Matrix ID** | **Score** | **Relative score** | **Start** | **End** | **Strand** | **Predicted sequence** |
| --- | --- | --- | --- | --- | --- | --- |
| MA0598.1 | MA0598.1.EHF | 13.78529 | 0.999252193 | 1805 | 1812 | + |
| MA0598.4 | MA0598.4.EHF | 13.424381 | 0.957020636 | 1804 | 1812 | + |
| MA0473.4 | MA0473.4.ELF1 | 12.868008 | 0.93128909 | 1804 | 1812 | - |
| MA0473.3 | MA0473.3.ELF1 | 11.717934 | 0.872815284 | 1802 | 1815 | - |
| MA0598.3 | MA0598.3.EHF | 11.563081 | 0.865757561 | 1802 | 1816 | + |
| MA0080.2 | MA0080.2.SPI1 | 11.542796 | 1.00000001 | 1805 | 1811 | - |
| MA0473.1 | MA0473.1.ELF1 | 10.626096 | 0.892233609 | 1804 | 1816 | - |
| MA0510.1 | MA0510.1.RFX5 | 10.392226 | 0.892663452 | 1806 | 1820 | + |
| MA0113.1 | MA0113.1.NR3C1 | 9.768812 | 0.824618422 | 1796 | 1813 | + |
| MA0156.2 | MA0156.2.FEV | 9.376312 | 0.895559538 | 1804 | 1813 | - |
| MA0080.2 | MA0080.2.SPI1 | 8.637205 | 0.903184569 | 1801 | 1807 | + |
| MA0113.1 | MA0113.1.NR3C1 | 8.374595 | 0.797310755 | 1794 | 1811 | - |
| MA0080.1 | MA0080.1.SPI1 | 8.184016 | 0.977897674 | 1806 | 1811 | - |
| MA0598.4 | MA0598.4.EHF | 6.641159 | 0.815204276 | 1800 | 1808 | - |
| MA0156.4 | MA0156.4.FEV | 6.525455 | 0.847700686 | 1805 | 1813 | - |
| MA0156.3 | MA0156.3.FEV | 6.044614 | 0.827290361 | 1803 | 1814 | - |
| MA0156.2 | MA0156.2.FEV | 4.6779256 | 0.816830112 | 1799 | 1808 | + |

**Table S16.** **Primers used in experiments.**

| **Primer name** | **Forward primer (5’-3’)** | **Reverse primer (5’-3’)** | **Application** |
| --- | --- | --- | --- |
| IL6-AS1  GAPDH  IL-6 | GCATAACATTTCAGGACCCGC CAGCCTCAAGATCATCAGCA  AAGCCAGAGCTGTGCAGATGAGTA | GGAGCAGTGGCTTCGTTTCA ACAGTCTTCTGGGTGGCAGT  TGTCCTGCAGCCACTGGTTC | qRT-PCR  qRT-PCR  qRT-PCR |
| IL-8 | AACTGAGAGTGATTGAGAGTGG | ATGAATTCTCAGCCCTCTTCAA | qRT-PCR |
| CCL2  TNF | CTTCTGTGCCTGCTGCTCATA AGCGGTTGTGAGGAGTTAGC | CTTTGGGACACTTGCTGCTG CATGCCTGGACACTCACCC | qRT-PCR  qRT-PCR |
| CCL7 | AGCTGCTTTCAGCCCCCAG | ACTTCTGTGTGGGGTCAGCAC | qRT-PCR |
| CXCL3 | TTTTCAGCGGATAGCTGAGGC | ATGGGGACTGAGCTGTTTCTT | qRT-PCR |
| CXCL10 | GAAGAGAGACTGACTGGGCAAC | CACCCCTCTGGAATCCACTTAT | qRT-PCR |
| CD86 | GCCTTAGGAGGTACGGGGA | CAGCACCAGAGAGCAGGAAG | qRT-PCR |
| CD11b | TTGGTGGCTTCCTTGTGGTT | CCCCTTGCGTTCTCTTGGAA | qRT-PCR |
| IL-10 | GGGCACCCAGTCTGAGAAC | GGCATCACCTCCTCCAGGTA | qRT-PCR |
| CD206 | ACCTGCGACAGTAAACGAGG | TGTCTCCGCTTCATGCCATT | qRT-PCR |
| ICAM1 | CGGTCGGGTTGGTTCTTACA | CTGGTCTGCAGAGAGCGAAT | qRT-PCR |
| Mus-IL-6 | AGCCAGAGTCCTTCAGAGAGAT | AGGAGAGCATTGGAAATTGGGG | qRT-PCR |
| Mus-CXCL15 (IL-8) | CTAGGCATCTTCGTCCGTCC | TTCACCCATGGAGCATCAGG | qRT-PCR |
| Mus-MCP-1 | TGAGGAGGAGCCAGGAAGG | GCTCAAGCTCCACTTCCTCA | qRT-PCR |
| Mus-CCL2 | CTGCTGTTCACAGTTGCCG | GCACAGACCTCTCTCTTGAGC | qRT-PCR |
| Mus-CXCL3 | TGCACCCAGACAGAAGTCATAG | GTTGGGATGGATCGCTTTTCTC | qRT-PCR |
| Mus-CXCL10 | CCTGCAAGCCAATTTTGTCCA | TGTGGTCCATCCTTGGAAGC | qRT-PCR |
| Mus-CCL7 | GATCTCTGCCACGCTTCTGT | ATAGCCTCCTCGACCCACTT | qRT-PCR |
| Mus-TNF | CCTGTAGCCCACGTCGTAG | GGGAGTAGACAAGGTACAACCC | qRT-PCR |
| Mus-MMP12  Mus-ELN  Mus-ICAM1  MMP12  Mus-CFS3 | TGTGTTCTTACAGGTATCTGCC TGTCCCACTGGGTTATCCCAT  GTCCGCTGTGCTTTGAGAAC CCAGTTTTCCGAGAACCAAA  GCACTATGGTCAGGACGAGAG | TGACAAGTACCATTCAGCAAATTCA CAGCTACTCCATAGGGCAATTTC  GAGGTCCTTGCCTACTTGCT ATGCTGATCTGCTGCGTATG  GGGGAAATACCCGATAGAGCC | qRT-PCR  qRT-PCR  qRT-PCR  qRT-PCR  qRT-PCR |
| Mus-ACTIN | AGTGTGACGTTGACATCCGT | GTAACAGTCCGCCTAGAAGC | qRT-PCR |
| U1 | TCAAGAAGGATGCACCCCCA | ATAATACGCCCGAGTTCCCC | qRT-PCR |
| ACTIN | CTGTCCAACATGATCGTGCG | GACACAGAGATCCGCAGTCC | qRT-PCR |

**Reference:**

1. Yi E, Lin B, Zhang Y, Wang X, Zhang J, Liu Y, Jin J, Hong W, Lin Z, Cao W *et al*: **Smad3-mediated lncRNA HSALR1 enhances the non-classic signalling pathway of TGF-beta1 in human bronchial fibroblasts by binding to HSP90AB1**. *Clin Transl Med* 2023, **13**(6):e1292.

2. Yi E, Cao W, Zhang J, Lin B, Wang Z, Wang X, Bai G, Mei X, Xie C, Jin J *et al*: **Genetic screening of MMP1 as a potential pathogenic gene in chronic obstructive pulmonary disease**. *Life Sci* 2023, **313**:121214.

3. Yi E, Zhang J, Zheng M, Zhang Y, Liang C, Hao B, Hong W, Lin B, Pu J, Lin Z *et al*: **Long noncoding RNA IL6-AS1 is highly expressed in chronic obstructive pulmonary disease and is associated with interleukin 6 by targeting miR-149-5p and early B-cell factor 1**. *Clin Transl Med* 2021, **11**(7):e479.

4. Tang Z, Kang B, Li C, Chen T, Zhang Z: **GEPIA2: an enhanced web server for large-scale expression profiling and interactive analysis**. *Nucleic Acids Res* 2019, **47**(W1):W556-W560.

5. Castro-Mondragon JA, Riudavets-Puig R, Rauluseviciute I, Lemma RB, Turchi L, Blanc-Mathieu R, Lucas J, Boddie P, Khan A, Manosalva Perez N *et al*: **JASPAR 2022: the 9th release of the open-access database of transcription factor binding profiles**. *Nucleic Acids Res* 2022, **50**(D1):D165-D173.

6. Nassar LR, Barber GP, Benet-Pages A, Casper J, Clawson H, Diekhans M, Fischer C, Gonzalez JN, Hinrichs AS, Lee BT *et al*: **The UCSC Genome Browser database: 2023 update**. *Nucleic Acids Res* 2023, **51**(D1):D1188-D1195.

7. Kang R, Zhang Y, Huang Q, Meng J, Ding R, Chang Y, Xiong L, Guo Z: **EnhancerDB: a resource of transcriptional regulation in the context of enhancers**. *Database (Oxford)* 2019, **2019**.

8. Lowe KE, Regan EA, Anzueto A, Austin E, Austin JHM, Beaty TH, Benos PV, Benway CJ, Bhatt SP, Bleecker ER *et al*: **COPDGene((R)) 2019: Redefining the Diagnosis of Chronic Obstructive Pulmonary Disease**. *Chronic Obstr Pulm Dis* 2019, **6**(5):384-399.

9. Sun R, Liu Y, Lu M, Ding Q, Wang P, Zhang H, Tian X, Lu P, Meng D, Sun N *et al*: **ALIX increases protein content and protective function of iPSC-derived exosomes**. *J Mol Med (Berl)* 2019, **97**(6):829-844.

10. Watanabe N, Fujita Y, Nakayama J, Mori Y, Kadota T, Hayashi Y, Shimomura I, Ohtsuka T, Okamoto K, Araya J *et al*: **Anomalous Epithelial Variations and Ectopic Inflammatory Response in Chronic Obstructive Pulmonary Disease**. *Am J Respir Cell Mol Biol* 2022, **67**(6):708-719.

11. Adams TS, Schupp JC, Poli S, Ayaub EA, Neumark N, Ahangari F, Chu SG, Raby BA, DeIuliis G, Januszyk M *et al*: **Single-cell RNA-seq reveals ectopic and aberrant lung-resident cell populations in idiopathic pulmonary fibrosis**. *Sci Adv* 2020, **6**(28):eaba1983.

12. Jin S, Guerrero-Juarez CF, Zhang L, Chang I, Ramos R, Kuan CH, Myung P, Plikus MV, Nie Q: **Inference and analysis of cell-cell communication using CellChat**. *Nat Commun* 2021, **12**(1):1088.

13. Kim S, Herazo-Maya JD, Kang DD, Juan-Guardela BM, Tedrow J, Martinez FJ, Sciurba FC, Tseng GC, Kaminski N: **Integrative phenotyping framework (iPF): integrative clustering of multiple omics data identifies novel lung disease subphenotypes**. *BMC Genomics* 2015, **16**:924.

14. Morrow JD, Zhou X, Lao T, Jiang Z, DeMeo DL, Cho MH, Qiu W, Cloonan S, Pinto-Plata V, Celli B *et al*: **Functional interactors of three genome-wide association study genes are differentially expressed in severe chronic obstructive pulmonary disease lung tissue**. *Sci Rep* 2017, **7**:44232.

15. Cruz T, Lopez-Giraldo A, Noell G, Guirao A, Casas-Recasens S, Garcia T, Saco A, Sellares J, Agusti A, Faner R: **Smoking Impairs the Immunomodulatory Capacity of Lung-Resident Mesenchymal Stem Cells in Chronic Obstructive Pulmonary Disease**. *Am J Respir Cell Mol Biol* 2019, **61**(5):575-583.

16. Bhattacharya S, Srisuma S, Demeo DL, Shapiro SD, Bueno R, Silverman EK, Reilly JJ, Mariani TJ: **Molecular biomarkers for quantitative and discrete COPD phenotypes**. *Am J Respir Cell Mol Biol* 2009, **40**(3):359-367.

17. Shaykhiev R, Krause A, Salit J, Strulovici-Barel Y, Harvey BG, O'Connor TP, Crystal RG: **Smoking-dependent reprogramming of alveolar macrophage polarization: implication for pathogenesis of chronic obstructive pulmonary disease**. *J Immunol* 2009, **183**(4):2867-2883.

18. Steiling K, van den Berge M, Hijazi K, Florido R, Campbell J, Liu G, Xiao J, Zhang X, Duclos G, Drizik E *et al*: **A dynamic bronchial airway gene expression signature of chronic obstructive pulmonary disease and lung function impairment**. *Am J Respir Crit Care Med* 2013, **187**(9):933-942.

19. O'Beirne SL, Kikkers SA, Oromendia C, Salit J, Rostmai MR, Ballman KV, Kaner RJ, Crystal RG, Cloonan SM: **Alveolar Macrophage Immunometabolism and Lung Function Impairment in Smoking and Chronic Obstructive Pulmonary Disease**. *Am J Respir Crit Care Med* 2020, **201**(6):735-739.

20. Samaha E, Vierlinger K, Weinhappel W, Godnic-Cvar J, Nohammer C, Koczan D, Thiesen HJ, Yanai H, Fraifeld VE, Ziesche R: **Expression Profiling Suggests Loss of Surface Integrity and Failure of Regenerative Repair as Major Driving Forces for Chronic Obstructive Pulmonary Disease Progression**. *Am J Respir Cell Mol Biol* 2021, **64**(4):441-452.

21. Hemani G, Zheng J, Elsworth B, Wade KH, Haberland V, Baird D, Laurin C, Burgess S, Bowden J, Langdon R *et al*: **The MR-Base platform supports systematic causal inference across the human phenome**. *Elife* 2018, **7**.

22. Sudlow C, Gallacher J, Allen N, Beral V, Burton P, Danesh J, Downey P, Elliott P, Green J, Landray M *et al*: **UK biobank: an open access resource for identifying the causes of a wide range of complex diseases of middle and old age**. *PLoS Med* 2015, **12**(3):e1001779.

23. Shrine N, Guyatt AL, Erzurumluoglu AM, Jackson VE, Hobbs BD, Melbourne CA, Batini C, Fawcett KA, Song K, Sakornsakolpat P *et al*: **New genetic signals for lung function highlight pathways and chronic obstructive pulmonary disease associations across multiple ancestries**. *Nat Genet* 2019, **51**(3):481-493.

24. Oliva M, Munoz-Aguirre M, Kim-Hellmuth S, Wucher V, Gewirtz ADH, Cotter DJ, Parsana P, Kasela S, Balliu B, Vinuela A *et al*: **The impact of sex on gene expression across human tissues**. *Science* 2020, **369**(6509).

25. Wray NR, Ripke S, Mattheisen M, Trzaskowski M, Byrne EM, Abdellaoui A, Adams MJ, Agerbo E, Air TM, Andlauer TMF *et al*: **Genome-wide association analyses identify 44 risk variants and refine the genetic architecture of major depression**. *Nat Genet* 2018, **50**(5):668-681.

26. Zhu Z, Zhang F, Hu H, Bakshi A, Robinson MR, Powell JE, Montgomery GW, Goddard ME, Wray NR, Visscher PM *et al*: **Integration of summary data from GWAS and eQTL studies predicts complex trait gene targets**. *Nat Genet* 2016, **48**(5):481-487.

27. Burgess S, Thompson SG: **Multivariable Mendelian randomization: the use of pleiotropic genetic variants to estimate causal effects**. *Am J Epidemiol* 2015, **181**(4):251-260.

28. Giambartolomei C, Vukcevic D, Schadt EE, Franke L, Hingorani AD, Wallace C, Plagnol V: **Bayesian test for colocalisation between pairs of genetic association studies using summary statistics**. *PLoS Genet* 2014, **10**(5):e1004383.
